# The *Xanthomonas* type-III effector XopS stabilizes *Ca*WRKY40a to regulate defense responses and stomatal immunity in pepper (*Capsicum annuum*)

**DOI:** 10.1093/plcell/koac032

**Published:** 2022-02-02

**Authors:** Margot Raffeiner, Suayib Üstün, Tiziana Guerra, Daniela Spinti, Maria Fitzner, Sophia Sonnewald, Susanne Baldermann, Frederik Börnke

**Affiliations:** Leibniz-Institute of Vegetable and Ornamental Crops (IGZ), Großbeeren 14979, Germany; Leibniz-Institute of Vegetable and Ornamental Crops (IGZ), Großbeeren 14979, Germany; Leibniz-Institute of Vegetable and Ornamental Crops (IGZ), Großbeeren 14979, Germany; Leibniz-Institute of Vegetable and Ornamental Crops (IGZ), Großbeeren 14979, Germany; Institute of Biochemistry and Biology, University of Potsdam, Potsdam 14476, Germany; Leibniz-Institute of Vegetable and Ornamental Crops (IGZ), Großbeeren 14979, Germany; Department of Biology, Division of Biochemistry, Friedrich-Alexander-Universität, Erlangen 91058, Germany; Leibniz-Institute of Vegetable and Ornamental Crops (IGZ), Großbeeren 14979, Germany; Department of Food Chemistry, Institute of Nutritional Science, University of Potsdam, Nuthetal 14558, Germany; Leibniz-Institute of Vegetable and Ornamental Crops (IGZ), Großbeeren 14979, Germany; Institute of Biochemistry and Biology, University of Potsdam, Potsdam 14476, Germany

## Abstract

As a critical part of plant immunity, cells that are attacked by pathogens undergo rapid transcriptional reprogramming to minimize virulence. Many bacterial phytopathogens use type III effector (T3E) proteins to interfere with plant defense responses, including this transcriptional reprogramming. Here, we show that Xanthomonas outer protein S (XopS), a T3E of *Xanthomonas campestris* pv. *vesicatoria* (*Xcv*), interacts with and inhibits proteasomal degradation of WRKY40, a transcriptional regulator of defense gene expression. Virus-induced gene silencing of *WRKY40* in pepper (*Capsicum annuum*) enhanced plant tolerance to *Xcv* infection, indicating that WRKY40 represses immunity. Stabilization of WRKY40 by XopS reduces the expression of its targets, which include salicylic acid-responsive genes and the jasmonic acid signaling repressor *JAZ8*. *Xcv* bacteria lacking XopS display significantly reduced virulence when surface inoculated onto susceptible pepper leaves. XopS delivery by *Xcv*, as well as ectopic expression of XopS in *Arabidopsis thaliana* or *Nicotiana benthamiana*, prevented stomatal closure in response to bacteria and biotic elicitors. Silencing *WRKY40* in pepper or *N. benthamiana* abolished XopS’s ability to prevent stomatal closure. This suggests that XopS interferes with both preinvasion and apoplastic defense by manipulating WRKY40 stability and downstream gene expression, eventually altering phytohormone crosstalk to promote pathogen proliferation.

## Teaser

The Xanthomonas effector protein XopS enhances disease susceptibility through the repression of defense gene expression and is essential to suppress stomatal immunity.

IN A NUTSHELL **Background:** Like other organisms, plants defend themselves against pathogens by activating immune responses. One of the first lines of plant immunity is to block the entry of bacteria into the leaf through pores in the leaf surface, called stomata. Stomata are essential to the plant, as they mediate the gas exchange required for photosynthesis and regulate transpiration and thus the distribution of water. As part of their defense system, plants recognize invading pathogens and in response close their stomata to prevent further bacterial entry. In addition, the defense response leads to the activation of immunity-related genes that eventually help to fight the pathogen. Many bacteria, like *Xanthomonas campestris* pv. *vesicatoria*, can overcome plant immunity by injecting so-called effector proteins directly into the plant cell. Effectors suppress plant defense at different levels (including expression of immunity genes) and promote disease.

**Question:** We sought to understand how bacterial pathogen effectors suppress plant defense responses to cause disease. Therefore, we aimed to investigate whether bacterial effectors could overcome stomatal defenses by affecting the activation of immunity genes.

**Findings:** We found that the *Xanthomonas campestris* pv. *vesicatoria* effector protein XopS prevents stomata from closing upon pathogen recognition and suppresses the expression of immunity genes. WRKY40, a transcription factor that suppresses defense gene expression, is usually degraded by the plant’s protein degradation machinery upon activation of immunity, but XopS physically interacts with and stabilizes WRKY40. XopS binding to WRKY40 interferes with its degradation and due to the higher amounts of WRKY40 protein, the plant cannot properly activate defense gene expression. This also affects expression of key genes required for stomatal closure and thus the stomata remain open making it much easier for the bacteria to get into the leaf.

**Next steps:** We are working to find the biochemical mechanism by which XopS stabilizes WRKY40. We also want to know whether other *Xanthomonas campestris* pv. *vesicatoria* effector proteins interfere with stomatal immunity.

## Introduction

Plants sense pathogen invasion by perceiving pathogen-derived microbe-associated molecular patterns (MAMPs) and launch defense responses, including inducing large-scale reprogramming of gene expression (Tsuda and Somssich, 2015; Garner et al., 2016; Li et al., 2016). For example, researchers identified ∼4,000 MAMP-responsive genes following infection of *Arabidopsis thaliana* plants with a nonpathogenic *Pseudomonas syringae* pv. *tomato* (*Pst*) strain or treatment of tomato (*Solanum lycopersicum*) with the *Pst* derived MAMP flagellin 22 (flg22) (Rosli et al., 2013; Lewis et al., 2015). The genes induced during MAMP-triggered immunity (MTI) are related to defense responses and biosynthesis of the plant hormone salicylic acid (SA); by contrast, MAMP-triggered immunity also involves suppressing genes associated with photosynthesis and chloroplast function (Rosli et al., 2013; Lewis et al., 2015).

Transcriptional reprogramming during defense against pathogens is governed by interacting networks of transcription factors (TFs) from multiple families and their co-regulatory proteins, which affect TF function through various molecular mechanisms (Moore et al., 2011). In general, these TFs act downstream of MAPK cascades or Ca^2+^ signaling via diverse activation mechanisms to relay MAMP signaling into an appropriate transcriptional response. WRKY family TFs have a prominent role among the TFs that mediate transcriptional reprogramming during MTI in Arabidopsis and other plant species (Pandey and Somssich, 2009; Tsuda and Somssich, 2015; Birkenbihl et al., 2017).

All WRKY TFs have an ∼60 amino acid long WRKY domain that binds to the W-box cis-element ((T)TGAC(C/T)) within the promoters of their target genes. Indeed, putative W-boxes have been identified in the promoter regions of many genes associated with plant biotic stress responses, including those involved in SA biosynthesis, and WRKYs have been described as both transcriptional activators and repressors in these pathways. In addition, WRKYs often form positive or negative regulatory feedback loops via binding to their own promoters (Pandey et al., 2010; Mao et al., 2011). Regulation of WRKY transcriptional activity also occurs at the posttranslational level through phosphorylation or proteasomal protein turnover (Ishihama and Yoshioka, 2012; Matsushita et al., 2013).

In Arabidopsis, the closely related WRKY TFs WRKY18 and WRKY40 have partially redundant functions in negatively regulating resistance to *P. syringae* and the biotrophic fungus *Golovinomyces orontii* (Xu et al., 2006; Pandey et al., 2010). The *wrky40 wrky18* double mutants have enhanced resistance toward both pathogens and undergo massive transcriptional reprogramming during early stages of infection in Arabidopsis (Pandey et al., 2010). Expression of several positive and negative regulators of jasmonic acid (JA) and SA signaling is significantly altered in resistant *wrky18 wrky40* double mutants compared with susceptible wild-type (WT) plants. Chromatin immunoprecipitation-sequencing (ChIP-seq) identified ∼1,400 possible target genes for WRKY40 in Arabidopsis with an enrichment for genes involved in the early processes of MAMP perception and signaling (Birkenbihl et al., 2017).

The Gram-negative bacterium *Xanthomonas campestris* pv. *vesicatoria* (*Xcv*; synonymously designated as *Xanthomonas euvesicatoria*) is the causal agent of bacterial spot disease in pepper (*Capsicum annuum*) and tomato plants (Osdaghi et al., 2021). As for other foliar bacterial pathogens, *Xcv* infection begins with an epiphytic phase when the pathogen arrives on the surface of a healthy leaf. The infection proceeds to a largely endophytic phase accompanied by aggressive bacterial multiplication within the apoplast of infected tissues (McGuire et al., 1991). Invasion of the apoplast occurs through natural openings in the leaf surface, including stomata and hydathodes, and through wounds (Ramos and Volin, 1987).

Stomata play an active role in preinvasion immunity against bacteria through the sensing of MAMPs by pattern-recognition receptors on the surface of guard cells around the stomatal pore (Melotto et al., 2006). This induces stomatal closure to prevent further ingress of bacteria into the apoplast, a mechanism referred to as stomatal immunity (Melotto et al., 2006; Sawinski et al., 2013). In addition to MAMP perception, the plant hormones abscisic acid (ABA), SA, and jasmonoyl isoleucine (JA-Ile) play integral roles in regulating stomatal immunity. Treating tomato plants with ABA to induce stomatal closure prior to surface inoculation with *Xcv* reduces disease incidence and symptom severity (Ramos and Volin, 1987), indicating that stomatal aperture can represent a major limiting factor for disease progression.

Several bacterial pathogens have evolved secreted virulence factors to overcome stomatal immunity, such as phytotoxins and type III secreted effector (T3E) proteins that open the stomatal pore (Melotto et al., 2006; Gimenez-Ibanez et al., 2014; Hurley et al., 2014). The best characterized example of such factors is the small molecule coronatine (COR) produced by *Pst* DC3000 to reopen closed stomata, thereby significantly increasing the number of entry sites for bacterial invasion. COR is a structural mimic of JA-Ile (Staswick and Tiryaki, 2004; Melotto et al., 2006; Okada et al., 2009) and binds to the JA co-receptor CORONATINE INSENSITIVE1 (COI1) (Katsir et al., 2008). When COR binds to COI1, downstream signaling leads to the induction of NAC TFs that repress SA biosynthesis genes and induce SA metabolism genes, thereby suppressing SA accumulation and promoting stomatal opening (Zheng et al., 2012; Du et al., 2014; Gimenez-Ibanez et al., 2017). *Xcv* is not known to produce COR, nor have other virulence factors targeting the stomatal immunity of host plants been described for this pathogen. Thus, whether and how *Xcv* interferes with preinvasive defense responses remains unclear.

The ability of *Xcv* to cause disease is largely dependent on a suite of approximately 35 T3Es, several of which are conserved between different *Xcv* strains or even *Xanthomonas* species and constitute a so-called “core” set of effectors, while others are only found in certain strains (Potnis et al., 2011; Schwartz et al., 2015). Although cellular targets and modes of action are known for only a limited number of *Xcv* effectors, it appears that direct manipulation of host transcription constitutes one of the key mechanisms underpinning bacterial pathogenesis (Boch and Bonas, 2010).

Xanthomonas outer protein S (XopS) is an *Xcv* translocated T3E, originally identified in the genome of *Xcv* strain 85-10 (Schulze et al., 2012; Teper et al., 2016). It is a protein of approximately 34 kDa confined to the Xanthomonads and sharing no obvious sequence similarity with other known proteins. The lack of discernible structural features in XopS has so far precluded any prediction of its function or its biochemical activity. An *XcvΔxopS* deletion strain does display reduced symptom development upon pressure infection of susceptible pepper plants, although lack of the effector protein does not appear to affect bacterial multiplication in infected tissue (Schulze et al., 2012). When expressed in Arabidopsis protoplasts, XopS interferes with the induction of MTI marker gene expression, likely acting downstream of MAP kinase signaling (Schulze et al., 2012; Popov et al., 2016). However, the proteins that XopS targets in vivo during infection remained unknown.

In this study, we show that XopS interferes with preinvasive and postinvasive defense responses and that it represents a major virulence factor that works to overcome stomatal immunity in pepper plants. XopS interacts with the negative regulator of defense gene transcription WRKY40, resulting in a dampening of SA-dependent gene expression in favor of JA-mediated responses. XopS binding to WRKY40 stabilizes the TF by inhibiting its proteasomal degradation, thereby perpetuating its repressor activity to attenuate induction of WRKY40 target genes.

## Results

### *XopS* is a bacterial virulence factor and suppresses plant defense

To assess the contribution of XopS to bacterial virulence in more detail, we first knocked out XopS function in *Xcv* and tested whether this affected bacterial infection. To this end, we constructed an *Xcv* 85-10 *xopS* mutant carrying a 571-bp deletion within the *XopS* coding sequence (CDS; acc. no. XCV0324) and used this to infect susceptible pepper plants by pressure infiltration. Our results confirmed a prior report (Schulze et al., 2012) that the *Xcv*Δ*xopS* strain grew to levels similar to those of the WT *Xcv* strain at 5 days postinoculation (dpi) (Supplemental Figure S1A).

While leaves infected with WT *Xcv* showed tissue collapse and clear signs of necrotic lesions, *Xcv*Δ*xopS* infected tissue displayed reduced signs of chlorosis, and infection with an *Xcv*Δ*xopS(XopS-HA)* complementation strain restored the WT phenotype (Supplemental Figure S1C). XopS-HA expression in the *Xcv*Δ*xopS(XopS-HA)* complementation strain was confirmed by immunoblotting (Supplemental Figure S1B). *Xcv*Δ*xopS* infected plants displayed significantly higher levels of the defense hormone SA relative to plants inoculated with WT *Xcv*, accompanied by elevated expression of the SA-responsive defense gene *CaPR1* (Supplemental Figure S1, D and E). This indicates that XopS interferes with cellular processes that lead to the development of host tissue necrosis and likely interferes with defense hormone balance.

**Figure 1:**
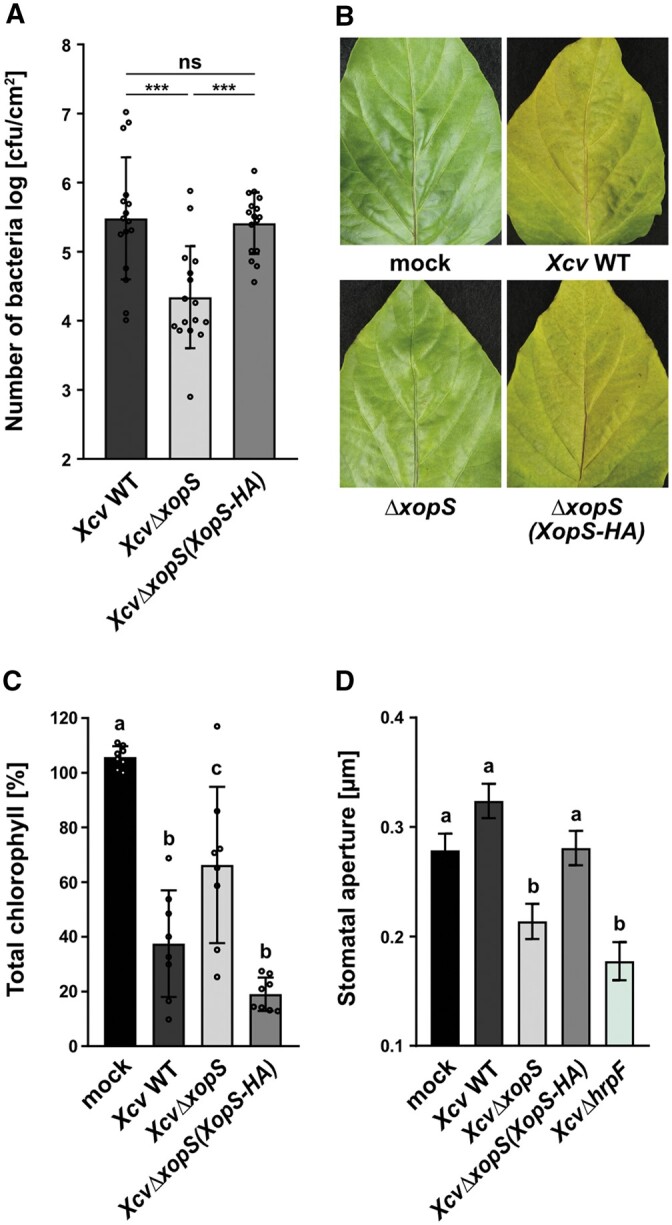
XopS is required for full virulence of *Xcv* on susceptible pepper plants. A, Bacterial multiplication of *Xcv* WT, *XcvΔxopS* and *XcvΔxopS(XopS-HA)* strains in susceptible pepper plants. Leaves were dip-inoculated with a bacterial suspension at OD_600_ = 0.2 and CFUs in infected tissue were quantified at 7 dpi. Bars represent the mean of *n* = 8 biological replicates (and two technical replicates per biological replicate) ± SD. Significant differences are marked by asterisks (***P < 0.001; ns, not significant) according to one-way ANOVA. The experiment was carried out 3 times with similar results. B, Disease symptom development in pepper leaves, dip-inoculated with *Xcv* WT, *XcvΔxopS* or *XcvΔxopS(XopS-HA)* strains. Pictures were taken at 7 dpi. A similar phenotype was consistently observed in three independent experiments (C) Chlorophyll content in pepper leaves, dip-inoculated with *Xcv* strains indicated. Uninfected leaves were used as mock control and the percentage of total chlorophyll was determined when the mock control was set to 100%. The measurement was performed at 7 dpi. Bars represent the mean of *n* = 8 biological replicates ± SD. Letters above bars represent statistical significance determined by one-way ANOVA (*P* < 0.05). The experiment was carried out twice with similar results. D, Stomatal apertures of pepper leaf discs from floated on water (mock) or on water containing *Xcv* WT, *XcvΔxopS*, *XcvΔxopS(XopS-HA) or XcvΔhrpF* strains at an OD_600_ = 0.2. The measurement of stomatal aperture was performed 2 h posttreatment. Approximately 100 apertures from *n* = 4 different plants were measured per individual treatment and are represented as width/length ratio. Bars represent the mean ± SE and letters above bars represent the statistical significance determined by one-way ANOVA (*P* < 0.05). The experiment was carried out twice with similar results.

Pressure infiltration of pathogenic bacteria into susceptible leaf tissue circumvents stomatal defense, a major barrier of the plant immune system that bacteria encounter during natural infection (Melotto et al., 2008). To investigate whether XopS might affect preinvasion defense responses, we dip-inoculated susceptible pepper plants with bacterial suspension cultures (1 × 10^8^ colony forming unit [CFU]/mL). *Xcv*Δ*xopS* bacteria achieved significantly lower population densities than WT bacteria at 7 dpi (Figure 1A). Complementation of the *Xcv*Δ*xopS* strain by ectopic XopS expression fully restored growth to WT levels. As observed upon pressure infiltration, dip-inoculation with the *Xcv*Δ*xopS* strain reduced the severity of symptoms, resulting in higher chlorophyll levels in the leaves when compared to WT *Xcv* or *Xcv*Δ*xopS(XopS-HA)* infected leaves (Figure 1, B and C).

Incubating pepper leaves with WT *Xcv* bacteria for 2 h did not affect stomatal aperture relative to mock-treated leaves (Figure 1D). However, treating leaves either with the *Xcv*Δ*xopS* strain or with an *XcvΔhrpF* strain, which is unable to deliver T3Es into the host cell, induced significant stomatal closure, while the *Xcv*Δ*xopS(XopS-HA)* strain was able to maintain stomatal opening (Figure 1D). These results suggest that *Xcv* suppresses stomatal closing in a T3E dependent manner and that XopS is a major effector contributing to this effect. Furthermore, transient expression of XopS in leaves of *N. benthamiana* significantly inhibited stomatal closure in response to the bacterial MAMP flg22, but did not affect the stomatal response to ABA (Supplemental Figure S2), indicating that, in these plants, stomatal closure can occur normally in response to cues other than MAMP perception.

**Figure 2:**
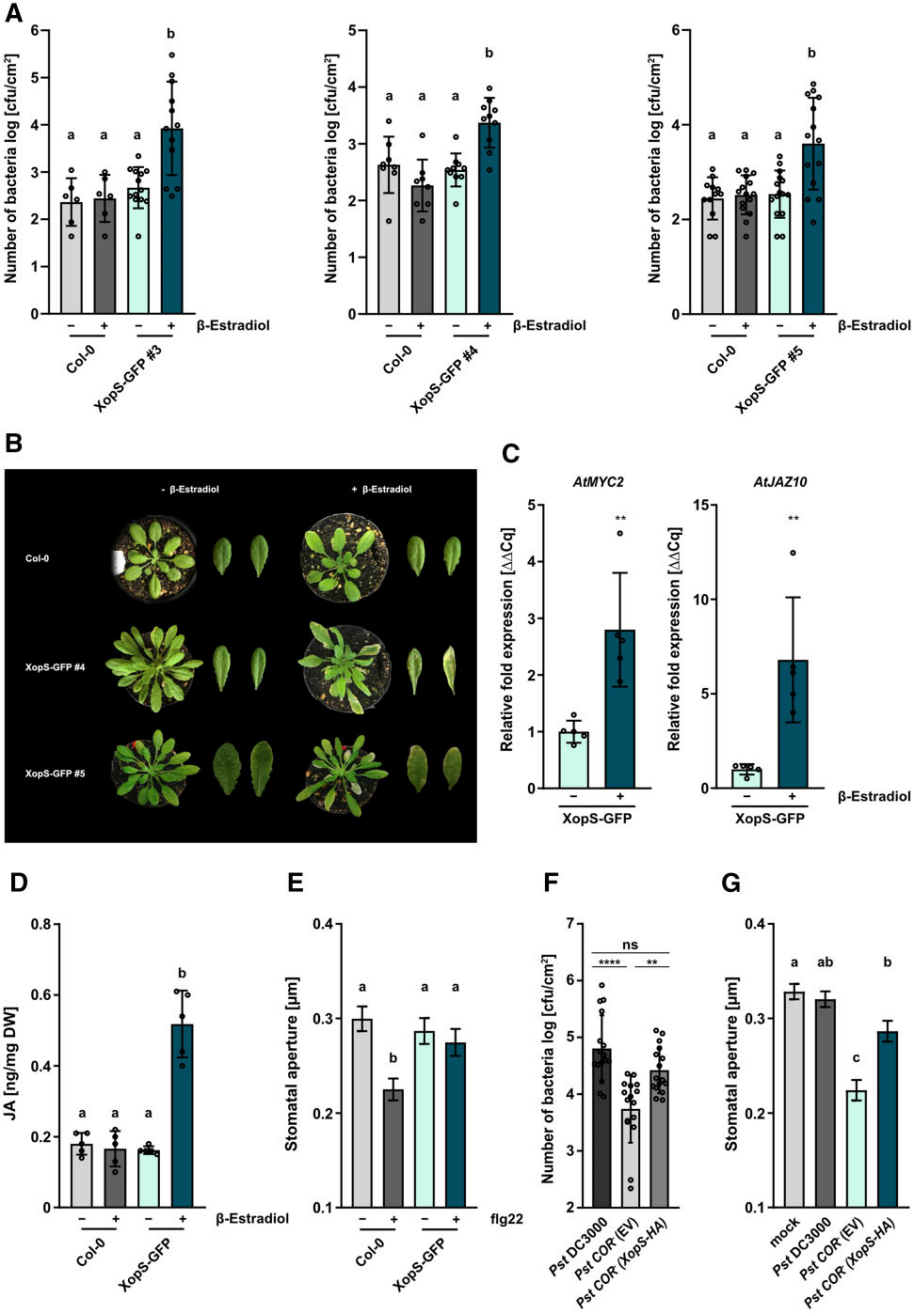
Inducible expression of XopS-GFP in transgenic Arabidopsis lines interferes with MTI and triggers JA signaling. A, Expression of XopS-GFP supports growth of *Pst* DC3000 Δ*hrcC* type-III defective bacteria. WT Col-0 and XopS-GFP transgenic Arabidopsis plants of indicated independent lines were treated with 50 µM β-estradiol and syringe-inoculated after 24 h with *Pst* DC3000 Δ*hrcC* bacteria at OD_600_ = 0.00002. CFU were determined 5 dpi. Bars represent the mean of at least *n* = 3 biological replicates and two technical replicates per biological replicate ± SD. Letters above bars represent a statistical significance determined by one-way ANOVA (*P* < 0.05). The experiment was carried out 3 times with similar results. B, Chlorotic phenotypes of indicated independent transgenic Arabidopsis lines expressing XopS-GFP under the control of the β-estradiol inducible promoter compared to the untransformed Col-0 control. Pictures were taken 5 days after spraying 50 µM β-estradiol. C, Gene expression analysis of JA marker genes in one representative XopS-GFP transgenic Arabidopsis line. Samples were taken 4 h after β-Estradiol induction. Total RNA was extracted and the mRNA levels of indicated marker genes was measured by RT-qPCR and compared to mock treated leaves. *UBIQUITIN CONJUGATING ENZYME 9* was used as a reference gene. Bars represents the mean of *n* = 5 biological replicates ± SD and asterisks (**P < 0.01) mark significant differences according to Student’s t test. The experiment was carried out twice with similar results. D, JA levels in one representative XopS-GFP transgenic Arabidopsis line 24 h after induction of protein expression by spraying 50 µM β-estradiol, compared to Col-0 plants. - indicates plants that were sprayed with the mock control. DW, Dry weight. Bars represent the mean of *n* = 5 pools of four independent plants each ±SD. Letters above bars represent the statistical significance as determined by one-way ANOVA (*P* < 0.05). E, Stomatal aperture measurement in Col-0 and transgenic Arabidopsis XopS-GFP line 5 upon induction of protein expression with 50 µM β-estradiol. Leaf discs were floated on water (control) or on water supplemented with 25 µM flg22 for 2 h prior to the measurement of stomatal aperture under a microscope. Approximately 100 apertures from *n* = 3 independent plants were measured per individual treatment and are represented as width/length ratio. Bars represent the mean ± se. Letters above bars represent the statistical significance determined by one-way ANOVA (*P* < 0.05). The experiment was carried out twice with similar results. F, Translocation of XopS-HA supports growth of *COR^−^Pst* DC3000 bacteria. Arabidopsis Col-0 plants were surface-inoculated with *Pst* DC3000, *Pst* DC3000 *COR*^−^ complemented with EV or the complementation strain *Pst* DC3000 *COR*^−^ (*XopS-HA*) at OD_600_ = 0.2. CFU were determined 5 dpi. Bars represent the mean of *n* = 8 biological replicates (and two technical replicates per biological replicate) ±sd. Significant differences are marked by asterisks (****P < 0.0001; **P < 0.01) according to one-way ANOVA. The experiment was carried out 3 times with similar results. G, Stomatal aperture measurement in Arabidopsis Col-0 leaf discs floated on water (mock control), *Pst* DC3000, *Pst* DC3000 *COR*^−^ complemented with EV or the complementation strain *Pst* DC3000 *CO^-^*^−^ (*XopS-HA*) at OD_600_ = 0.2. The measurement of stomatal aperture under a microscope was performed 3 h posttreatment. Approximately 100 apertures from *n* = 4 different plants were measured per individual treatment and are represented as width/length ratio. Bars represent the mean ± se and letters above bars represent statistical significance determined by one-way ANOVA (*P* < 0.05). The experiment was carried out twice with similar results.

To investigate whether XopS interferes with MTI in general, we generated transgenic Arabidopsis lines expressing a XopS-GFP fusion protein under the control of a β-estradiol inducible promoter; expression of the fusion protein was verified by immunoblotting (Supplemental Figure S3A). Independent transgenic Arabidopsis lines supported enhanced bacterial growth of a nonpathogenic *ΔhrcC P.* *syringae* DC3000 (*Pst* DC3000) strain upon induction of XopS-GFP expression (Figure 2A). This indicates that XopS-GFP interferes with postinvasive MTI responses in Arabidopsis.

Interestingly, the two transgenic lines with the highest XopS-GFP expression levels (lines 4 and 5) developed chlorotic symptoms ∼5 days after β-estradiol treatment, independent of bacterial inoculation (Figure 2B). This phenotype is reminiscent of T3E expressing plants with an activated JA response (Gimenez-Ibanez et al., 2014). Thus, we analyzed expression of JA marker genes induced early in the JA response in a representative transgenic line. Expression of the JA marker genes *AtMYC2* and *AtJAZ10* was greatly induced in XopS-GFP plants 4 h after β-estradiol treatment (Figure 2C). This is in line with elevated JA levels in XopS-GFP expressing Arabidopsis plants 24 h after induction with β-estradiol (Figure 2D).

In accordance with our previous observations, inducible expression of XopS-GFP in Arabidopsis prevented stomatal closure in response to treatment with flg22 (Figure 2E). Based on these observations, we hypothesized that XopS functions similarly to COR in promoting bacterial susceptibility by inducing JA responses, as demonstrated by COR-deficient (*COR*^−^) *Pst* DC3000, which is less virulent when surface inoculated on Arabidopsis plants (Melotto et al., 2006). To investigate whether XopS could restore the reduced virulence of a *COR*^−^  *Pst* DC3000 mutant strain, we surface-inoculated Arabidopsis plants with a *Pst* DC3000 *COR*^−^ strain engineered to translocate XopS. Expression of XopS-HA in the *Pst* DC3000 *COR*^−^  *(XopS-HA)* strain was confirmed by immunoblotting (Supplemental Figure S3B). The growth of *Pst* DC3000 *COR*^−^  *(XopS-HA)* bacteria was increased by ∼1 log (CFU/cm^2^) when compared to that of the same strain containing an empty vector (EV) construct (Figure 2F). In addition, incubation of Arabidopsis leaves with the XopS expressing *Pst* DC3000 *COR*^−^ bacteria resulted in a significantly reduced stomatal closure relative to that in leaves incubated with the EV control strain (Figure 2G). These data are consistent with a certain degree of functional redundancy between XopS and the phytotoxin COR.

Taken together, our experiments suggest that XopS is a major virulence factor of *Xcv* to overcome stomatal immunity. Furthermore, it interferes with postinvasive MTI and triggers a JA-response when ectopically expressed in Arabidopsis.

### *XopS* interacts with WRKY40 in yeast

We next aimed to identify the cellular targets of XopS in the host plant cell. To this end, we screened a yeast two-hybrid (Y2H) cDNA library from tobacco (*Nicotiana tabacum*) for proteins that interact with XopS. Although tobacco is not a natural host plant for *Xcv*, our previous research suggests that the conservation of potential *Xcv* T3E target proteins between tobacco and pepper is sufficiently high to identify bona fide XopS host targets (Üstün et al., 2013). A screen of >1 × 10^6^ CFU yielded four candidate XopS interaction partners (Supplemental Table S1). One candidate was identified 2 times, and the inserted cDNA encoded the predicted full-length polypeptide annotated as probable WRKY40 TF (GenBank acc. no. XM_016624265). When compared to the WRKY family of Arabidopsis using BLAST, the probable *N. tabacum* WRKY40 showed highest similarity (65%) to Arabidopsis WRKY40 (Supplemental Figure S4). Thus, we termed it *Nt*WRKY40.

**Figure 3:**
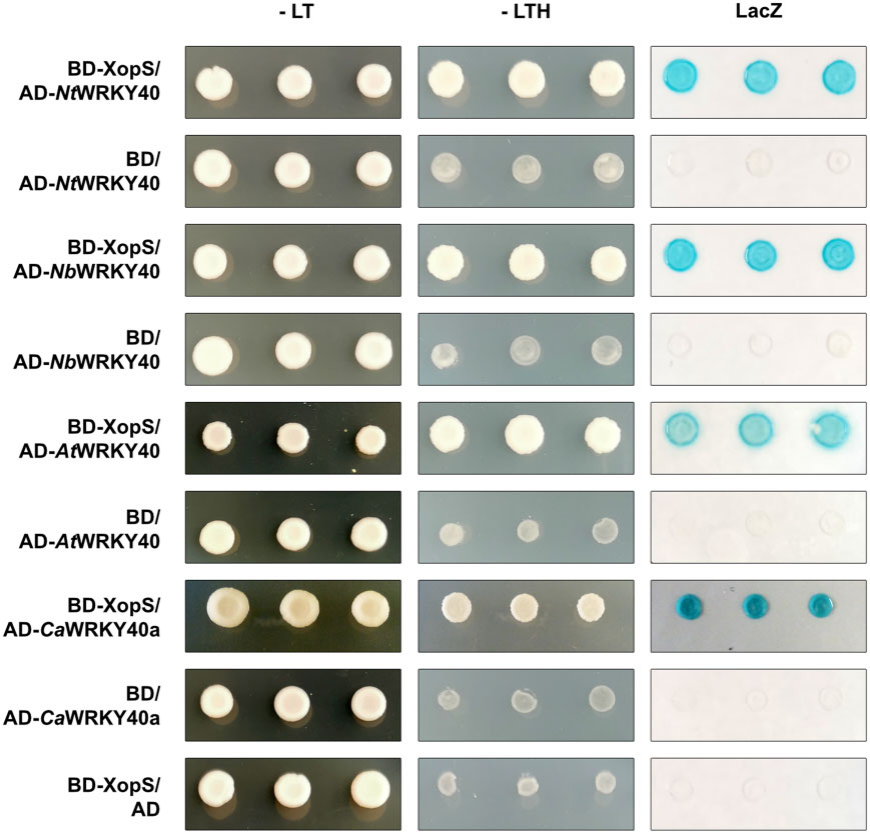
XopS interacts with WRKY40 from different plants in yeast. XopS fused to the GAL4 DNA-BD was expressed in combination with the WRKY40 protein from different plant species fused to the GAL4 AD in yeast strain Y190. Three independent transformants were grown on selective media before a LacZ filter assay was performed. Empty pGAD424 (AD) or pGBT9 (BD) vector served as negative control. *Nt*WRKY40, *N. tabacum* WRKY40; *Nb*WRKY40, *N. benthamiana* WRKY40; *At*WRKY40, *A. thaliana* WRKY40; *Ca*WRKY40a, *C. annuum* WRKY40a. –LT, yeast growth on medium without Leu and Trp; –LTH, yeast growth on medium lacking Leu, Trp, and His, indicating expression of the *HIS3* reporter gene. LacZ indicates activity of the *lacZ* reporter gene. The experiment was carried out at least 3 times with similar results.

To better understand the interaction of XopS with WRKY40, we tested the ability of the T3E to bind WKRY40 orthologs from other species. A search of the *N. benthamiana* genome at the Sol Genomics Network website (www.solgenomics.net) in combination with polymerase chain reaction (PCR) cloning identified an *Nb*WRKY40 ortholog (Niben101Scf06091g04005.1) with 100% identity to the *Nt*WRKY40 protein identified in the Y2H screen (Supplemental Figure S4). *Nicotiana* *benthamiana* also encodes two additional putative WRKY40 orthologs (*Nb*WRKY40a, Niben101Ctg16115g00003.1 and *Nb*WRKY40e, and Niben101Scf04944g05002.1), albeit displaying lower sequence similarities to *Nt*WRKY40 (Supplemental Figure S4). Pepper encodes two WRKY40 orthologs (Ca00g87690 and Ca03g32070), of which Ca00g87690 has previously been described as being involved in the positive regulation of both high temperature tolerance and *Ralstonia solanacearum* resistance (Dang et al., 2013). However, the pepper WRKY ortholog with highest similarity to the XopS interacting *Nt*WRKY40 is Ca03g32070, with 82.2% similarity (Supplemental Figure S4). To avoid confusion with the pepper WRKY40 described by Dang et al. (2013), we refer to the high-similarity Ca03g32070 as *Ca*WRKY40a. The direct interaction assays in yeast revealed that in addition to *Nt*WRKY40, XopS is able to interact with WRKY40 proteins from Arabidopsis and *N. benthamiana* as well as with pepper WRKY40a (Figure 3).

Given the presence of WRKY40 orthologs with potentially overlapping functions in *N. benthamiana* and pepper, we tested the ability of these other WRKY40 family proteins to interact with XopS. *N. benthamiana* WRKY8, which has been shown to be a positive regulator of defense responses (Ishihama et al., 2011), as well as the distantly related negative regulator of defense gene expression *Ca*WRKY1 (Oh et al., 2008), were also included in the analysis. A phylogenetic tree as well as an identity matrix showing the sequence relationships among the WRKY proteins studied here are shown in Supplemental Figure S4 and Supplemental Data Set S1 (Supplemental File S1). A Y2H assay revealed that XopS did not interact with any of the additional WRKYs tested (Supplemental Figure S5).

Thus, although binding of XopS to other WRKYs not tested in this experiment cannot be excluded, XopS appears to have a high degree of specificity in interacting with distinct WRKY40 orthologs from different species. Taken together, the Y2H experiments suggest that WRKY40 is a potential XopS target protein during compatible interactions of *Xcv* with pepper.

### *XopS* interacts with WRKY40 in planta

**Figure 4:**
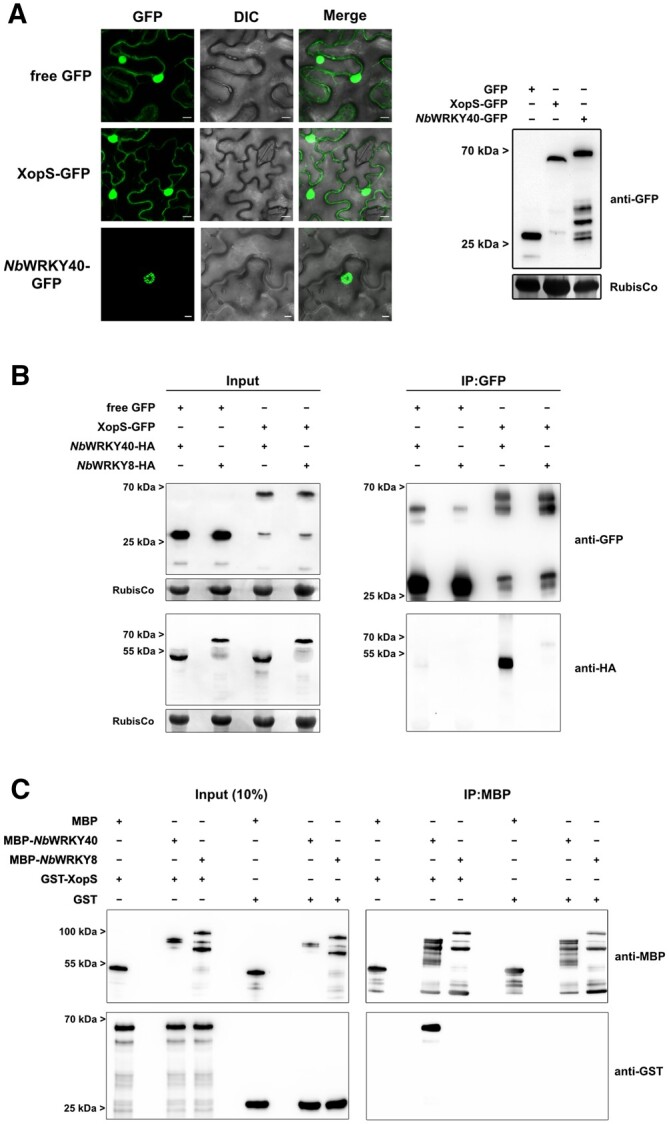
WRKY40 interacts with XopS in planta and in vitro. A, Subcellular localization of XopS-GFP and *Nb*WRKY40-GFP. GFP fusion proteins or free GFP under control of the CaMV35S promoter were expressed transiently in leaves of *N. benthamiana* using *Agrobacterium-*infiltration. The localization of transiently expressed proteins (left) was determined with confocal laser scanning microscopy 24 hpi. Scale bars represent 20 µm. DIC, Differential Interference Contrast. A representative image from 10 randomly chosen regions of interest of infiltrated leaves is shown. The experiment was carried out at least 3 times with similar results. Protein expression was verified by immunoblotting using an anti-GFP antibody (right). Amido black staining of RubisCo served as a loading control. B, Co-IP of either free GFP or XopS-GFP with *Nb*WRKY40-HA or *Nb*WRKY8-HA. Proteins were transiently co-expressed in leaves of *N. benthamiana* using *Agrobacterium*-infiltration. After 24 h, total protein extracts (Input) were subjected to IP (IP:GFP) with GFP-Trap beads, followed by immunoblotting using either anti-GFP or anti-HA antibodies. Amido black staining of RubisCo served as a loading control for input samples. The experiment was carried out at least 2 times with similar results. C, In vitro pull-down assay showing physical interaction of XopS with *Nb*WRKY40. MBP, MBP-*Nb*WRKY40, MBP-*Nb*WRKY8, GST, and GST-XopS were expressed in *E. coli.* Pull-down was performed by affinity purification of MBP-tagged proteins using amylose resin. MBP alone and GST alone were used as negative controls. Pull-down experiments with MBP-*Nb*WRKY8 were additionally performed to confirm specificity of interaction between *Nb*WRKY40 and XopS. Indicated recombinant proteins were detected before (Input 10%) and after affinity purification (IP:MBP) by immunoblotting using anti-MBP or anti-GST antibodies. The experiment was carried out twice with similar results.

If XopS interacts with WRKY40 in planta, we expect their patterns of subcellular localization to overlap. Therefore, we investigated the subcellular localization of both WRKY40 and of XopS using green fluorescent protein (GFP) fusion proteins transiently expressed in leaves of *N. benthamiana.* Expression of the fusion proteins was verified by immunoblotting (Figure 4A). While the XopS-GFP fluorescent signal showed a nucleo-cytoplasmic distribution similar to free GFP, *Nb*WRKY40-GFP fluorescence was confined to the nucleus of infiltrated cells (Figure 4A). Thus, subcellular localization of XopS and its potential target protein overlaps in the plant cell nucleus.

To determine if XopS interacts with WRKY40 in planta, a GFP pull-down assay was performed. To this end, we transiently expressed either XopS-GFP or free GFP with or without *Nb*WRKY40-HA in *N. benthamiana*. One day after infiltration, we performed pull-down of XopS-GFP using GFP-Trap beads and analyzed the eluates by immunoblotting with anti-GFP and anti-HA antibodies. XopS-GFP, but not free GFP, was able to pull down *Nb*WRKY40-HA, verifying the specific interaction of both proteins in planta (Figure 4B). In contrast, XopS-GFP was not able to pull down *Nb*WRKY8-HA, indicating that the two proteins do not interact in planta (Figure 4B).

To exclude the possibility that additional plant proteins mediate the interaction between XopS and WRKY40, we performed an in vitro pull-down assay using recombinant proteins. Recombinant maltose-binding protein (MBP) tagged *Nb*WRKY40 or *Nb*WRKY8 bound to an MBP-affinity matrix was incubated with either glutathione *S*-transferase (GST) tagged XopS or free GST. After washing, protein complex formation was analyzed by immunoblotting using anti-GST and anti-MBP antibodies. The immunoblot revealed that MBP-*Nb*WRKY40, but not MBP-*Nb*WRKY8 or free MBP, was able to pull down GST-XopS, demonstrating direct and specific physical interaction between XopS and its potential target protein without any additional eukaryotic factors (Figure 4C).

In summary, the data imply that XopS and WRKY40 specifically interact in planta through direct physical association.

### Virus-induced gene silencing of CaWRKY40a in pepper leads to increased resistance toward Xcv

**Figure 5:**
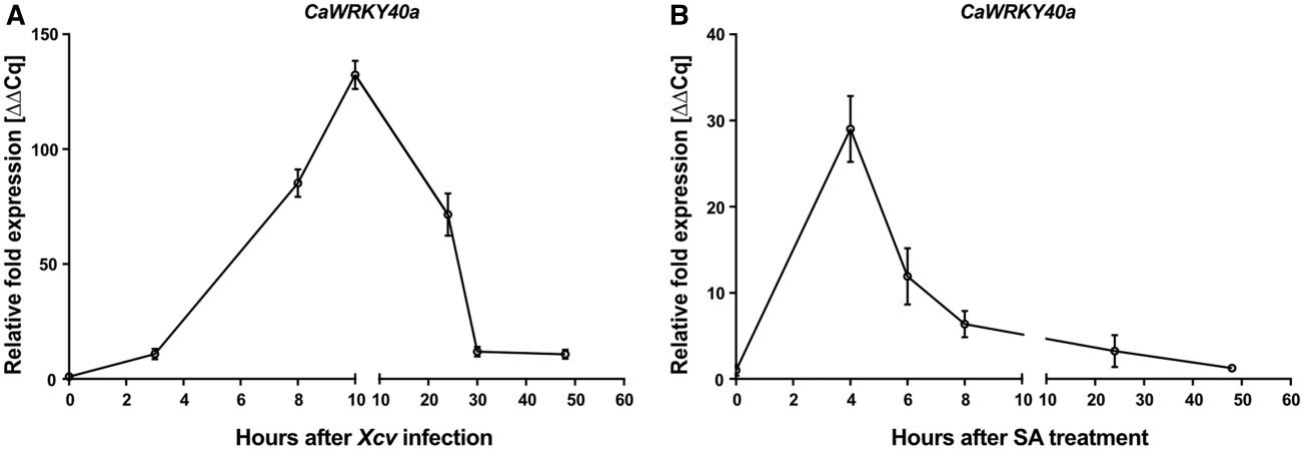
CaWRKY40 expression is induced in response to defense signals. A, Time course of *CaWRKY40a* expression in response to *Xcv* WT infection. Leaves of pepper plants were syringe-inoculated with *Xcv* WT (*Xcv*) at OD_600_ = 0.2 (0 h after *Xcv* infection) and samples were taken for the quantification of *CaWRKY40a* transcript levels at indicated time points. The respective mRNA levels were compared to the *CaWRKY40a* mRNA levels at 0 hpi. Each data point represents the mean of *n* = 5 biological replicates ± se. *Tubulin* was used as a reference gene. The experiment was carried out twice with similar results. B, Time course of *CaWRKY40a* expression in response to SA. Leaves of pepper plants were sprayed with 5 mM SA (0 h after SA treatment), samples were taken for the quantification of *CaWRKY40a* mRNA levels at indicated time points and compared to mRNA levels at 0 h after SA treatment. Each data point represents the mean of *n* = 3 biological replicates ± se. *Actin* was used as a reference gene. The experiment was carried out once.

Having established that XopS directly binds to WRKY40 in plant cells, we sought to investigate the role this TF plays in the interaction between pepper and *Xcv*. Many WRKY TFs respond transcriptionally to defense signaling molecules (Eulgem et al., 2000). Although expression of *CaWRKY40a* was readily detectable in leaves from naïve pepper plants (Supplemental Figure S6), mRNA levels rapidly increased after infection with WT *Xcv*, peaking at 10 h postinoculation (hpi) (Figure 5A). Expression then began to decrease again until reaching a low-level plateau at 30 hpi.

To investigate which signaling pathways might be involved in the rapid transient induction of *CaWRKY40a*, we monitored gene expression over a time course of 50 h after treatment of leaves with 5 mM SA. *CaWRKY40a* mRNA levels peaked at 4 h after SA application and then declined rapidly by ∼80% until 8 h posttreatment (Figure 5B). Subsequently, *CaWRKY40a* mRNA levels slowly decreased further to reach almost basal levels again at the end of the time course. Thus, SA treatment and *Xcv* infection each lead to a similarly rapid and transient increase in *CaWRKY40a* expression.

To obtain direct evidence for an involvement of *CaWRKY40a* in the defense against *Xcv*, we used virus-induced gene silencing (VIGS) with *Tobacco rattle virus* (TRV) of *WRKY40a* in pepper, followed by infection with *Xcv*. To avoid silencing any off-target genes, we employed the VIGS tool on the Sol Genomics Network website (Fernandez-Pozo et al., 2015) with default settings and using the entire *CaWRKY40a* CDS as a query. The VIGS tool suggested using a fragment covering the first 300 bp of the *CaWRKY40a* CDS to achieve high silencing specificity. A 21-mer siRNA generated from this silencing construct would have large numbers of matches with *CaWRKY40a* (CA03g32070; 942 matches), but no other genes were identified as being potential off-targets except *CaWRKY40* (CA00g87690) with a very low probability (three matches).

**Figure 6:**
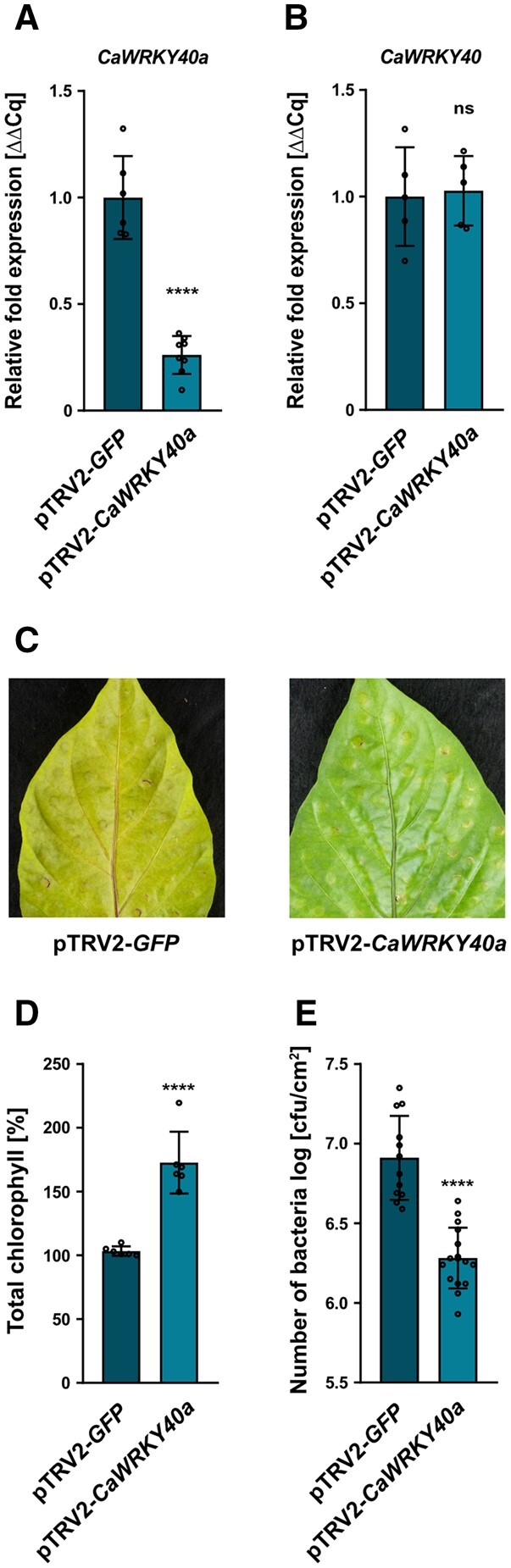
VIGS of *CaWRKY40a* in pepper enhances resistance against *Xcv* infection. A, Verification of *CaWRKY40a* downregulation in pTRV2-*CaWRKY40a* (*CaWRKY40a* silencing) compared with pTRV2-*GFP* (*GFP* silencing; control) in VIGS pepper plants. Three weeks after infiltrating pepper cotyledons with the silencing constructs, total RNA was isolated from excised leaves treated with 5 mM SA for 4 h. The mRNA level of *CaWRKY40a* in pTRV2-*CaWRK40a* was measured by RT-qPCR and compared to *CaWRKY40a* expression in pTRV2-*GFP* control plants. *Tubulin* was used as a reference gene. Bars represent the mean of at least *n* = 6 biological replicates ± sd (*n* = 6 for pTRV2-*GFP* and *n* = 8 for pTRV2-*CaWRKY40a*). Asterisks (****P < 0.0001) mark significant differences according to Student’s *t* test. B, Exclusion of potential off targets during downregulation of *CaWRKY40a* in VIGS pepper plants. Three weeks after infiltrating pepper cotyledons with the silencing constructs, total RNA was isolated from excised leaves treated with 5 mM SA for 4 h. The mRNA level of *CaWRKY40* in pTRV2-*CaWRK40a* plants was measured by RT-qPCR and compared to *CaWRKY40* expression in pTRV2-*GFP* control plants. *Tubulin* was used as a reference gene. Bars represent the mean of *n* = 5 biological replicates ± sd. ns according to Student’s *t* test. The experiment was carried out twice with similar results. C, Disease symptom development in leaves of pTRV2-*CaWRKY40a* pepper plants compared to pTRV2-*GFP* control plants, syringe-inoculated with WT *Xcv* bacteria at an OD_600_ = 0.0001. Pictures were taken at 5 dpi. A similar phenotypic difference was consistently observed in two independent experiments. D, Chlorophyll levels in leaves of pTRV2-*CaWRKY40a* pepper plants syringe-infected with *Xcv* WT relative to infected pTRV2-*GFP* plants. The percentage of total chlorophyll was determined when compared to the pTRV2-*GFP* control (set to 100%). The measurement was performed 5 dpi. Each bar represents the mean of *n* = 6 biological replicates ± SD and asterisks (****P < 0.0001) mark significant differences according to Student’s t test. The experiment was carried out twice with similar results. E, Bacterial growth of *Xcv* WT in leaves of pTRV2-*CaWRKY40a* compared to pTRV2-*GFP* control plants. Leaves were syringe-inoculated with a bacterial density of OD_600_ = 0.0001 and CFU in infected tissue were quantified at 5 dpi. Bars represent the mean of at least *n* = 6 biological replicates (and two technical replicates per biological replicate) ±sd (*n* = 6 for pTRV2-*GFP* and *n* = 8 for pTRV2-*CaWRKY40a*). Asterisks (****P < 0.0001) mark significant differences according to Student’s t test. The experiment was carried out 3 times with similar results.

After inserting the suggested *CaWRKY40a* fragment into the silencing vector pTRV2, we infiltrated the cotyledons of pepper seedlings with a mixture of *Agrobacterium tumefaciens* strains of pTRV1 (*Cauliflower mosaic virus* [CaMV]; CaMV 35S-driven TRV RNA1) and pTRV2-*CaWRKY40a* (TRV RNA2 containing the target sequence), or pTRV2-*GFP* (serving as a control for infection symptoms). To increase the signal to noise ratio, we harvested leaves 4 weeks after infiltration and treated them with 5 mM SA for 4 h. We confirmed strong downregulation of *CaWRKY40a* by reverse transcription-quantitative PCR (RT-qPCR) in plants infiltrated with the pTRV2-*CaWRKY40a* vector as compared to the pTRV2-*GFP* control (Figure 6A). In contrast, we saw no change in the expression of *CaWRKY40*, confirming the specificity of the silencing approach (Figure 6B).

Subsequently, we syringe-inoculated silenced plants with *Xcv* and monitored disease progression over the course of 5 days. While infected leaves of pTRV2-*GFP* plants developed strong chlorosis, silencing of *CaWRKY40a* substantially reduced the appearance of visible disease symptoms (Figure 6C). We measured chlorophyll content as a proxy to quantify the infection phenotype and, in accordance with the development of leaf chlorosis, found that chlorophyll content was significantly lower in control plants than in *CaWRKY40a* silenced plants (Figure 6D). Strikingly, bacterial multiplication in pTRV2-*CaWRKY40a* plants was significantly lower than in the control at 5 dpi (Figure 6E), indicating that silencing of *WRKY40a* in susceptible pepper plants leads to enhanced postinvasion resistance toward *Xcv* infection.

### WRKY40 negatively regulates defense gene expression

**Figure 7:**
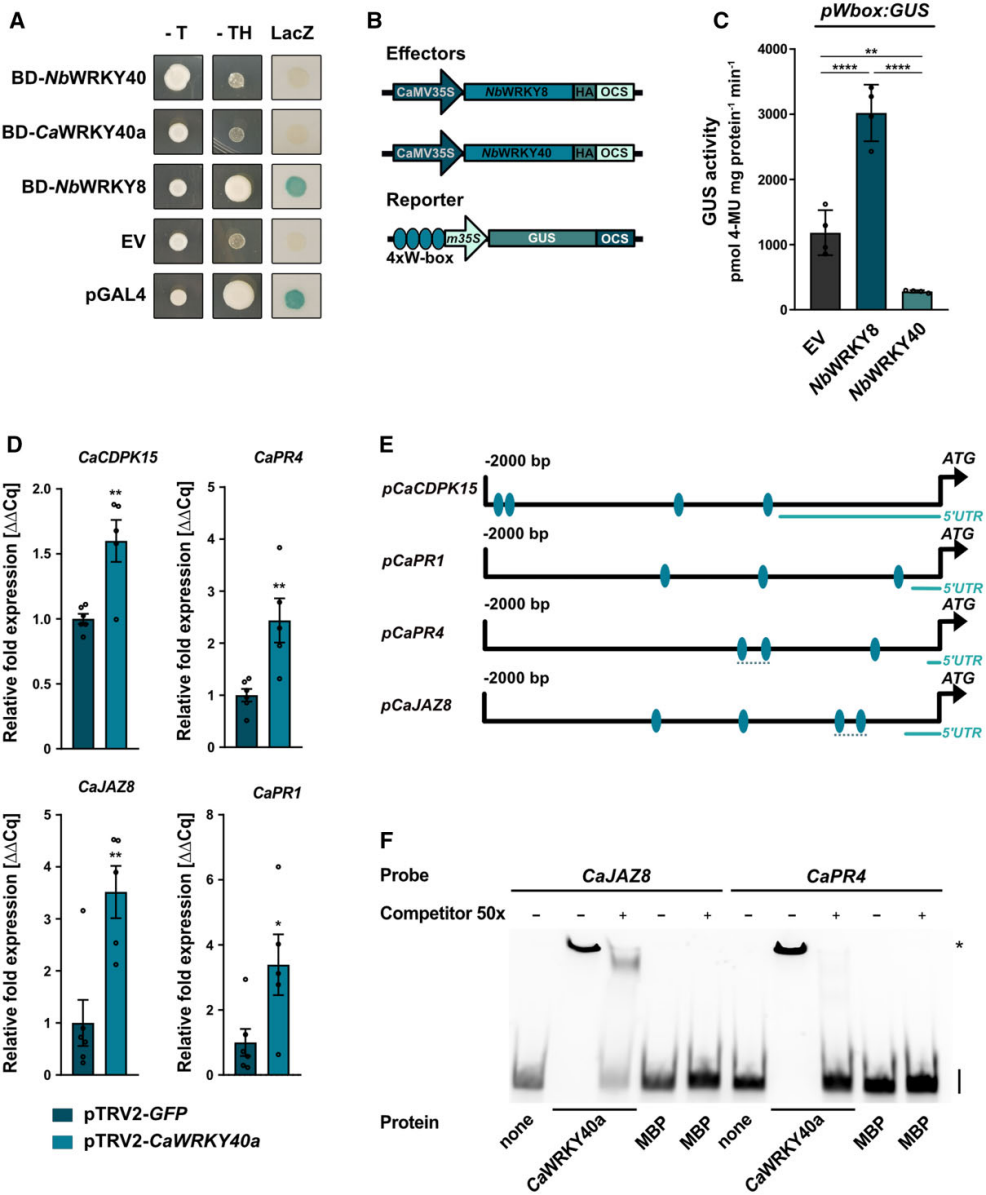
WRKY40 is a negative regulator of defense gene expression. A, Yeast transactivation assay. WRKY proteins were expressed as GAL4 DNA-BD fusion proteins in yeast strain Y190. *HIS3* and *LacZ* reporter gene activity was monitored 3 days after spotting transformed cells on selective medium. The pGBT9 EV served as negative control and pGAL4 vector (pGAL4) expressing the full length GAL4 protein was used as positive control. -T and -TH, indicating expression of the *HIS3* reporter gene. LacZ indicates activity of the *lacZ* reporter gene. *Nb*WRKY40, *N. benthamiana* WRKY40; *Ca*WRKY40a, *C. annuum* WRKY40a; *Nb*WRKY8, *N. benthamiana* WRKY8. The experiment was carried out three times with similar results. B, Schematic representation of the effector and reporter constructs used in the transient expression assay of W-box driven promoter activity. Effector constructs contained either *Nb*WRKY8 or *Nb*WRKY40 under control of the constitutive CaMV35S promoter. A C-terminal HA-tag enabled immune detection of the protein. The *GUS* reporter gene was submitted to the control by a minimal CaMV35S promoter (*m35S*) preceded by an oligonucleotide containing four W-boxes. OCS, terminator of the octopine synthase. C, W-box driven GUS reporter gene activity in *N. benthamiana*. The GUS reporter gene was submitted to the control of a chimeric promoter consisting of a 4× W-box element fused to the minimal CaMV35S promoter. The reporter construct was transiently co-expressed with EV HA, *Nb*WRKY8-HA or *Nb*WRKY40-HA as effector constructs. GUS activity was determined 48 h post-*Agrobacterium*-infiltration and is represented as pmol 4-Methylumbelliferone mg protein^−1^ min^−1^. Bars represent the mean of *n* = 4 biological replicates ± SD and asterisks (**P < 0.01; ****P < 0.0001) mark significant differences according to one-way ANOVA. The experiment was carried out 3 times with similar results. D, Defense gene expression in pTRV2-*CaWRKY40a* pepper plants upon infection with *Xcv*. Leaves of pTRV2-*CaWRKY40a* and pTRV2-*GFP* control pepper plants were syringe-inoculated with *Xcv* WT bacteria at OD_600_ = 0.2 and samples were taken 10 hpi. The mRNA levels of four indicated potential *Ca*WRKY40a target genes were measured by RT-qPCR in pTRV2-*CaWRKY40a* plants and were compared to their mRNA levels in pTRV2 control plants. *Ubiquitin-conjugating protein* (*UBI*-3) was used as a reference gene. Bars represent the mean of at least *n* = 5 biological replicates ± SE (*n* = 6 for pTRV2-*GFP* and *n* = 5 for pTRV2-*CaWRKY40a*). Asterisks (*P < 0.05; **P < 0.01) mark significant differences according to Student’s t test. The experiment was carried out twice with similar results. E, Schematic representation of the 2 kb promoter regions upstream of the translational start site of indicated candidate *Ca*WRKY40a target genes. Ellipses indicate the position of predicted W-boxes. Dashed lines represent position of the probes used for the EMSA. F, EMSA shows binding of *Ca*WRKY40a to W-boxes contained in the *CaJAZ8* and *CaPR4* promoter regions. MBP-*Ca*WRKY40a was produced in *E. coli* and incubated with Cy5-labeled 150 bp DNA fragments derived from the *CaJAZ8* and *CaPR4* promoter regions. Each DNA fragment contained two predicted W-boxes. Protein–DNA complexes were separated from unbound probe on a 5% TBE gel. Unlabeled DNA fragment was used as a competitor with a 50-fold (50×) excess over the amount of used probe. MBP protein alone was included as additional negative control. On the right-hand side of the gel, specific retarded protein–DNA complexes are marked by an asterisk, whereas free running probes are designated by a black bar. The experiment was carried out three times with similar results.

The enhanced resistance of pTRV2-*CaWRKY40a* plants to infection by *Xcv* suggests that, similar to the scenario in Arabidopsis, the TF *Ca*WRKY40a is involved in negative regulation of defense gene expression. To investigate whether XopS-interacting WRKY40s are capable of transactivation, we performed a yeast transactivation assay (Ye et al., 2004). To this end, we cloned the full-length open reading frames of *NbWRKY40* and *CaWRKY40a* into the pGBT9 vector to create fusion proteins with the GAL4 DNA-binding domain (BD). *Nb*WRKY8, a known positive regulator of gene expression (Ishihama et al., 2011), served as a control. We separately transformed the vectors into the Y190 yeast reporter strain and assayed the transformants for growth on histidine lacking medium and for *LacZ* activity. While BD-*Nb*WRKY8 was able to induce strong reporter gene expression, BD-*Nb*WRKY40 and BD-*Ca*WRKY40a did not display transactivation activity in yeast (Figure 7A).

To confirm the repressor activity of WRKY40 in planta, we fused a β-Glucuronidase (GUS) reporter gene to a nucleotide sequence containing four W-boxes in front of a minimal CaMV35S promoter (Figure 7B). Co-expression of the *W-box:GUS* construct with CaMV35S driven *NbWRKY8* resulted in significant induction of GUS activity relative to the EV control (Figure 7C), which is in line with a role of WRKY8 as a positive regulator of gene expression (Ishihama et al., 2011). In contrast, co-expression with CaMV35S-driven *WRKY40* significantly repressed GUS activity below the level of the EV control, suggesting that WRKY40 can suppress basal GUS expression mediated by the minimal CaMV35S promoter (Figure 7C). Expression of both WRKY proteins tested was verified by immunoblotting (Supplemental Figure S7). Thus, WRKY40 likely functions as a negative regulator of gene expression.

We next analyzed pathogen-induced defense gene expression in pTRV2-*CaWRKY40a* plants compared with control plants. Since target genes of *Ca*WRKY40a in pepper are not known, we selected genes that were described earlier to be under the regulation of WRKY TFs, such as *CaCDPK15* (Shen et al., 2016) and *CaPR4* (Huh et al., 2015), or were considered bona fide WRKY target genes based on their regulation in Arabidopsis, such as *JAZ8* and *PR1* (Pandey et al., 2010; Birkenbihl et al., 2017). As a prerequisite for WRKY binding, we confirmed that all selected genes contain W-boxes within the 2 kb upstream of their transcriptional start site (Figure 7E). When measured 10 h after syringe infection with WT *Xcv* bacteria, the expression of all genes tested was significantly higher in pTRV2-*CaWRKY40a* plants than in those infected with the pTRV2-*GFP* silencing control (Figure 7D), suggesting that silencing of *CaWRK40a* leads to a faster and/or stronger defense response on the transcriptional level that correlates with enhanced *Xcv* resistance. We verified the effective silencing of *CaWRKY40a* in the plants under scrutiny by RT-qPCR (Supplemental Figure S8).

We next sought to investigate whether the increased expression level of genes was due to the loss of *Ca*WRKY40a binding to their promoter regions. To this end, we analyzed the direct binding of recombinant MBP-*Ca*WRKY40a to an ∼150 bp *CaJAZ8* or *CaPR4* promoter fragment comprising two predicted W-boxes (Figure 6E and Supplemental Figure S4B) using an electrophoretic mobility shift assay (EMSA).

The results indicate that the MBP-*Ca*WRKY40a protein was able to bind to CY5-labeled promoter fragments containing the W-boxes, resulting in a shift of mobility (Figure 7F). Excess unlabeled probe (the same DNA fragment without CY5) efficiently competed for binding to the protein, indicating specificity of binding. No binding signal was detected in reactions without added protein (lanes 1 and 6) or with MBP alone (lanes 5 and 10). Mutation of the W-boxes within EMSA probes abolished binding to MBP-*Ca*WRKY40a (Supplemental Figure S9 and Supplemental Data Set S2 (Supplemental File S1)). Thus, *CaJAZ8* and *CaPR4* are likely subject to direct regulation by WRKY40a in pepper plants.

### The ability of XopS to interfere with stomatal immunity requires WRKY40

**Figure 8:**
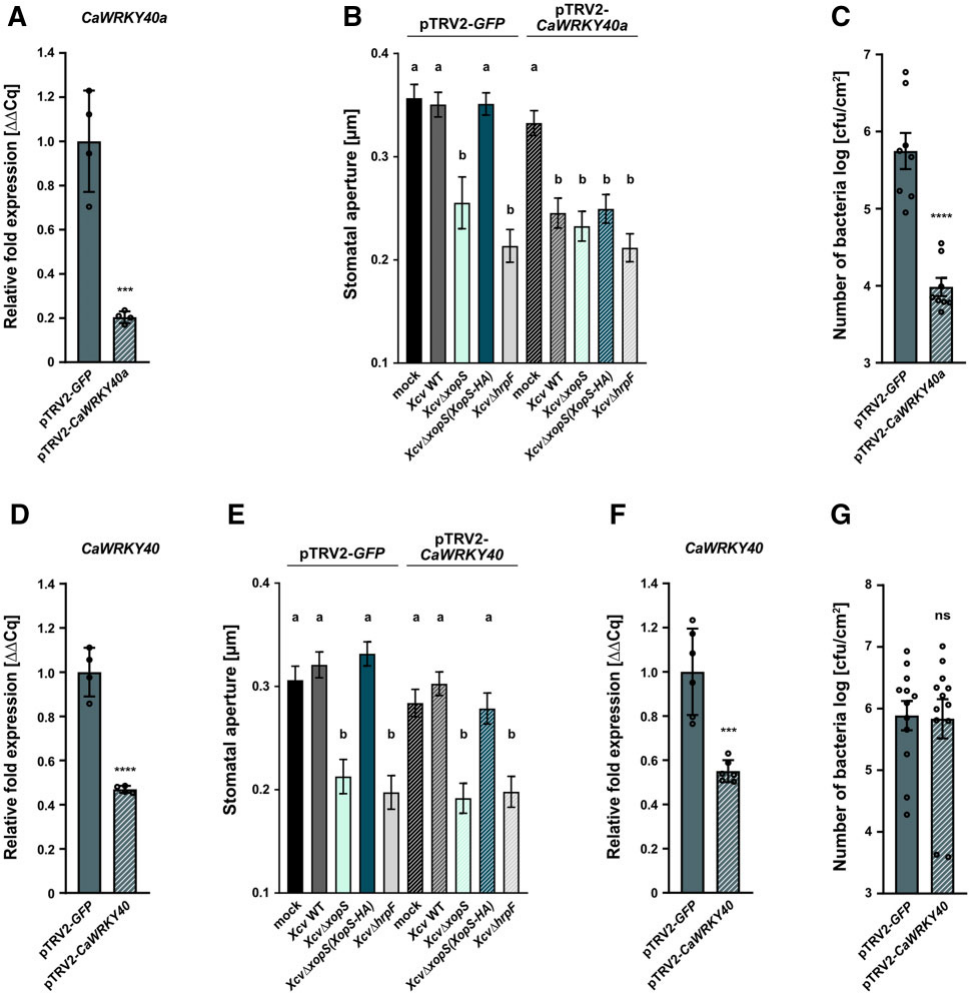
The ability of XopS to interfere with stomatal immunity in pepper requires WRKY40. A, Verification of *CaWRKY40a* downregulation in VIGS pepper pTRV2-*CaWRKY40a* plants. Three weeks after infiltrating pepper cotyledons with the silencing constructs, total RNA was isolated from excised leaves treated with 5 mM SA for 4 h. The mRNA level of *CaWRKY40a* in pTRV2-*CaWRK40a* was measured by RT-qPCR and compared with *CaWRKY40a* expression in pTRV2-*GFP* control plants. *Tubulin* was used as a reference gene. Bars represent the mean of *n* = 4 biological replicates ± sd. Asterisks (***P < 0.001) mark significant differences according to Student’s t test. B, Stomatal apertures from pTRV2-*GFP* and pTRV2-*CaWRKY40a* VIGS pepper plants. Leaf discs were floated on water (mock) or on water containing *Xcv* WT, *XcvΔxopS*, *XcvΔxopS(XopS-HA) or XcvΔhrpF* strains at an OD_600_ = 0.2. The measurement of stomatal aperture was performed 2 h posttreatment. Approximately 100 apertures from *n* = 4 different plants were measured per individual treatment and are represented as width/length ratio. Bars represent the mean ± SE and letters above the bars represent a statistical significance determined by one-way ANOVA (*P* < 0.05). The experiment was carried out twice with similar results. C, Bacterial growth of surface-inoculated *Xcv* WT in leaves of pTRV2-*CaWRKY40a* compared with pTRV2-*GFP* control plants. Leaves were dip-inoculated with bacteria at a density of OD_600_ = 0.2 and CFU in infected tissue were quantified at 6 dpi. Bars represent the mean of *n* = 4 biological replicates (and two technical replicates per biological replicate) ± sd. Asterisks (****P < 0.0001) mark significant differences according to Student’s *t* test. The experiment was carried out twice with similar results. D, Verification of *CaWRKY40* downregulation in VIGS pepper pTRV2-*CaWRKY40* plants. Three weeks after infiltrating pepper cotyledons with the silencing constructs, total RNA was isolated from excised leaves treated with 5 mM SA for 4 h. The mRNA level of *CaWRKY40a* in pTRV2-*CaWRK40* was measured by RT-qPCR and compared with *CaWRKY40* expression in pTRV2-*GFP* control plants. *Tubulin* was used as a reference gene. Bars represent the mean of *n* = 4 biological replicates ± sd. Asterisks (****P < 0.0001) mark significant differences according to Student’s *t* test. E, Stomatal apertures of pTRV2-*GFP* and pTRV2-*CaWRKY40* VIGS pepper plants. Leaf discs were floated on water (mock) or on water containing *Xcv* WT, *XcvΔxopS*, *XcvΔxopS(XopS-HA)* or *XcvΔhrpF* strains at an OD_600_ = 0.2. The measurement of stomatal aperture was performed 2 h posttreatment. Approximately 100 apertures from *n* = 4 different plants were measured per individual treatment and are represented as width/length ratio. Bars represent the mean ± se and letters above bars represent the statistical significance determined by one-way ANOVA (*P* < 0.05). The experiment was carried out twice with similar results. F, Verification of *CaWRKY40* downregulation in VIGS pepper pTRV2-*CaWRKY40* plants. Three weeks after infiltrating pepper cotyledons with the silencing constructs, total RNA was isolated from excised leaves treated with 5 mM SA for 4 h. The mRNA level of *CaWRKY40a* in pTRV2-*CaWRK40* was measured by RT-qPCR and compared to *CaWRKY40* expression in pTRV2-*GFP* control plants. *Tubulin* was used as a reference gene. Bars represent the mean of *n* = 6 biological replicates ± sd. Asterisks (***P < 0.001) mark significant differences according to Student’s *t* test. G, Bacterial growth of surface-inoculated *Xcv* WT in leaves of pTRV2-*CaWRKY40* compared to pTRV2-*GFP* control plants. Leaves were dip-inoculated with a bacterial density of OD_600_ = 0.2 and CFU in infected tissue were quantified at 6 dpi. Bars represent the mean of *n* = 6 biological replicates (and two technical replicates per biological replicate) ±sd. ns according to Student’s *t* test. The experiment was carried out twice with similar results.

We next tested whether *Ca*WRKY40a is involved in XopS-induced interference with stomatal immunity. Incubation of pepper leaves from *CaWRKY40a* silenced plants with different *Xcv* bacterial strains for 2 h led to a significant reduction of stomatal aperture (Figure 8, A and B), indicating that in the absence of *CaWRKY40a, Xcv-*induced stomatal movement is not affected even in the presence of XopS. As observed before, the ability to prevent stomatal closure in pTRV2-*GFP* control plants was strictly dependent on the translocation of XopS (Figure 8B). We observed an approximately 2 log reduction of bacterial multiplication in *CaWRKY40a* silenced plants after dip-inoculation with WT *Xcv* (Figure 8C), suggesting that *CaWRKY40a* silencing strongly increases resistance to surface inoculated *Xcv* bacteria. In contrast, VIGS of *CaWRKY40* (Dang et al., 2013) did not affect either the ability of *Xcv* to interfere with stomatal closure or bacterial multiplication upon surface inoculation with *Xcv* (Figure 8, D–G).

**Figure 9:**
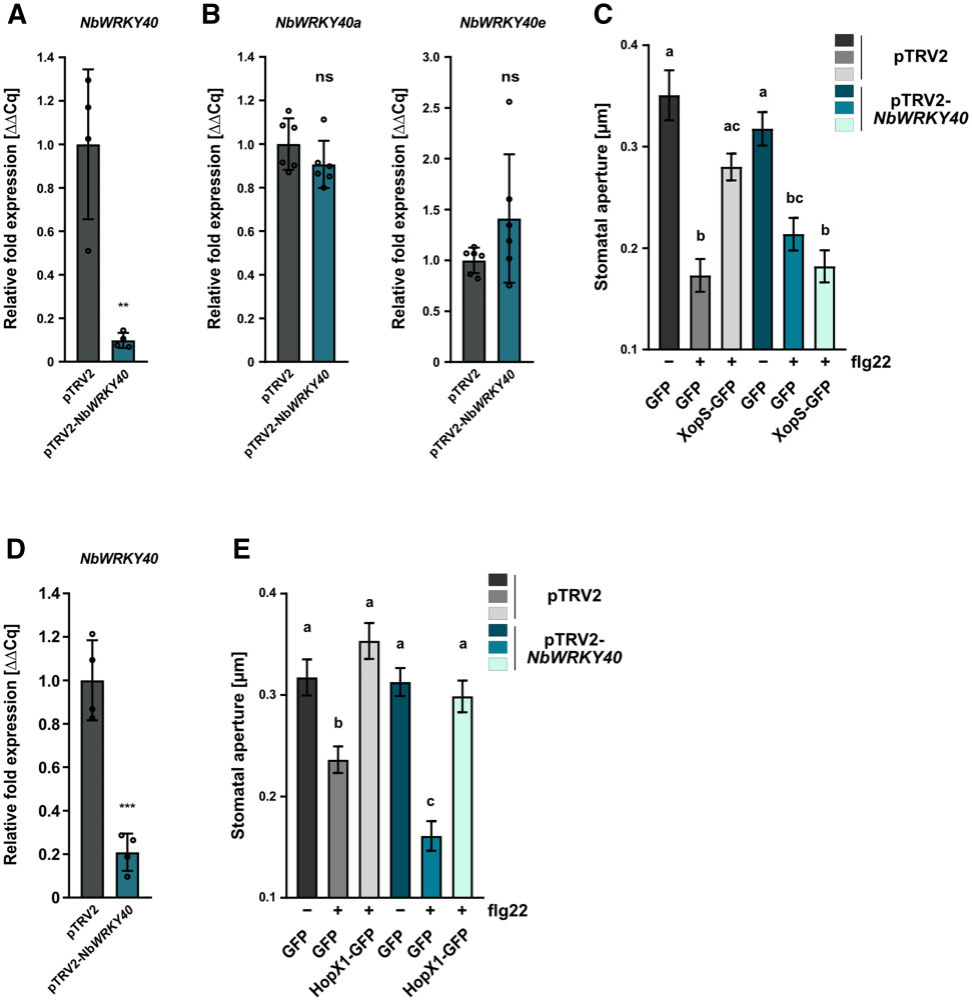
VIGS of *WRKY40* in *N. benthamiana* affects stomatal closure in response to a MAMP stimulus. A, Verification of *NbWRKY40* downregulation in pTRV2-*NbWRKY40* (*NbWRKY40* silencing) compared with pTRV2 (EV, silencing control) in VIGS *N. benthamiana* plants. Two weeks after infiltrating *N. benthamiana* plants with the silencing constructs, total RNA was isolated from excised leaves treated with 5 mM SA for 4 h. The mRNA level of *NbWRKY40* in pTRV2-*NbWRKY40* was measured by RT-qPCR and compared to *NbWRKY40* expression in pTRV2 control plants. *Actin* was used as a reference gene. Bars represent the mean of *n* = 4 biological replicates ± sd and asterisks (**P < 0.01) mark significant differences according to Student’s *t* test. B, Exclusion of potential off target effects during downregulation of *NbWRKY40* in VIGS *N. benthamiana* plants. The mRNA levels of *NbWRKY40a* and *NbWRKY40e* in pTRV2-*NbWRKY40* plants were measured by RT-qPCR and compared with *NbWRKY40a* and *NbWRKY40e* expression in pTRV2 control plants. *Actin* was used as a reference gene. Bars represent the mean of *n* = 5 biological replicates± sd. ns according to Student’s *t* test. The experiment was carried out twice with similar results. C, Stomatal aperture measurement in pTRV2-*NbWRKY40* and pTRV2 VIGS *N. benthamiana* plants transiently expressing either GFP alone or XopS-GFP. Leaf discs were floated on water (control, -) or on water supplemented with 25 µM flg22 (+) for 2 h prior to the measurement of stomatal aperture under a microscope. Approximately 100 apertures from *n* = 4 independent plants were measured per individual treatment and are represented as width/length ratio. Bars represent the mean ± se. Letters above bars represent the statistical significance determined by one-way ANOVA (*P* < 0.05). The experiment was carried out twice with similar results. D, Verification of *NbWRKY40* downregulation in pTRV2-*NbWRKY40* VIGS *N. benthamiana* plants. Two weeks after infiltrating *N. benthamiana* plants with the silencing constructs, total RNA was isolated from excised leaves treated with 5mM SA for 4 h. The mRNA level of *NbWRKY40* in pTRV2-*NbWRKY40* was measured by RT-qPCR and compared to *NbWRKY40* expression in pTRV2 control plants. *Actin* was used as a reference gene. Bars represent the mean of *n* = 4 biological replicates ± SD and asterisks (***P < 0.001) mark significant differences according to Student’s t test. E, Stomatal aperture measurement in pTRV2-*NbWRKY40* and pTRV2 VIGS *N. benthamiana* plants transiently expressing either GFP alone or HopX1-GFP. Leaf discs were floated on water (control, -) or on water supplemented with 25 µM flg22 (+) for 2 h prior to the measurement of stomatal aperture under a microscope. Approximately 100 apertures from *n* = 4 independent plants were measured per individual treatment and are represented as width/length ratio. Bars represent the mean ± se. Letters above bars represent the statistical significance determined by one-way ANOVA (*P* < 0.05). The experiment was carried out twice with similar results.

To further corroborate these findings, we next investigated whether the ability of XopS to prevent stomatal closure in response to a MAMP stimulus in *N. benthamiana* is also dependent on WRKY40. Expression of WRKY40 was significantly induced in *N. benthamiana* leaves 2 h after treatment with flg22 (Supplemental Figure S10), validating the expression of the TF during the experimental treatment. We used the VIGS tool at the Sol Genomics network website to select a 300-bp fragment of the *NbWRKY40* CDS for amplification and insertion into the pTRV2 silencing vector. VIGS resulted in a reduction of *WRKY40* gene expression by around 90% as compared to the pTRV2 control (Figure 9A). To investigate whether off-target silencing of other WRKY40 orthologs occurred in the VIGS plants, we examined the transcript levels of *NbWRKY40a* and *NbWRKY40e* and found that they were unaffected (Figure 9B).

Subsequently, we expressed either XopS-GFP or GFP alone in leaves of *WRKY40* as well as EV silenced plants using *Agrobacterium* infiltration and detected their presence by immunoblotting (Supplemental Figure S11A). We also monitored 24 hpi leaf discs of XopS-GFP expressing plants along with GFP controls for the ability to close their stomata in response to flg22. In contrast to XopS-GFP expressing leaves, leaves expressing free GFP displayed a significantly reduced stomatal aperture 2 h after treatment. This confirmed the ability of XopS to attenuate stomatal closure in response to a MAMP stimulus (Figure 9C). In response to flg22 exposure, pTRV2*-NbWRKY40* plants showed comparable stomatal closure to control plants. Expression of XopS-GFP had no significant effect on stomatal aperture in plants with reduced *WRKY40* expression (Figure 9C). These data clearly demonstrate a direct relationship between WRKY40 and the ability of XopS to interfere with stomatal immunity.

HopX1 from *P.* *syringae* pv. *tabaci* (*Pta*) 11,528 influences stomatal immunity through the degradation of JAZ proteins (Gimenez-Ibanez et al., 2014). Similar to the effect in pTRV2 control plants, transient expression of HopX1 in *WRKY40* silenced *N. benthamiana* plants (Supplemental Figure S11B) still prevented stomatal closing in response to flg22 treatment (Figure 9, D and E). This is in accordance with a model whereby HopX1 acts on a target downstream of WRKY40 in the signaling pathway leading to MAMP-induced stomatal closure.

### XopS interferes with proteasomal turnover of WRKY40 in planta

**Figure 10:**
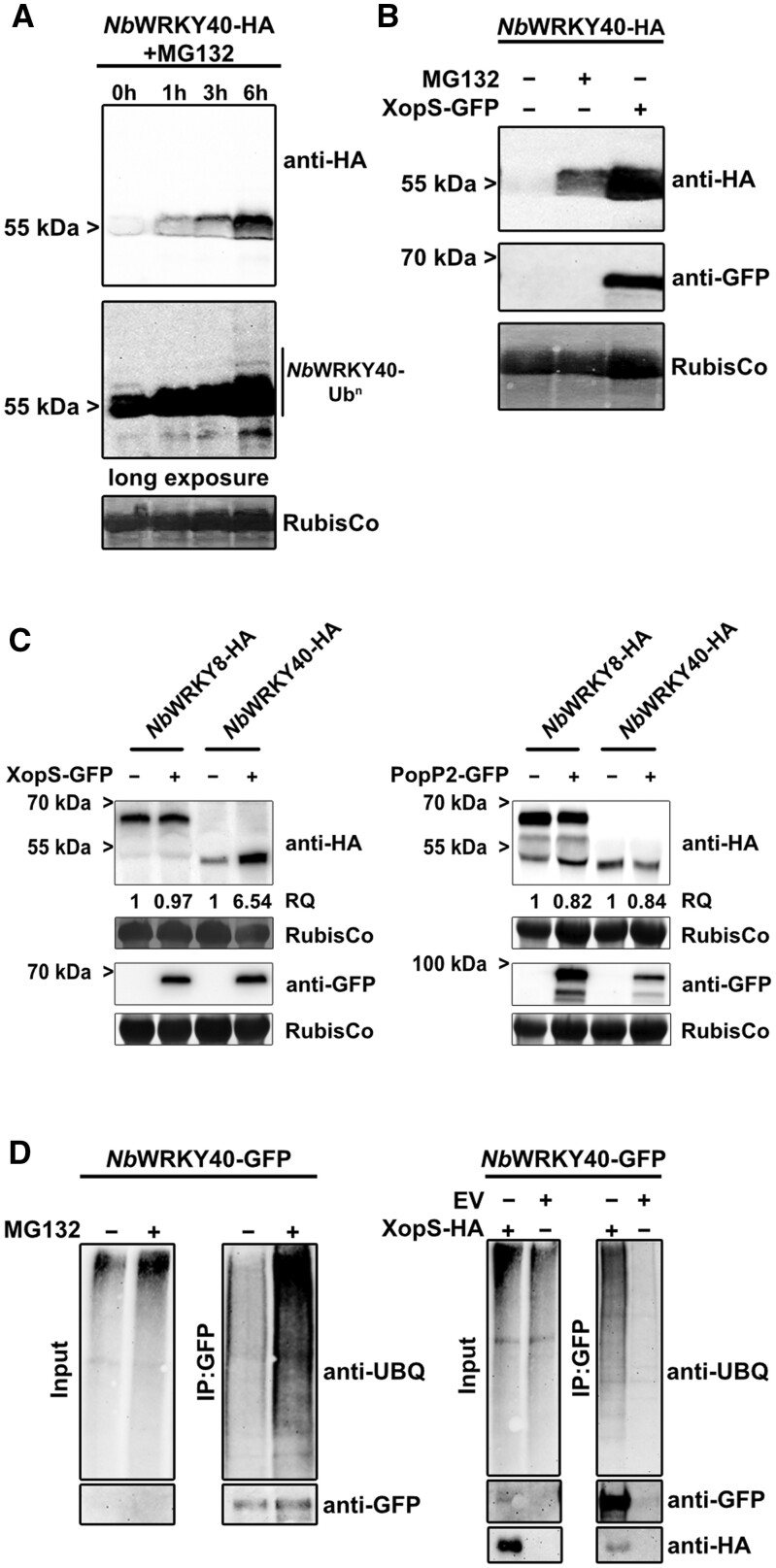
XopS protects WRKY40 from degradation. A, Inhibition of the 26S proteasome results in the accumulation of WRKY40. *Nb*WRKY40-HA was transiently expressed in leaves of *N. benthamiana* using *Agrobacterium*-infiltration. About 48 hpi leaves were treated with 200 µM MG132 and samples were taken at the time points indicated. *Nb*WRKY40-HA protein levels were monitored over time using an anti-HA antibody (upper panel: short exposure; lower panel: long exposure). Amido black staining of RubisCo served as a loading control. The experiment was carried out at least 2 times with similar results. B, Co-expression with XopS-GFP results in increased *Nb*WRKY40-HA protein levels. *Nb*WRKY40-HA was transiently expressed either alone or along with XopS-GFP or was treated with 200 µM MG132 for 6 h where indicated. About 48 hpi total protein extracts were generated and proteins were detected by immunoblotting using either anti-HA or anti-GFP antibodies. Amido black staining of RubisCo served as loading control. The experiment was carried out at least two times with similar results. C, XopS-GFP stabilizes *Nb*WRKY40-HA with a certain degree of specificity. *Nb*WRKY40-HA and *Nb*WRKY8-HA fusion proteins were transiently co-expressed in *N. benthamiana* leaves with either XopS-GFP (left panel) or PopP2-GFP (right panel) using *Agrobacterium*-infiltration. Total protein extracts were generated from infiltrated leaf material 24 hpi and proteins were detected by immunoblotting using an anti-HA or anti-GFP antibody. The relative quantity (RQ) of indicated proteins was determined using the Quantity Tool within the BioRad Image Lab software. Amido black staining of RubisCo served as a loading control. The experiment was carried out at least 2 times with similar results. D, Accumulation of ubiquitinated *Nb*WRKY40-GFP in the presence of XopS-HA. *Nb*WRKY40-GFP was transiently expressed and the protein was stabilized either by treatment with 200 µM MG132 or by co-expression with XopS-HA (Input). Subsequently, the protein was purified from total plant extracts using GFP-trap (IP:GFP). Proteins were detected by immunoblotting with either anti-Ubiquitin, anti-GFP or anti-HA antibodies. The experiment was carried out at least 2 times with similar results.

Having established the interaction between XopS and WRKY40 and the role of the TF as a negative regulator of defense gene expression, we next sought to determine how XopS might affect the function of WRKY40 to support bacterial virulence. Some WRKY TFs undergo proteasomal turnover to regulate their activity (Miao and Zentgraf, 2010; Matsushita et al., 2013; Ye et al., 2018; Liu et al., 2021). Thus, we set out to determine whether WRKY40 undergoes proteasomal degradation in planta. To this end, we transiently expressed *Nb*WRKY40-HA in *N. benthamiana* leaves and 2 days later infiltrated the same leaves with the well-characterized proteasome inhibitor MG132. Subsequently, we took samples over a time course of 6 h. At 0 h *Nb*WRKY40-HA, protein was only detectable after long exposure of the membrane, while the signal was barely visible upon shorter exposure (Figure 10A). However, the *Nb*WRKY40-HA signal increased gradually over time, becoming easily visible even upon short exposure 6 h after treatment with MG132, indicating that *Nb*WRKY40-HA accumulates when the proteasome is inhibited. The long exposure revealed the presence of higher molecular weight *Nb*WRKY40-HA species at later time points of MG132 treatment, reminiscent of a poly-ubiquitination of the protein (Figure 10A). From these data we conclude that WRKY40 undergoes proteasomal degradation when transiently expressed in *N. benthamiana*.

To explore whether XopS affects WRKY40 protein accumulation, we monitored WRKY40 levels by immunoblotting in leaves of *N. benthamiana* either expressing *Nb*WRKY40 alone or in combination with XopS. Under the experimental conditions used, *Nb*WRKY40-HA was readily detectable only when the proteasome was inhibited by MG132, indicative for its proteasomal turnover (Figure 10B). However, when co-expressed with XopS-GFP, *Nb*WRKY40 accumulated to high levels, suggesting that the effector renders WRKY40 resistant to proteasomal degradation (Figure 10B). In contrast, *Nb*WRKY8 was not stabilized by co-expression with XopS (Figure 10C). The *R. solanacearum* T3E PopP2 is known to target a number of WRKY proteins in order to interfere with their function (Le Roux et al., 2015). However, co-expression with PopP2 did not affect *Nb*WRKY40 protein levels (Figure 10C). Thus, although we cannot exclude the possibility that XopS affects the stability of other WRKY proteins not tested here, the data suggest a certain degree of specificity of XopS on WRKY40 stability.

We did not detect any effect on overall proteasome activity in leaves transiently expressing XopS (Supplemental Figure S12), indicating that the effector does not have a general inhibitory effect on the proteasome like those previously reported for other T3Es (Üstün et al., 2013, 2014, 2016). Most proteasome substrates are marked for degradation by ubiquitin conjugation (Vierstra, 2009), and these conjugates can be reversed or modified by deubiquitinating enzymes that could interfere with the proteasomal degradation of target proteins. To investigate whether XopS affects the overall ubiquitination status of WRKY40, we expressed an *Nb*WRKY40-GFP fusion protein transiently in leaves of *N. benthamiana* and inhibited its proteasomal degradation either by MG132 treatment or by co-expression with XopS. We then immunopurified the protein using a GFP-trap and monitored it for ubiquitination using an ubiquitin-specific antibody. Inhibition of WRKY40 proteasomal degradation either by MG132 or by XopS allowed purification of *Nb*WRKY40-GFP in its ubiquitinated form (Figure 10D). This suggests that XopS does not stabilize WRKY40 by preventing its ubiquitination through, for instance, de-ubiquitination. However, the assay does not allow us to draw conclusions about WRKY40 ubiquitination levels or ubiquitin chain topology.

**Figure 11:**
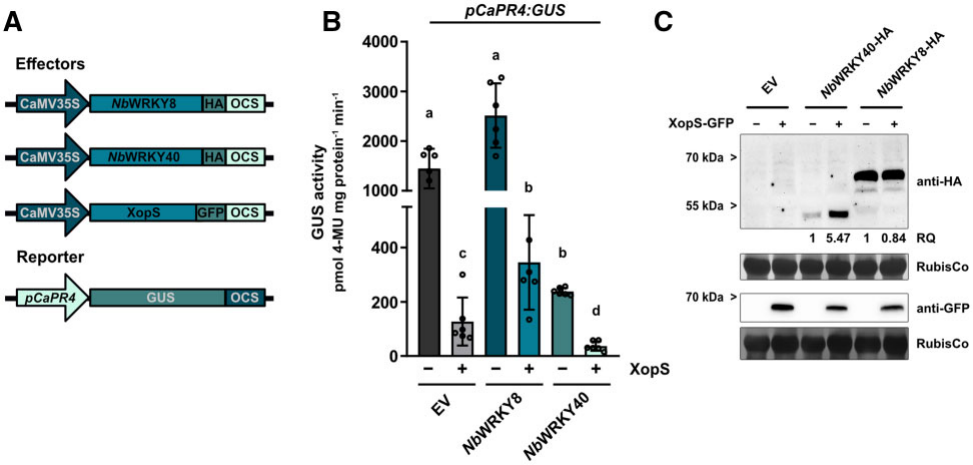
XopS and WRKY40 repress expression from the *CaPR4* promoter. A, Schematic diagrams of the effector and reporter constructs. The effector plasmids contain the WRKY TFs or XopS fused to the constitutive CaMV35S promoter and carry either a C-terminal HA-tag or a GFP-tag. The GUS reporter construct contains the 2 kb region upstream of the predicted translational start site of the *CaPR4* gene. OCS, terminator of the octopine synthase. B, Transactivation of the *pCaPR4*:GUS reporter by the TFs *Nb*WRKY8 or *Nb*WRKY40 either in presence (+) or absence (−) of XopS. Samples were taken 24 h after *Agrobacterium*-infiltration of indicated effector constructs and GUS activity is expressed in pmol 4-Methylumbelliferone mg protein^−1^ min^−1^. Bars represent the mean of n = 6 biological replicates ± sd. Letters over bars represent statistical significance determined by one-way ANOVA (*P* < 0.05).The experiment was carried out three times with similar results. C, Verification of effector protein expression in *pCaPR4:GUS* reporter gene analyses. Total protein extracts from *Agrobacterium*-infiltrated leaves were prepared 24 hpi and protein expression was detected by immunoblotting using an anti-HA or anti-GFP antibody. The RQ of indicated proteins was determined using the Quantity Tool within the BioRad Image Lab software. Amido black staining of RubisCo served as loading control. Leaf discs from the six biological replicates used for *pCaPR4:GUS* reporter gene analyses were pooled for the immunoblot analysis shown here.

Given the role of WRKY40 as a negative regulator of defense gene expression, we hypothesized that stabilization of WRKY40 through interaction of XopS would enhance the repressor function of the TF. To test this, we fused the 2 kb upstream region of the *CaPR4* gene (*pCaPR4*) to a GUS reporter gene and co-transformed the construct along with CaMV35S driven *NbWRKY40* or *NbWRKY8* in either the absence or presence of XopS in leaves of *N. benthamiana* (Figure 11A). The *pCaPR4* fragment mediated readily detectable GUS expression in the presence of the EV control (Figure 11B). Expression of the effector constructs and the stabilizing effect of XopS on WRKY40 were verified by immunoblotting (Figure 11C). XopS alone led to a significant reduction of GUS activity, likely stemming from the stabilization of endogenous WRKY40 protein, as XopS had no effect on *pCaPR4:GUS* activity in *WRKY40* silenced *N. benthamiana* plants (Supplemental Figure S13). *Nb*WRKY8 alone significantly induced *pCaPR4* driven GUS expression; however, XopS was able to reverse GUS induction, likely by stabilizing endogenous WRKY40 and thus partially outcompeting *Nb*WRKY8 from binding to the *PR4* promoter. The negative effect of XopS on reporter gene expression was enhanced by co-expression with *Nb*WRKY40, further supporting a synergistic effect of both proteins on the repression of gene expression (Figure 11B).

## Discussion

Disabling of host stomatal immunity is often a prerequisite for successful entry into leaf tissues by various phytopathogenic bacteria (Melotto et al., 2006). In this study, we provide evidence that the *Xcv* T3E XopS is a virulence factor required to overcome stomatal immunity. In pepper, XopS specifically stabilizes *Ca*WRKY40a, a negative regulator of defense gene induction, thus enhancing the repression of its target genes. As a result, SA signaling is attenuated while JA signaling is promoted to suppress stomatal closure, which facilitates *Xcv* infection.

Our data show that an *Xcv*Δ*xopS* strain achieved significantly lower population densities than WT *Xcv* when bacteria were dip-inoculated onto the leaf surface. This is in contrast to what has been described for pressure infiltration of leaf tissue, where an *Xcv* strain lacking XopS was not affected in bacterial multiplication relative to the WT (Schulze et al., 2012). It strongly resembles the infection of Arabidopsis with *Pst* DC3000 *COR*^*−*^ mutant bacteria (Mittal and Davis, 1995; Melotto et al., 2006) and suggests that, similar to COR, XopS interferes with an early defense response that is bypassed by syringe-inoculation of bacteria directly into the apoplast. Indeed, incubating pepper leaves with *Xcv* lacking XopS led to considerable stomatal closure, while the WT strain was able to prevent stomatal closure. Similar results were obtained upon flg22 treatment of either *N. benthamiana* or Arabidopsis leaves ectopically expressing XopS. This strongly suggests that XopS either is able to prevent stomatal closure or leads to a reopening of the stomata after their initial closure to facilitate bacterial tissue entry.

Although deletion of XopS does not affect multiplication of bacteria that are directly inoculated into the apoplast of pepper plants (Schulze et al., 2012), the effector is required for full symptom development and interferes with apoplastic immune responses. Like COR, XopS is required for chlorosis development and leads to a reduction of SA levels, resulting in decreased expression of SA-dependent genes. Syringe-inoculation of a nonpathogenic *Pst* strain into the leaves of Arabidopsis lines expressing XopS demonstrates that the effector supports bacterial growth and thus interferes with MTI during apoplastic defense. Moreover, XopS expression in Arabidopsis leads to leaf chlorosis, induction of early JA-responsive genes, and elevated JA levels. This phenotype is reminiscent of Arabidopsis lines expressing the T3E HopX1 from *Pta*, which is a protease that degrades JAZ proteins, a key family of JA repressors (Gimenez-Ibanez et al., 2014). *Pta* does not produce COR and thus it uses HopX1 as an alternative means to promote activation of JA-induced defenses and susceptibility in Arabidopsis. Similar to HopX1, XopS is able to complement the virulence defect of a *Pst* DC3000 *COR*^−^ strain in terms of bacterial multiplication and with respect to overcoming stomatal immunity. Thus, we propose that, similar to COR in *Pst* DC3000, *Xcv* uses XopS to activate JA signaling and to repress SA-related defense responses in order to promote disease susceptibility.

We show by several independent methods that XopS interacts with a subset of WRKY40 TFs, suggesting that XopS plays a role in modulating host gene expression. Notably, we observed XopS binding to only one of the two pepper WRKY40 orthologs, designated *Ca*WRKY40a in our study (Ca03g32070). In a similar vein, only one of three putative WRKY40 orthologs from *N. benthamiana* interacted with XopS in a Y2H assay.

In Arabidopsis, WRKY40 functions redundantly with WRKY18 and WRKY60 as a negative regulator of resistance against the hemibiotrophic bacterial pathogen *Pst* DC3000 (Xu et al., 2006). While neither the *wrky40* nor *wrky18* single mutants displayed increased resistance against *Pst*, a *wrky40 wrky18* double mutant showed reduced bacterial multiplication (Xu et al., 2006). Enhanced bacterial resistance in *wrky* knock-out lines was accompanied by elevated expression of *PR1* upon infection. Similar to the *wrky40 wrky18* double knock-out in Arabidopsis, VIGS of *CaWRKY40a* in pepper plants led to a >10-fold reduction of bacterial multiplication upon *Xcv* apoplast inoculation, suggestive of enhanced apoplastic defense against this hemibiotrophic pathogen. Reduced bacterial growth was correlated with enhanced expression of defense-related genes, indicating a role of *Ca*WRKY40a as a negative regulator of gene expression. This is in contrast to *Ca*WRKY40, which has been described as a positive regulator of defense gene expression (Dang et al., 2013).

The in vivo targets of *Ca*WRKY40a currently remain unknown. Based on previously reported positive regulation by other WRKY TFs and the in vitro binding of *Ca*WRKY40a to W-boxes within their promoter region, *CaJAZ8* and *CaPR4* can be considered bona fide *Ca*WRKY40a target genes. However, indirect effects on gene expression by other WRKY proteins or TFs from other families cannot be ruled out at this point. A recent genome-wide ChIP-seq analysis identified >1,400 in vivo target genes for WRKY40 in Arabidopsis with a clear preference for binding gene loci involved in early MTI perception and signaling (Birkenbihl et al., 2017). Thus, it can be expected that silencing of *CaWRKY40a* in pepper has far-reaching consequences for defense gene expression that result in enhanced immunity toward *Xcv*.

Several lines of evidence suggest that *Ca*WRKY40a negatively regulates defense gene expression, for example, the lack of transactivation activity in yeast and the ability to suppress the expression of a GUS reporter gene driven by a W-box containing promoter sequence. Thus, its induction by the defense hormone SA and during infection with virulent *Xcv* bacteria appears counterintuitive. However, a similar expression pattern has been observed for Arabidopsis *WRKY40*, and it has been proposed that this mode of regulation could ensure a dynamic function whereby some early MTI responses are rapidly activated but subsequently dampened to avoid unnecessary prolonged resource allocation to immunity (Pandey et al., 2010).

*CaWRKY40a* silenced plants display substantially reduced disease symptoms upon infection with *Xcv* compared to WT plants, which in turn develop severe chloroses correlated with losses in chlorophyll content. The development of chloroses is largely dependent on the translocation of XopS, and the phenotypic similarities between *XcvΔxopS* infected WT pepper plants and the *Xcv* infected *CaWRKY40a* silenced plants strongly suggest that XopS requires *Ca*WRKY40a to perform its virulence function. In support of this hypothesis, *Xcv* WT bacteria are unable to prevent stomatal closure in *CaWRKY40a* VIGS pepper plants, and these plants are also highly resistant to leaf surface inoculated bacteria. Transient expression of XopS does not prevent stomatal closing in *WRKY40* silenced *N. benthamiana* in response to a MAMP stimulus, further reinforcing a direct link between XopS’s virulence functions and its interaction with WRKY40. In pepper, the effects are specific to CaWRKY40a, as VIGS of CaWRKY40 neither affects the ability of *Xcv* to interfere with stomatal immunity nor results in higher resistance to the bacteria. This is in line with a specific interaction between XopS and CaWRKY40a, as well as a different role for this TF during infection relative to the positive regulator of gene expression CaWRKY40 (Dang et al., 2013).

The development of chloroses accompanied by a reduction in chlorophyll content is considered a hallmark response of the JA pathway (Creelman and Mullet, 1995). In Arabidopsis, WRKY40 targets the promoter region of several JA associated genes, including *JAZ8* (Pandey et al., 2010; Birkenbihl et al., 2017). Although *wrky40 wrky18* and *wrky40 wrky18 wrky60* mutants display increased resistance against biotrophic and hemi-biotrophic pathogens (Xu et al., 2006; Pandey et al., 2010), they show enhanced susceptibility to the necrotrophic pathogen *Botrytis cinerea* correlated with a reduced JA response (Xu et al., 2006). Thus, it was proposed that the three WRKY proteins function redundantly as negative regulators of SA-dependent pathways but play a positive role in JA-mediated pathways. Arabidopsis WRKY40 has been shown to bind to the promoter region of *JAZ8*, and the *wrky18 wrky40* double mutant constitutively expresses high levels of a number of JAZ family members (Pandey et al., 2010). *Ca*WRKY40a binds to a promoter region of the *JAZ8* ortholog from pepper containing two W-boxes, and *CaWRKY40a* silenced plants show increased expression of *JAZ8* upon infection with *Xcv* accompanied by a loss of JA-associated leaf phenotypes. Thus, similar to the findings in Arabidopsis, these data suggest that *Ca*WRKY40a positively affects JA signaling by directly reducing the expression of at least one of the negative regulators of the JA pathway. The phenotype of pepper plants infected with an *XcvΔXops* strain implies that XopS enhances the repressor activity of WRKY40, as these plants show less leaf chloroses then WT *Xcv* infected plants. In turn, either translocation of XopS during *Xcv* infection or ectopic expression of the effector protein in transgenic Arabidopsis lines leads to an induction of JA-mediated responses. This is in line with the observed effect of XopS on stomatal movement, whereby an induction of JA signaling results in an increased stomatal aperture in response to a MAMP stimulus (Melotto et al., 2006). Collectively our data suggest that by targeting WRKY40, XopS positively affects the repressor function of the TF to dampen SA-responses and at the same time induce JA-responses.

Targeting of WRKY TFs by bacterial T3Es is not without precedent. The *R. solanacearum* effector protein PopP2 is an acetyltransferase that binds and acetylates a range of Arabidopsis WRKY proteins (Le Roux et al., 2015). PopP2 acetylation within the WRKY domain of multiple defense-acting WRKY TFs interferes with their binding to W-box containing promoter elements, thereby reducing defense gene activation (Le Roux et al., 2015). Intriguingly, the group IIa WRKY TFs *At*WRKY40 and *At*WRKY60 do not interact with PopP2 and are not acetylation substrates for the effector protein. It has been suggested that PopP2 has evolved a degree of substrate discrimination, which avoids acetylation of negative regulators of defense whose inactivation would be disadvantageous for bacterial infection (Le Roux et al., 2015). Our data imply that binding of XopS to WRKY TFs has the opposite selectivity relative to PopP2. XopS interacts with WRKY40 orthologs from different plants but does not bind to *Nb*WRKY8, a group I WRKY TF, which promotes defense gene induction (Ishihama et al., 2011). Similar to the effect of acetylation on negative regulators of defense gene expression by PopP2, stabilization of positive regulators by XopS would counteract the virulence activity of the effector. Thus, stabilization of negatively acting group IIa WRKY TFs, such as WRKY40, by XopS reduces defense gene induction and at the same time induces JA signaling, providing an additional means to counteract SA-mediated immunity.

The mechanism through which XopS stabilizes the WRKY40 protein is currently unknown. The available data suggest that WRKY40 is subject to proteasomal turnover, likely as a means to balance its repressor activity during active defense, and that XopS interferes with degradation of the TF by the proteasome. Several other WRKY TFs have been described as being degraded by the proteasome to regulate expression of their target genes in response to a range of stimuli, including immunity (Miao and Zentgraf, 2010; Matsushita et al., 2013; Ye et al., 2018; Liu et al., 2021). E3-ubiquitin ligases from different families are involved in WRKY ubiquitination; for instance, the *Phytophthora sojae* effector protein Avr1d was recently demonstrated to repress the E3-ligase activity of *Gm*PUB13 to facilitate infection (Lin et al., 2021). However, it seems unlikely that XopS acts as inhibitor of the E3-ligase catalyzing WRKY40 ubiquitination, as this would not require a direct interaction with the TF; moreover, our data suggest that XopS stabilized WRKY40 is still conjugated to ubiquitin, indicating that it has been modified by an E3-ligase activity. Ubiquitin modifies protein substrates mostly in the form of a K48 linked polyubiquitin chain, which serves as a signal for proteasome degradation (Finley, 2009). However, other types of ubiquitination may also occur, including monoubiquitination and polyubiquitination, in which ubiquitin chains are formed through the linkage of lysine residues other than K48 of the ubiquitin molecule, that is K6‐, K11-, K27‐, K29‐, K33‐, and K63‐linked ubiquitination, or through the C‐ or N‐terminus of the ubiquitin moieties (i.e. linear ubiquitination) (Kulathu and Komander, 2012). Although not very well understood, many nonK48-linked types of ubiquitination appear to serve as nonproteolytic regulatory modifications often related to chromatin organization, transcription, DNA repair, and protein trafficking (Mukhopadhyay and Riezman, 2007). It is currently unclear how these atypical ubiquitin conjugates are assembled and whether XopS influences the ubiquitin chain topology of WRKY40 either directly or by recruiting auxiliary proteins to the XopS/WRKY40 complex.

**Figure 12:**
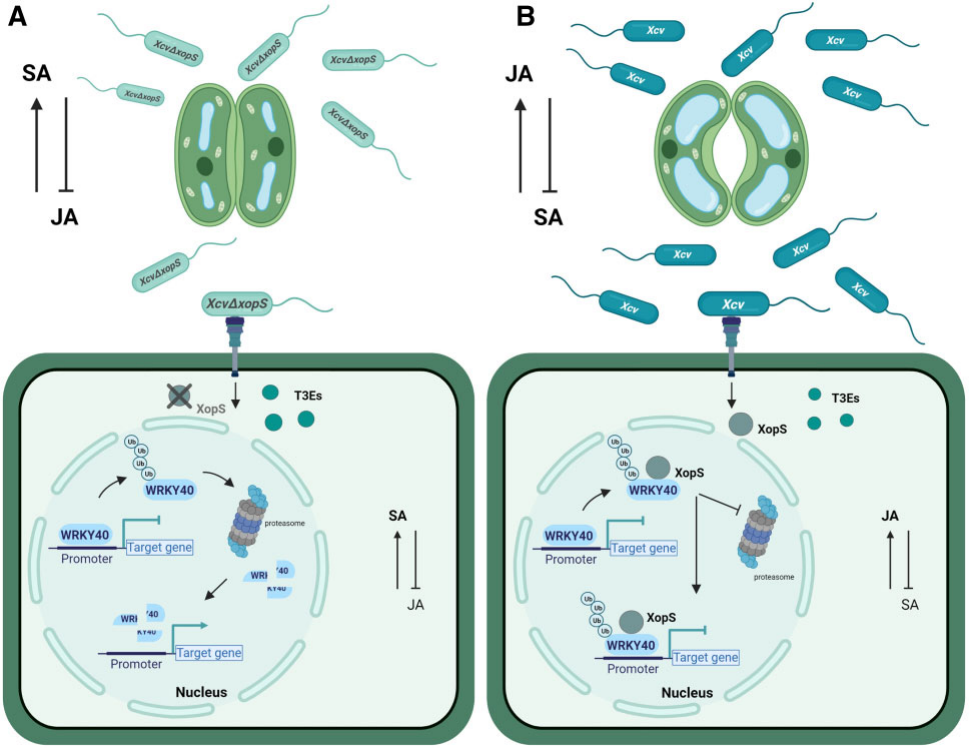
Working model of how XopS promotes *Xcv* virulence. A, Upon infection of pepper plants with an *Xcv* strain lacking XopS, WRKY40 ubiquitination results in its degradation by the proteasome and leads to the de-repression of its target genes. This enables the induction of SA-dependent gene expression, such as *CaPR1*, and at the same time represses JA-dependent responses through increased expression of negative regulators, including *CaJAZ8*. As a result, stomata close in response to MAMP perception and the plant mounts apoplastic defense responses. B, In contrast, WT *Xcv* delivers XopS into the host cell, which prevents degradation of WRKY40 through physical interaction. Stabilized WRKY40 attenuates the induction of SA-dependent immunity and also mediates decreased expression of *CaJAZ8*. The latter results in an activation of JA-responses further antagonizing SA-mediated defense, including interference with stomatal closure to facilitate bacterial tissue entry.

In conclusion, the data discussed above led us to propose a working model of how XopS promotes bacterial virulence (Figure 12). Future experiments will have to investigate the biological mechanism of how XopS prevents proteasomal degradation of WRKY40 and reveal possible enzymatic activities of the effector. It will be interesting to see whether the mechanism of stabilizing host TFs to subvert defense responses is widely used by pathogens.

## Materials and methods

### Plant material and growth conditions

Pepper (*C.* *annuum* cv. Early Cal Wonder) and *Nicotiana benthamiana* and *N. benthamiana roq1* plants were grown in soil in a growth chamber with daily watering, and subjected to a 16-h light:8-h dark cycle (25°C:20°C) at 240–300 µmol m^−2^ s^−1^ light (Metal Halide Lamp MT400DL/BH; Iwasaki Electric Co., Japan) and 75% relative humidity. Transgenic β-estradiol inducible XopS-GFP Arabidopsis (*A.* *thaliana*) lines were generated by transformation of Col-0 WT plants with a plasmid containing the entire XopS coding region inserted into binary vector pABindGFP (Bleckmann et al., 2010) using the floral dip method (Clough and Bent, 1998). For the selection of transgenic plants, seeds of T0 plants were sterilized and sown onto Murashige and Skoog medium (Sigma–Aldrich, St. Louis, MO, USA) supplemented with Gamborg’s vitamin solution (1:1.000) and 20 µg/mL hygromycin B (Roth, Karlsruhe, Germany). Primary transformants were allowed to self-fertilize and were propagated into the homozygous T3 generation. Arabidopsis plants were grown in soil in an 8-h light:16-h dark cycle (22°C:18°C) at 80 µmol m^−2^ s^−1^ light and 70% relative humidity. Transgene expression was induced 6 weeks after germination by spraying with 50 µM β-estradiol (Sigma-Aldrich) and 0.1% (v/v) Tween-20. Control plants were sprayed with 0.1% (v/v) EtOH in water and 0.1% (v/v) Tween-20.

### Plasmid construction

To generate plasmids containing the corresponding gene of interest, the entire open-reading frame either with or without the stop codon was amplified from cDNA. The resulting fragments were inserted into the pENTR-D/TOPO vector according to the manufacturer’s instructions (ThermoFisher, Waltham, MA, USA) and verified by sequencing. For Y2H experiments, fragments containing a stop codon were recombined into Gateway-compatible versions of the GAL4 DNA BD vector pGBT-9 and the activation domain (AD) vector pGAD424 (Clontech, Mountain View, CA, USA) using L/R-clonase (ThermoFisher). To generate translational fusions between the protein of interest and GFP, CDSs without stop codon were recombined as described above into the binary vector pK7FWG2 (Karimi et al., 2002). To tag proteins with hemagglutinin (HA), fragments were inserted into pGWB614 (Nakamura et al., 2010). To generate the *Nb*WRKY8–HA fusion protein, the NEBuilder recombinational cloning kit (New England Biolabs [NEB], Ipswich, MA, USA) was used. Constructs for protein expression in *Escherichia coli* were either inserted into a Gateway-cloning-compatible version of pMal-C2 (NEB) to create a fusion to the MBP or into pDEST15 for fusion to GST (ThermoFisher). To create GUS reporter constructs, either an oligonucleotide containing four W-boxes and the minimal CaMV 35S promoter or the 2 kb region upstream of the translational start site of *CaPR4* were inserted in front of the GUS reporter gene in vector pRB-bar-GUS using the NEBuilder recombinational cloning kit (NEB).

Constructs for VIGS in pepper or *N. benthamiana* were generated by recombining a PCR fragment of the gene of interest from pENTR-D/TOPO into pTRV2-Gateway (Liu et al., 2002). To generate the construct for VIGS of *CaWRKY40*, the NEBuilder recombinational cloning kit (NEB) was used.

Sequences of oligonucleotides used for cloning are listed in Supplemental Table S2.

### Phylogenetic analysis

Phylogenetic analysis was performed using the MEGA X software package (Kumar et al., 2018). Arabidopsis, pepper (*C.* *annuum*) and tobacco (*N.* *tabacum*) WRKY40 sequences were collected from NCBI (https://www.ncbi.nlm.nih.gov/). *Nicotiana* *benthamiana* WRKY sequences were retrieved from the Solgenomics database (https://solgenomics.net/). Proteins were aligned in MEGA X. An unrooted WRKY phylogenetic tree was inferred by using the Maximum Likelihood method and JTT matrix-based model. The tree with the highest log likelihood (−5,087.96) was selected. The alignment and tree information are provided in Supplemental File S1.

### Y2H analysis

Y2H techniques were performed according to the yeast protocols handbook and the Matchmaker GAL4 Two-hybrid System 3 manual (both Clontech, Heidelberg, Germany) using the yeast reporter strains AH109 and Y187. To identify XopS interacting proteins, a GAL4 AD-domain tagged cDNA library from *N.* *tabacum* (Börnke, 2005) was screened as detailed in Üstün et al. (2013).

### Yeast transactivation assay

WRKY proteins used for this experiment were fused into the pGBT9 vector (containing GAL4 DNA-BD) and transformed into yeast strain Y190. Transformed clones were selected on SCAD yeast growth medium (6.7 g/L yeast nitrogen base, 20 g/L glucose, 0.67 g/L amino acid mix, 200 mg/L adenine hemisulfate; pH 5.8) lacking Trp (-T) and growing cells were spotted onto selective medium lacking Trp and His (-TH), supplemented with 10 mM 3-amino-1,2,4-triazole. Three days later, *HIS3* reporter gene activity was monitored by the ability of transformed cells to grow on SCAD-TH plates. Additionally, cells growing on selective medium were further tested for their ability to induce the *lacZ* reporter gene using a filter lift assay. The empty pGBT9 vector was used as a negative control and the pGAL4 vector containing full length GAL4 was used as a positive control. Yeast cells were grown at 30°C.

### Bacterial strains and plant infection assays

Strains used in this study were as follows: *X.* *campestris* pv. *vesicatoria* (*Xcv* WT), *XcvΔhrpF* (type-3 secretion-deficient mutant), *XcvΔxopS*, complementation strain *XcvΔxopS*(*XopS-HA*), *P.* *syringae* pv. *tomato* strain DC3000 *PstΔhrcC* (type-3 secretion-deficient mutant), and a *COR*^*−*^  *P.* *syringae* pv. *tomato* DC3000 strain (Gimenez-Ibanez et al., 2014). The *XcvΔxopS* deletion strain and its *XcvΔxopS*(*XopS-HA*) complementation strain were constructed as described in Üstün et al. (2013) using the primers listed in Supplemental Table S2. Bacteria were grown overnight in KingsB medium (20 g/L glycerol, 40 g/L peptone, 10 mL/L 10% (w/v) K_2_HPO_4_, 10 mL/L 10% (w/v) MgSO_4_) containing appropriate antibiotics at 28°C with shaking.

For in planta growth assays using syringe-inoculation, leaves from 4 to 5-week-old pepper plants were infiltrated with the different *Xcv* strains at OD_600_ = 0.0001 (1 × 10^5^ CFU/mL) using a 1 mL sterile needleless syringe. Leaves from 6-week-old Arabidopsis plants were inoculated with *PstΔhrcC* at OD_600_ = 0.00002 (1 × 10^4^ CFU/mL). Bacterial suspensions were prepared in 10 mM MgCl_2_. For dip-inoculation assays of pepper plants with *Xcv* strains, bacterial suspensions were prepared at OD_600_ = 0.2 (1 × 10^8^ CFU/mL) in 10 mM MgCl_2_ supplemented with 0.02% (v/v) Silwet l-77 (Helena Chemical Company, Collierville, TN, USA). One leaf per plant (4–5 weeks old) was completely submerged into the indicated bacterial suspension for 1 min. Leaves were then covered with a moisturized plastic bag for 24 h to ensure 100% humidity. Plants were kept in the growth chamber under a 16-h light:8-h dark cycle (25°C:20°C) at 240–300 µmol m^−2^ s^−1^ light and 75% relative humidity. For dip-inoculation assays of Arabidopsis plants with *Pst* strains, bacterial suspensions were prepared at OD_600_ = 0.2 (1 × 10^8^ CFU/mL) in 10 mM MgCl_2_ supplemented with 0.02% (v/v) Silwet l-77. Rosette leaves from 6-week-old plants were completely submerged into the indicated bacterial suspension for 30 s. Plants were kept in the growth chamber under a 8-h light:16-h dark cycle (20°C:18°C) at 80-µmol m^−2^ s^−1^ light and 70% relative humidity.

For determination of bacterial titer of each analyzed strain, one or two leaf discs (1 × 0.5 cm^2^ for pepper, 2 × 0.22 cm^2^ for Arabidopsis) per biological replicate were collected, surface sterilized and ground in 10 mM sterile MgCl_2_. Two technical replicates per biological replicate were prepared. Serial dilutions were spotted onto KingsB plates supplemented with appropriate antibiotics. For infection assays used for analysis of marker gene expression, leaves from 4 to 5-week-old pepper plants were infiltrated with the different *Xcv* strains at OD_600_ = 0.2 (1 × 10^8^ CFU/mL) in 10 mM MgCl_2_. About 10 mM MgCl_2_ was used as mock control. At least three biological replicates were used per experiment unless otherwise stated.

### Transient expression of proteins in *N. benthamiana*

For infiltration of *N. benthamiana* leaves, *A.* *tumefaciens* (*Agrobacterium*) strain C58C1 was infiltrated into the abaxial air space of 4-week-old plants, using a 1 mL sterile needleless syringe. *Agrobacteria* were cultivated overnight in YEB medium (5 g/L Difco bovine extract, 1 g/L yeast extract, 1 g/L bacto peptone, 5 g/L saccharose, 2 mM MgSO_4_) containing appropriate antibiotics at 28°C with shaking. The cultures were harvested by centrifugation and the pellet was resuspended in infiltration buffer (10 mM MgCl_2_, 10 mM MES pH 5.7, 200 µM acetosyringone; Sigma–Aldrich) and incubated for 2 h in the dark with shaking prior to infiltration. Cultures containing different transgenes were infiltrated at OD_600_ = 0.5. *Agrobacteria* carrying the silencing inhibitor P19 were co-infiltrated at OD_600_ = 0.3. Infiltrated plants were kept in the growth chamber under 16-h light:8-h dark cycle (25°C:20°C) at 240–300 µmol m^−2^ s^−1^ light and 75% relative humidity.

### Virus-induced gene silencing

VIGS was performed as described previously (Üstün et al., 2013). Briefly, *Agrobacterium* strains carrying the pTRV1 vector (Liu et al., 2002) and either pTRV2-*GFP* or pTRV2-*CaWRKY40a* (OD_600_ = 1.0) were mixed in a 1:1 ratio, and the mixture was infiltrated into cotyledons of 10-day-old pepper plants using a 1 mL sterile syringe without a needle. For each experiment, 10 plants were infiltrated with pTRV2-*GFP* (control) and 50 plants were infiltrated with pTRV2-*CaWRKY40a*. For VIGS in *N. benthamiana*, *Agrobacterium* strains carrying the pTRV1 vector and either the pTRV2 EV (pTRV2) or pTRV2-*NbWRKY40* (OD_600_ = 1.0) were mixed in a 1:1 ratio, and the mixture was infiltrated into two lower leaves of 2-week-old *N. benthamiana* plants (four-leaf-stage) using a 1 mL sterile needleless syringe. For each experiment, 10 plants were infiltrated with pTRV2 (control) and 30 plants were infiltrated with pTRV2-*NbWRKY40*. The *Agrobacterium*-inoculated plants were kept in the dark for 56 h and then grown under a 16-h light:8-h dark cycle (25°C:20°C) at 240–300 µmol m^−2^ s^−1^ light and 75% relative humidity. Silenced plants were analyzed two to 3 weeks postinoculation. The mRNA levels of silenced plants were checked via quantitative real-time PCR upon treatment of one excised leaf per plant with 5 mM SA for 4 h to trigger *WRKY40* gene expression. *WRKY40* mRNA levels in pTRV2-*CaWRKY40a* pepper or pTRV2-*NbWRKY40 N. benthamiana* plants were compared to mRNA levels in pTRV2-GFP or pTRV2 plants, respectively.

### RNA extraction and RT-qPCR

Total RNA was isolated from leaf material and then treated with RNase-free DNase to degrade any remaining DNA. RNA concentrations were measured using a microplate reader (Tecan Infinite 200 PRO). For RT-qPCR, first-strand cDNA synthesis was performed from 1 µg of total RNA using Revert-Aid reverse transcriptase (ThermoFisher) and the cDNAs were amplified using SensiFAST SYBR Lo-ROX Mix (Bioline, London, UK) in the AriaMx Realtime PCR System (Agilent Technologies, Santa Clara, CA, USA) as previously described (Arsova et al., 2010). At least three biological and two technical replicates were used for each analysis. The transcript level was standardized based on cDNA amplification of the reference genes stated in the respective figure legends. Primer sequences are provided in Supplemental Table S2.

### Stomatal assays

Leaf discs were cut from leaves of four to 5-week-old pepper and *N. benthamiana* plants or from 6-week-old Arabidopsis plants and floated on water for 2 h to allow maximum stomatal opening. After 2 h, *N. benthamiana* or Arabidopsis leaf discs were floated on water supplemented with 25 µM flg22 (Hycultec, Beutelsbach, Germany) or 50 µM ABA (Sigma–Aldrich) for 2 h as described in Lozano-Durán et al. (2014). For stomatal assays using bacterial suspensions, Arabidopsis or pepper leaf discs were floated on water supplemented with a bacterial suspension of different *Pst* or *Xcv* strains at OD_600_ = 0.2 (1 × 10^8^ CFU/mL) for 3 or 2 h, respectively. Leaf discs were kept under light (80 µmol m^−2^ s^−1^ for Arabidopsis and 240–300 µmol m^−2^ s^−1^ for pepper) over the whole duration of the experiment and were removed from the growth chamber only right before the microscopic analysis. At the indicated time points, leaf discs were moved to a microscope slide containing sterilized water and the lower epidermis of the leaf discs was observed under a digital microscope VHX-7000 (Keyence, Osaka, Japan; version 1.4.17.3). For each experiment, stomatal apertures (width/length) of ∼100 stomata per treatment were measured using the onboard software of the microscope. Completely closed stomata were reported as a value of 0 μm. At least three biological replicates were used per experiment unless otherwise stated.

### Chlorophyll measurement

For determination of total chlorophyll (Chl *a* + *b*) content, four leaf discs were cut from each biological replicate and incubated in 1 mL of 80% (v/v) acetone overnight at 4°C with shaking. Measurements were made by pipetting 200 µL sample into a 96-well flat bottom polystyrene plate (Sarstedt, Nümbrecht, Germany) and absorption at wavelength 663 nm (Chl *a*) and wavelength 645 nm (Chl *b*) was read in a Tecan Infinite 200 PRO microplate reader. About 80% (v/v) acetone was used as blank. Total chlorophyll was calculated using the formula described in Lichtenthaler (1987). Chlorophyll content in control plants was set to 100%. Two technical replicates per biological replicate were measured. At least six biological replicates were used per experiment unless otherwise stated.

### Plant hormone analysis

SA was extracted from frozen leaf material and analyzed by an ultra-high-performance liquid chromatography (HPLC) system (Agilent Technologies) coupled to an Agilent 6530 quadrupole time of flight-mass spectrometer (Agilent Technologies) using the internal standard d4-SA, as described previously (Nietzsche et al., 2018). JA was extracted from the plant matrix and analyzed by HPLC coupled to a QTrap mass spectrometer. In brief, 5 mg of freeze-dried and homogenized sample material was extracted with a methanol/water (60:40, v/v) solution. Deuterated JA (d5-JA) was added as an internal standard. Analysis was performed using an Agilent Technologies 1260 Infinity HPLC coupled to Q-Trap 6500 ESI-MS/MS system (Sciex, Framingham, MA, USA). The analytes were separated on an Eclipse Plus C18 column (Agilent Technologies, Waldbronn, Germany) using water (+ 0.1% (v/v) acetic acid) and acetonitrile (+0.1% (v/v) ultrapure water) as a mobile phase in a gradient mode. Ionization was performed with electron spray ionization (ion source temperature, 500°C; ion spray voltage, −4,500 V; curtain gas, 50 psi; nebulizer gas, 50 psi; auxiliary gas, 65 psi) in the negative ionization mode. Multiple reaction monitoring (MRM) mode was used for the measurement, and the transitions of JA (209 → 59; collision energy, −16 V, entrance potential −5 V; cell exit potential −15 V; declustering potential −50 V) and d5-JA (214 → 62; collision energy −16 V, entrance potential −5 V; cell exit potential −15 V; declustering potential −50 V) were used for quantification. The concentrations were calculated based on external calibration curves of JA and the recovery of the internal standard (d5-JA).

### Immunoblotting

Transiently expressed proteins were extracted from *N. benthamiana* leaf material using plant protein extraction buffer (50 mM Tris–HCl pH 7.5, 150 mM NaCl, 5 mM EDTA, 1 mM NaF, 10 mM DTT, 0.1% (v/v) Triton X-100 and 1% (v/v) protease inhibitor cocktail; Sigma–Aldrich). Protein extracts were boiled at 95°C for 10 min in 1× sodium-dodecyl sulfate (SDS) loading buffer (4×; 200 mM Tris–HCl pH 6.8, 0.4 M DTT, 40% (v/v) glycerol, 6 mM bromophenol blue and 8% (w/v) SDS) and then subjected to SDS-polyacrylamide gel electrophoresis. Separated proteins were transferred onto nitrocellulose membrane (Amersham biosciences, Amersham, UK), blocked with 5% (w/v) skimmed milk in TBS-T and incubated with respective antibodies in 1% (w/v) skimmed milk in TBS-T. Antibodies used in this study were as following: horseradish peroxidase-conjugated anti-GFP (1:1,000; Santa Cruz Biotechnology Inc., Dallas, TX, USA; cat. no. sc-9996 HRP), anti-HA (1:500; Merck Chemicals GmbH, Billerica, MA, USA; cat. no. 12013819001) and anti-Ubiquitin (1:500; Santa Cruz Biotechnology Inc., cat. no. sc-8017 HRP). Proteins were detected using the Clarity Western ECL substrate (BioRad, Hercules, CA, USA) and chemiluminescence was detected using a ChemiDoc imager (BioRad).

### In planta GFP pull-down

Approximately 1 g of leaf material was ground to a fine powder in liquid nitrogen and homogenized in 2 mL of extraction buffer (25 mM Tris–HCl, pH 7.5, 150 mM NaCl, 10% (v/v) glycerol, 1 mM DTT, 1 mM EDTA, 0.5% (v/v) Triton X-100 and 1% (v/v) protease inhibitor cocktail; Sigma–Aldrich). Insoluble debris was pelleted by centrifugation for 20 min with 4,000 rpm at 4°C. IP was performed by adding 50 µL of GFP-Trap coupled to magnetic beads (ChromoTek, Munich, Germany) and samples were incubated for 2 h at 4°C with continuous rotation. Beads were subsequently washed five times with Tris-buffered saline containing 0.5% (v/v) Triton X-100, and immunoprecipitates were eluted with 70 μL of 1× SDS loading buffer at 95°C for 10 min.

### In vitro pull-down

Recombinant MBP-*Nb*WRKY40, MBP-*Nb*WRKY8, or MBP alone from *E. coli* lysates were immobilized on amylose resins (NEB), incubated for 1 h at 4°C with purified GST-XopS or GST alone, eluted, and analyzed by immunoblotting using either anti-GST antibody (1:1,000, horseradish peroxidase-conjugated; Santa Cruz Biotechnology Inc.; cat. no. sc-138 HRP) or anti-MBP antibody (1:10,000; NEB, cat. no. E8032S; used with 1:10,000 secondary horseradish peroxidase-conjugated anti-mouse antibody; ThermoFisher; cat. no. 31430).

### GUS activity measurement

For in planta GUS reporter assays, 4-week-old *N. benthamiana* plants were inoculated with *Agrobacteria* carrying indicated effector constructs, alongside *Agrobacteria* carrying indicated reporter constructs at OD_600_ = 0.5. *Agrobacteria* carrying the silencing inhibitor P19 were co-infiltrated at OD_600_ = 0.3. After 24 or 48 h, four leaf discs (0.5 cm^2^) per sample were ground in 120 µL GUS extraction buffer (50 mM sodium phosphate buffer pH 7.0, 10 mM DTT, 1 mM Na_2_EDTA, 0.1% (w/v) SDS and 0.1% (v/v) Triton X-100), incubated for 20 min on ice and then centrifuged at 13,000 rpm for 10 min at 4°C. Protein concentrations were determined using Bradford reagent (BioRad), and 200 µg of protein extract were preincubated in 450 µL GUS assay buffer for 10 min at 22°C to guarantee equal protein amounts incubated with the substrate. The reaction was started by adding 25 µL of 20 mM 4‐Methylumbelliferyl‐β-D‐glucuronide hydrate (Sigma–Aldrich) substrate to the samples and a 20 µL aliquot was immediately added to 180 µL stop buffer (0.2 M Na_2_CO_3_) which was used as a time point 0 control. The rest of the mixture was incubated at 37°C for 1 h and the reaction was stopped by again adding 20 µL aliquots to 180 µL stop buffer. GUS activity was determined by measuring the fluorescence (excitation wavelength 365 nm and emission wavelength 460 nm) of produced 4-Methylumbelliferone (4-MU) in a 96-well polystyrene microwell plate (Nunc F96 black flat bottom; ThermoFisher) by the Tecan Infinite 200 PRO microplate reader. A 4-MU standard curve was used to calculate the produced amount of 4-MU per unit of time and GUS activity is given in pmol (picomol) 4-MU mg protein^−1^ min^−1^. Protein expression of effectors was verified by immunoblotting.

### Fluorescent EMSA

DNA binding reactions for the fluorescent EMSA were carried out in 25 mM HEPES-KOH pH 7.6, 40 mM KCl, 1 mM DTT and 10% (v/v) glycerol. Fifty nanograms of Cy5-labeled probe DNA was incubated with the indicated recombinant proteins in a total volume of 20 μL for 30 min at room temperature. MBP-*Ca*WRKY40a and MBP recombinant proteins were produced in *E. coli* and purified using amylose resin (NEB). Target probes for MBP-*Ca*WRKY40a upstream regions consisted of 150 bp fragments containing two predicted W-boxes that were amplified by PCR using Cy5-labeled primers and gel purified. As competitor DNA, the same fragments amplified using unmodified primers were used and added in 50-fold (50×) excess over the labeled probe. Primer sequences can be found in Supplemental Table S2. 1× Orange-G (0.25% (w/v) Orange-G and 30% (v/v) glycerol) was used as loading dye. The DNA–protein complexes were resolved on 5% nondenaturing polyacrylamide gels (BioRad) and visualized using an Octoplus Q-Plex Imaging System (Intas, Homburg, Germany).

### Drug treatment

For analyzing protein stability in *N. benthamiana* plants transiently expressing binary *Nb*WRKY40 constructs, leaves were infiltrated 42 hpi with 200 µM MG132 (Sigma–Aldrich). Six hours later, leaf material was harvested and processed.

### Proteasome activity measurement

The experiment was performed as previously described (Üstün and Börnke, 2017). Proteasome activity is expressed in percentage relative to the control treatment (set to 100%).

### Statistical analysis and data presentation

Depending on the experiment, statistical significances were based on either Student’s *t* test or one-way ANOVA followed by Tukey’s multiple comparisons test. For statistical analysis of data shown in Figure 11B, data were transformed by Tukey’s Ladder of Powers approach using the R package rcompanion (http://rcompanion.org/handbook/G_14.html). ANOVA and Student’s t test tables are provided in Supplemental File S2. The model generated to illustrate XopS’ mode of action was created with BioRender.com. Each experiment was repeated at least twice unless otherwise stated. The number of biological replicates (individual plants, *n*) is stated in the figure legends.

## Accession numbers

Sequence data for genes relevant to this article can be found in the Arabidopsis Genome Initiative, GenBank/EMBL or Solgenomics databases under the following accession numbers: *AtWRKY40*, AT1G80840; *CaWRKY40*, XP_016578457; *CaWRKY40a*, XP_016562883; *Ca*WRKY1, XP_016582129.1; *NtWRKY40*, XP_016457007; *NbWRKY40*, Niben101Scf06091g04005.1; *Nb*WRKY40a, Niben101Ctg16115g00003.1; *Nb*WRKY40e, Niben101Scf04944g05002.1; *NbWRKY8*, AB445392; *CaCDPK15*, XM_016716796.1; *CaPR1*, AY560589.1; *CaPR4*, JX030397.1; *CaJAZ8*, XM_016682106.1; and *XopS*, CAJ21955.

## Supplemental data

The following materials are available in the online version of this article.

**Supplemental Figure S1.** XopS contributes to *Xcv* symptom development on susceptible pepper plants and alters SA responses.

**Supplemental Figure S2.** Transient expression of XopS in *N. benthamiana* inhibits stomatal closure in response to flg22.

**Supplemental Figure S3.** Verification of ectopic XopS protein expression.

**Supplemental Figure S4.** Sequence relationship of different WRKY proteins.

**Supplemental Figure S5.** XopS does not interact with WRKY proteins other than WRKY40 in yeast.

**Supplemental Figure S6.** Basal gene expression of pepper WRKY40a and WRKY40.

**Supplemental Figure S7.** Verification of effector protein expression in *W-box:GUS* reporter gene analyses.

**Supplemental Figure S8.** Verification of *CaWRKY40a* downregulation in VIGS pepper plants used for defense gene expression analyses.

**Supplemental Figure S9.** Mutation of W-boxes abolishes binding of *Ca*WRKY40a to promotor fragments of *Ca*JAZ8 and *Ca*PR4.

**Supplemental Figure S10.** Induction of NbWRKY40 expression upon flg22 treatment.

**Supplemental Figure S11.** Verification of protein expression in pTRV2 (EV silencing; control) and pTRV2-NbWRKY40 (*NbWRKY40* silencing) VIGS *N. benthamiana* plants transiently expressing either GFP, XopS-GFP, or HopX1-GFP.

**Supplemental Figure S12.** Expression of XopS does not affect proteasome activity.

**Supplemental Figure S13.** The ability of XopS to repress *pCaPR4:GUS* reporter gene expression depends on WRKY40.

**Supplemental Table S1.** Candidate XopS interaction partners from the Y2H screening.

**Supplemental Table S2.** Primers used for RT-qPCR, EMSA, and cloning.

**Supplemental File S1.** Fasta file of the alignment used for the phylogenetic analysis and a tree file corresponding to the phylogenetic tree shown in Supplemental Figure S4.

**Supplemental File S2.** ANOVA and Student’s *t* test tables.

## Supplementary Material

koac032_supplementary_data

## Notes

M.R., S.Ü., and F.B. conceived and designed the experiments. M.R., S.Ü., T.G., D.S., M.F., and F.B. performed the experiments. M.R., S.Ü., S.B., and F.B. analyzed the data. S.S. provided unpublished materials. M.R. created the figures. M.R. and F.B. wrote the manuscript with input from all authors.

The author responsible for distribution of materials integral to the findings presented in this article in accordance with the policy described in the Instructions for Authors (https://academic.oup.com/plcell) is: Frederik Börnke (boernke@igzev.de).

## References

[koac032-B1] Arsova B , Hoja U, Wimmelbacher M, Greiner E, Üstün S, Melzer M, Petersen K, Lein W, Börnke F (2010) Plastidial thioredoxin z interacts with two fructokinase-like proteins in a thiol-dependent manner: evidence for an essential role in chloroplast development in Arabidopsis and *Nicotiana benthamiana*. Plant Cell 22: 1498–1515 20511297 10.1105/tpc.109.071001 PMC2899873

[koac032-B2] Birkenbihl RP , Kracher B, Roccaro M, Somssich IE (2017) Induced genome-wide binding of three Arabidopsis WRKY transcription factors during early MAMP-triggered immunity. Plant Cell 29: 20–38 28011690 10.1105/tpc.16.00681 PMC5304350

[koac032-B3] Bleckmann A , Weidtkamp-Peters S, Seidel CAM, Simon R (2010) Stem cell signaling in Arabidopsis requires CRN to localize CLV2 to the plasma membrane. Plant Physiol 152: 166–176 19933383 10.1104/pp.109.149930 PMC2799354

[koac032-B4] Boch J , Bonas U (2010) Xanthomonas AvrBs3 family-type III effectors: discovery and function. Ann Rev Phytopathol 48: 419–436 19400638 10.1146/annurev-phyto-080508-081936

[koac032-B5] Börnke F (2005) The variable C-terminus of 14-3-3 proteins mediates isoform-specific interaction with sucrose-phosphate synthase in the yeast two-hybrid system. J Plant Physiol 162: 161–168 15779826 10.1016/j.jplph.2004.09.006

[koac032-B7] Clough SJ , Bent AF (1998) Floral dip: a simplified method for Agrobacterium-mediated transformation of *Arabidopsis thaliana*. Plant J 16: 735–743 10069079 10.1046/j.1365-313x.1998.00343.x

[koac032-B8] Creelman RA , Mullet JE (1995) Jasmonic acid distribution and action in plants: regulation during development and response to biotic and abiotic stress. Proc Natl Acad Sci USA 92: 4114–4119 11607536 10.1073/pnas.92.10.4114 PMC41895

[koac032-B9] Dang FF , Wang YN, Yu L, Eulgem T, Lai Y, Liu ZQ, Wang X, Qiu AL, Zhang TX, Lin J, et al (2013) CaWRKY40, a WRKY protein of pepper, plays an important role in the regulation of tolerance to heat stress and resistance to Ralstonia solanacearum infection. Plant Cell Environ 36: 757–774 22994555 10.1111/pce.12011

[koac032-B10] Du M , Zhai Q, Deng L, Li S, Li H, Yan L, Huang Z, Wang B, Jiang H, Huang T, et al (2014) Closely related NAC transcription factors of tomato differentially regulate stomatal closure and reopening during pathogen attack. Plant Cell 26: 3167–3184 25005917 10.1105/tpc.114.128272 PMC4145139

[koac032-B11] Eulgem T , Rushton PJ, Robatzek S, Somssich IE (2000) The WRKY superfamily of plant transcription factors. Trends Plant Sci 5: 199–206 10785665 10.1016/s1360-1385(00)01600-9

[koac032-B12] Fernandez-Pozo N , Rosli HG, Martin GB, Mueller LA (2015) The SGN VIGS tool: user-friendly software to design virus-induced gene silencing (VIGS) constructs for functional genomics. Mol Plant 8: 486–488 25667001 10.1016/j.molp.2014.11.024

[koac032-B13] Finley D (2009) Recognition and processing of ubiquitin-protein conjugates by the proteasome. Annu Rev Biochem 78: 477–513 19489727 10.1146/annurev.biochem.78.081507.101607 PMC3431160

[koac032-B14] Garner CM , Kim SH, Spears BJ, Gassmann W (2016) Express yourself: Transcriptional regulation of plant innate immunity. Sem Cell Devel Biol 56: 150–162 10.1016/j.semcdb.2016.05.002 27174437

[koac032-B15] Gimenez-Ibanez S , Boter M, Fernández-Barbero G, Chini A, Rathjen JP, Solano R (2014) The bacterial effector HopX1 targets JAZ transcriptional repressors to activate jasmonate signaling and promote infection in Arabidopsis. PLoS Biol 12: e1001792. 24558350 10.1371/journal.pbio.1001792 PMC3928049

[koac032-B16] Gimenez-Ibanez S , Boter M, Ortigosa A, García-Casado G, Chini A, Lewsey MG, Ecker JR, Ntoukakis V, Solano R (2017) JAZ2 controls stomata dynamics during bacterial invasion. New Phytol 213: 1378–1392 28005270 10.1111/nph.14354

[koac032-B17] Huh SU , Lee GJ, Jung JH, Kim Y, Kim YJ, Paek KH (2015) *Capsicum annuum* transcription factor WRKYa positively regulates defense response upon TMV infection and is a substrate of CaMK1 and CaMK2. Sci Rep 5: 7981 25613640 10.1038/srep07981 PMC5379037

[koac032-B18] Hurley B , Lee D, Mott A, Wilton M, Liu J, Liu YC, Angers S, Coaker G, Guttman DS, Desveaux D (2014) The *Pseudomonas syringae* type III effector HopF2 suppresses Arabidopsis stomatal immunity. PLoS ONE 9: e114921 25503437 10.1371/journal.pone.0114921 PMC4263708

[koac032-B19] Ishihama N , Yoshioka H (2012) Post-translational regulation of WRKY transcription factors in plant immunity. Curr Opin Plant Biol 15: 431–437 22425194 10.1016/j.pbi.2012.02.003

[koac032-B20] Ishihama N , Yamada R, Yoshioka M, Katou S, Yoshioka H (2011) Phosphorylation of the *Nicotiana benthamiana* WRKY8 transcription factor by MAPK functions in the defense response. Plant Cell 23: 1153–1170 21386030 10.1105/tpc.110.081794 PMC3082260

[koac032-B21] Karimi M , Inzé D, Depicker A (2002) GATEWAY^(TM)^ vectors for *Agrobacterium*-mediated plant transformation. Trends Plant Sci 7: 193–195 11992820 10.1016/s1360-1385(02)02251-3

[koac032-B22] Katsir L , Schilmiller AL, Staswick PE, He SY, Howe GA (2008) COI1 is a critical component of a receptor for jasmonate and the bacterial virulence factor coronatine. Proc Natl Acad Sci 105: 7100–7105 18458331 10.1073/pnas.0802332105 PMC2383947

[koac032-B23] Kulathu Y , Komander D (2012) Atypical ubiquitylation—the unexplored world of polyubiquitin beyond Lys48 and Lys63 linkages. Nat Rev Mol Cell Biol 13: 508–523 22820888 10.1038/nrm3394

[koac032-B24] Kumar S , Stecher G, Li M, Knyaz C, Tamura K (2018) MEGA X: molecular evolutionary genetics analysis across computing platforms. Mol Biol Evol 35: 1547–1549 29722887 10.1093/molbev/msy096 PMC5967553

[koac032-B25] Le Roux C , Huet G, Jauneau A, Camborde L, Trémousaygue D, Kraut A, Zhou B, Levaillant M, Adachi H, Yoshioka H, et al (2015) A receptor pair with an integrated decoy converts pathogen disabling of transcription factors to immunity. Cell 161: 1074–1088 26000483 10.1016/j.cell.2015.04.025

[koac032-B26] Lewis LA , Polanski K, de Torres-Zabala M, Jayaraman S, Bowden L, Moore J, Penfold CA, Jenkins DJ, Hill C, Baxter L, et al (2015) Transcriptional dynamics driving MAMP-triggered immunity and pathogen effector-mediated immunosuppression in Arabidopsis leaves following infection with *Pseudomonas syringae* pv *tomato* DC3000. Plant Cell 27: 3038–3064 26566919 10.1105/tpc.15.00471 PMC4682296

[koac032-B27] Li B , Meng X, Shan L, He P (2016) Transcriptional regulation of pattern-triggered immunity in plants. Cell Host Microbe 19: 641–650 27173932 10.1016/j.chom.2016.04.011 PMC5049704

[koac032-B28] Lichtenthaler HK (1987) Chlorophylls and carotenoids, pigments of photosynthetic biomembranes. Meth Enzymol 148: 350–382

[koac032-B29] Lin Y , Hu Q, Zhou J, Yin W, Yao D, Shao Y, Zhao Y, Guo B, Xia Y, Chen Q, et al (2021) *Phytophthora sojae* effector Avr1d functions as an E2 competitor and inhibits ubiquitination activity of GmPUB13 to facilitate infection. Proc Natl Acad Sci USA 118: e2018312118 33658365 10.1073/pnas.2018312118 PMC7958378

[koac032-B30] Liu B , Jiang Y, Tang H, Tong S, Lou S, Shao C, Zhang J, Song Y, Chen N, Bi H, et al (2021) The ubiquitin E3 ligase SR1 modulates the submergence response by degrading phosphorylated WRKY33 in Arabidopsis. Plant Cell 33: 1771–1789 33616649 10.1093/plcell/koab062 PMC8254483

[koac032-B31] Liu Y , Schiff M, Dinesh-Kumar SP (2002) Virus-induced gene silencing in tomato. Plant J 31: 777–786 12220268 10.1046/j.1365-313x.2002.01394.x

[koac032-B1001] **Lozano-Duran R, Bourdais G, He SY, Robatzek S** (2014) The bacterial effector HopM1 suppresses PAMP-triggered oxidative burst and stomatal immunity. New Phytol **202**: 259–269 10.1111/nph.12651 24372399

[koac032-B32] Mao G , Meng X, Liu Y, Zheng Z, Chen Z, Zhang S (2011) Phosphorylation of a WRKY transcription factor by two pathogen-responsive MAPKs drives phytoalexin biosynthesis in Arabidopsis. Plant Cell 23: 1639–1653 21498677 10.1105/tpc.111.084996 PMC3101563

[koac032-B33] Matsushita A , Inoue H, Goto S, Nakayama A, Sugano S, Hayashi N, Takatsuji H (2013) Nuclear ubiquitin proteasome degradation affects WRKY45 function in the rice defense program. Plant J 73: 302–313 23013464 10.1111/tpj.12035 PMC3558880

[koac032-B34] McGuire RG , Jones JB, Scott JW (1991) Epiphytic populations of *Xanthomonas campestris* pv. *vesicatoria* on tomato cultigens resistant and susceptible to bacterial spot. Plant Dis 75: 606–609

[koac032-B35] Melotto M , Underwood W, He SY (2008) Role of stomata in plant innate immunity and foliar bacterial diseases. Annu Rev Phytopathol 46: 101–122 18422426 10.1146/annurev.phyto.121107.104959 PMC2613263

[koac032-B36] Melotto M , Underwood W, Koczan J, Nomura K, He SY (2006) Plant stomata function in innate immunity against bacterial invasion. Cell 126: 969–980 16959575 10.1016/j.cell.2006.06.054

[koac032-B37] Miao Y , Zentgraf U (2010) A HECT E3 ubiquitin ligase negatively regulates Arabidopsis leaf senescence through degradation of the transcription factor WRKY53. Plant J 63: 179–188 20409006 10.1111/j.1365-313X.2010.04233.x

[koac032-B38] Mittal S , Davis KR (1995) Role of the phytotoxin coronatine in the infection of Arabidopsis thaliana by *Pseudomonas syringae* pv. *tomato*. Mol Plant Microbe Interact 8: 165–171 7539639 10.1094/mpmi-8-0165

[koac032-B39] Moore JW , Loake GJ, Spoel SH (2011) Transcription dynamics in plant immunity. Plant Cell 23: 2809–2820 21841124 10.1105/tpc.111.087346 PMC3180793

[koac032-B40] Mukhopadhyay D , Riezman H (2007) Proteasome-independent functions of ubiquitin in endocytosis and signaling. Science 315: 201–205 17218518 10.1126/science.1127085

[koac032-B41] Nakamura S , Mano S, Tanaka Y, Ohnishi M, Nakamori C, Araki M, Niwa T, Nishimura M, Kaminaka H, Nakagawa T, et al (2010) Gateway binary vectors with the bialaphos resistance gene, bar, as a selection marker for plant transformation. Biosci Biotechnol Biochem 74: 1315–1319 20530878 10.1271/bbb.100184

[koac032-B42] Nietzsche M , Guerra T, Alseekh S, Wiermer M, Sonnewald S, Fernie AR, Börnke F (2018) STOREKEEPER RELATED1/G-element binding protein (STKR1) interacts with protein kinase SnRK1. Plant Physiol 176: 1773–1792 29192025 10.1104/pp.17.01461 PMC5813543

[koac032-B43] Oh SK , Baek KH, Park JM, Yi SY, Yu SH, Kamoun S, Choi D (2008) *Capsicum annuum* WRKY protein *Ca*WRKY1 is a negative regulator of pathogen defense. New Phytol 177: 977–989 18179600 10.1111/j.1469-8137.2007.02310.x

[koac032-B44] Okada M , Ito S, Matsubara A, Iwakura I, Egoshi S, Ueda M (2009) Total syntheses of coronatines by exo-selective Diels–Alder reaction and their biological activities on stomatal opening. Organ Biomol Chem 7: 3065–3073

[koac032-B45] Osdaghi E , Jones JB, Sharma A, Goss EM, Abrahamian P, Newberry EA, Potnis N, Carvalho R, Choudhary M, Paret ML, et al (2021) A centenary for bacterial spot of tomato and pepper. Mol Plant Pathol 22: 1500–1519 10.1111/mpp.13125 34472193 PMC8578828

[koac032-B46] Pandey SP , Somssich IE (2009) The role of WRKY transcription factors in plant immunity. Plant Physiol 150: 1648–1655 19420325 10.1104/pp.109.138990 PMC2719123

[koac032-B47] Pandey SP , Roccaro M, Schon M, Logemann E, Somssich IE (2010) Transcriptional reprogramming regulated by WRKY18 and WRKY40 facilitates powdery mildew infection of Arabidopsis. Plant J 64: 912–923 21143673 10.1111/j.1365-313X.2010.04387.x

[koac032-B48] Popov G , Fraiture M, Brunner F, Sessa G.(2016) Multiple *Xanthomonas euvesicatoria* type III effectors inhibit flg22-triggered immunity. Mol Plant Microbe Interact 29: 651–660 27529660 10.1094/MPMI-07-16-0137-R

[koac032-B49] Potnis N , Krasileva K, Chow V, Almeida NF, Patil PB, Ryan RP, Sharlach M, Behlau F, Dow JM, Momol MT, et al (2011) Comparative genomics reveals diversity among xanthomonads infecting tomato and pepper. BMC Genom 12: 146 10.1186/1471-2164-12-146 PMC3071791 21396108

[koac032-B50] Ramos LJ , Volin RB (1987) Role of stomatal opening and frequency on infection of *Lycopersicon* spp. by *Xanthomonas campestris* pv. vesicatoria. Phytopathology 77: 1311–1317

[koac032-B51] Rosli HG , Zheng Y, Pombo MA, Zhong S, Bombarely A, Fei Z, Collmer A, Martin GB (2013) Transcriptomics-based screen for genes induced by flagellin and repressed by pathogen effectors identifies a cell wall-associated kinase involved in plant immunity. Genome Biol 14: R139 24359686 10.1186/gb-2013-14-12-r139 PMC4053735

[koac032-B52] Sawinski K , Mersmann S, Robatzek S, Böhmer M (2013) Guarding the green: Pathways to stomatal immunity. Mol Plant Microbe Interact 26: 626–632 23441577 10.1094/MPMI-12-12-0288-CR

[koac032-B53] Schulze S , Kay S, Büttner D, Egler M, Eschen-Lippold L, Hause G, Krüger A, Lee J, Müller O, Scheel D, et al (2012) Analysis of new type III effectors from Xanthomonas uncovers XopB and XopS as suppressors of plant immunity. New Phytol 195: 894–911 22738163 10.1111/j.1469-8137.2012.04210.x

[koac032-B54] Schwartz AR , Potnis N, Timilsina S, Wilson M, Patané J, Martins J, Minsavage GV, Dahlbeck D, Akhunova A, Almeida N, et al (2015) Phylogenomics of *Xanthomonas* field strains infecting pepper and tomato reveals diversity in effector repertoires and identifies determinants of host specificity. Front Microbiol 6: 535 26089818 10.3389/fmicb.2015.00535 PMC4452888

[koac032-B55] Shen L , Yang S, Yang T, Liang J, Cheng W, Wen J, Liu Y, Li J, Shi L, Tang Q, et al (2016) CaCDPK15 positively regulates pepper responses to *Ralstonia solanacearum* inoculation and forms a positive-feedback loop with CaWRKY40 to amplify defense signaling. Sci Rep 6: 22439 26928570 10.1038/srep22439 PMC4772545

[koac032-B56] Staswick PE , Tiryaki I (2004) The oxylipin signal jasmonic acid is activated by an enzyme that conjugates it to isoleucine in Arabidopsis. Plant Cell 16: 2117–2127 15258265 10.1105/tpc.104.023549 PMC519202

[koac032-B57] Teper D , Burstein D, Salomon D, Gershovitz M, Pupko T, Sessa G (2016) Identification of novel *Xanthomonas euvesicatoria* type III effector proteins by a machine-learning approach. Mol Plant Pathol 17: 398–411 26104875 10.1111/mpp.12288 PMC6638362

[koac032-B58] Tsuda K , Somssich IE (2015) Transcriptional networks in plant immunity. New Phytol 206: 932–947 25623163 10.1111/nph.13286

[koac032-B59] Üstün S , Börnke F (2017) Ubiquitin proteasome activity measurement in total plant extracts. Bio-protocol 7: e2532 34541188 10.21769/BioProtoc.2532 PMC8413642

[koac032-B60] Üstün S , Bartetzko V, Börnke F (2013) The *Xanthomonas campestris* type III effector XopJ targets the host cell proteasome to suppress salicylic-acid mediated plant defence. PLoS Pathog 9: e1003427 23785289 10.1371/journal.ppat.1003427 PMC3681735

[koac032-B61] Üstün S , König P, Guttman DS, Börnke F (2014) HopZ4 from *Pseudomonas syringae*, a member of the HopZ type III effector family from the YopJ superfamily, inhibits the proteasome in plants. Mol Plant Microb Interact 27: 611–623 10.1094/MPMI-12-13-0363-R 24625030

[koac032-B62] Üstün S , Sheikh A, Gimenez-Ibanez S, Jones A, Ntoukakis V, Börnke F (2016) The proteasome acts as a hub for plant immunity and is targeted by *Pseudomonas* type III effectors. Plant Physiol 172: 1941–1958 27613851 10.1104/pp.16.00808 PMC5100764

[koac032-B63] Vierstra RD (2009) The ubiquitin-26S proteasome system at the nexus of plant biology. Nat Rev Mol Cell Biol 10: 385–397 19424292 10.1038/nrm2688

[koac032-B64] Xu X , Chen C, Fan B, Chen Z (2006) Physical and functional interactions between pathogen-induced *Arabidopsis* WRKY18, WRKY40, and WRKY60 transcription factors. Plant Cell 18: 1310–1326 16603654 10.1105/tpc.105.037523 PMC1456877

[koac032-B65] Ye Q , Wang H, Su T, Wu WH, Chen YF (2018) The ubiquitin E3 ligase PRU1 regulates WRKY6 degradation to modulate phosphate homeostasis in response to low-Pi stress in *Arabidopsis*. Plant Cell 30: 1062–1076 29567663 10.1105/tpc.17.00845 PMC6002188

[koac032-B66] Ye R , Yao QH, Xu ZH, Xue HW (2004) Development of an efficient method for the isolation of factors involved in gene transcription during rice embryo development. Plant J 38: 348–357 15078336 10.1111/j.1365-313X.2004.02037.x

[koac032-B67] Zheng XY , Spivey NW, Zeng W, Liu PP, Fu ZQ, Klessig DF, He SY, Dong X (2012) Coronatine promotes *Pseudomonas syringae* virulence in plants by activating a signaling cascade that inhibits salicylic acid accumulation. Cell Host Microbe 11: 587–596 22704619 10.1016/j.chom.2012.04.014 PMC3404825

