# Supplementary material for: The Xanthomonas type-III effector XopS stabilizes CaWRKY40a to regulate defense responses and stomatal immunity in pepper (Capsicum annuum)
Source: Plant Cell. 2022 Feb 2;34(5):1684–708. doi: 10.1093/plcell/koac032 (PMC9048924; doi:10.1093/plcell/koac032)
Supplement: koac032_supplementary_data [file koac032_supplementary_data.zip › tpc.21.00991_Supplemental Figures and Tables.pdf]

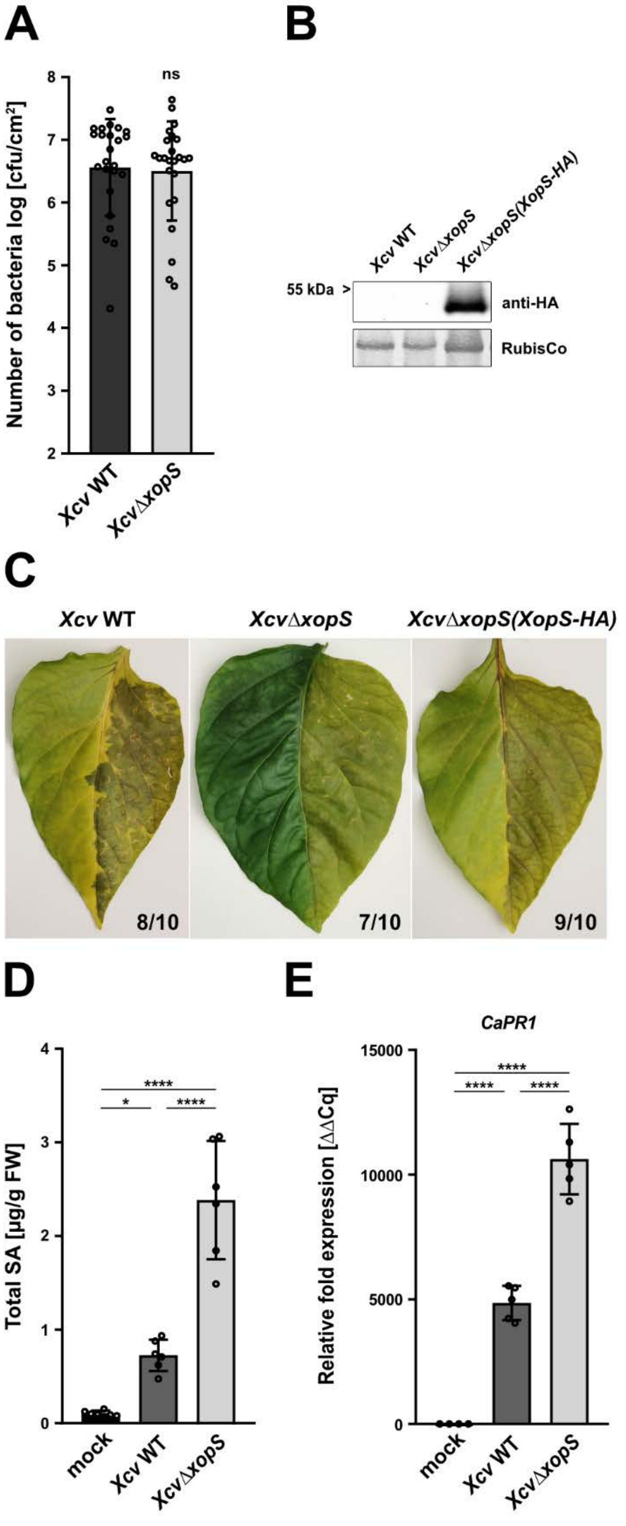
A
B
Number of bacteria log [cfu/cm²]
ns
Xcv WT
XcvΔxopS
XcvΔxopS(XopS-HA)
55 kDa >
anti-HA
RubisCo
C
8/10
7/10
9/10
D
E
CaPR1
Total SA [µg/g FW]
Relative fold expression [ΔΔCq]
mock

Supplementary Figure S1. XopS contributes to *Xcv* symptom development on susceptible pepper plants and alters SA responses. (Supports Figure 1). **(A)** Deletion of *xopS* does not affect bacterial multiplication *in planta*. Leaves of susceptible pepper plants were syringe-inoculated with *Xcv* wild-type (WT) or the *Xcv*Δ*xopS* strain at a bacterial density of OD_{600} = 0.0001 and colony forming units (CFU) in infected tissue were quantified at 5 days post inoculation (dpi). Bars represent the mean of n = 12 biological replicates (and 2 technical replicates per biological replicate) ± SD. ns, not significant according to Student's *t*-test. The experiment was carried out twice with similar results. **(B)** Verification of XopS-HA protein expression in the *Xcv*Δ*xopS*(*XopS-HA*) complementation strain. Leaves of susceptible pepper plants were syringe-inoculated with *Xcv* WT*, Xcv*Δ*xopS,* or the complementation strain *Xcv*Δ*xopS*(*XopS-HA*) at OD_{600} = 0.1. Total protein extracts from pepper leaves were prepared at 3 dpi and equal volumes of each extract representing approximately equal protein amounts were immunoblotted. Proteins were detected using an anti-HA (hemagglutinin) antibody and Amido black staining of RubisCo served as loading control. **(C)** Disease symptom development in pepper leaves infected with *Xcv* WT*, Xcv*Δ*xopS,* or *Xcv*Δ*xopS*(*XopS-HA*). Indicated strains were syringe-inoculated with an OD_{600} of 0.2 into susceptible leaves of pepper plants and the disease phenotype of one representative leaf out of 10 infected leaves is shown. Pictures were taken at 4 dpi. Numbers at the bottom indicate the frequency of occurrence of the phenotype. **(D)** Salicylic acid (SA) content changes upon infection with different *Xcv* strains in pepper plants. Susceptible pepper leaves were syringe-inoculated with $MgCl_2$ (mock) or a bacterial suspension of *Xcv* WT and *Xcv*Δ*xopS* at OD_{600} = 0.2, respectively. Total SA (free SA+ glycosylated SA) levels in infected tissue were measured 2 dpi and compared to total SA levels in mock treated plants. Bars represent the mean of n = 6 biological replicates ± SD and asterisks (*, P < 0.05; ****, P < 0.0001) mark significant differences according to one-way ANOVA. FW, Fresh weight. The experiment was carried out twice with similar results. **(E)** Gene expression analysis of *CaPR1* upon *Xcv* WT and *Xcv*Δ*xopS* infection. Leaves of susceptible pepper plants were syringe-inoculated with indicated strains at OD_{600} = 0.2. Samples were taken 10 hours post inoculation (hpi), the mRNA level of *CaPR1* was measured by RT-qPCR and compared to mock treated leaves. *TUBULIN* was used as a reference gene. Each bar represents the mean of at least n = 4 biological replicates ± SD (n = 4 for mock, n = 5 for *Xcv* WT and *Xcv*Δ*xopS*). Asterisks (****, P < 0.0001) mark significant differences according to one-way ANOVA. The experiment was carried out at least three times with similar results.

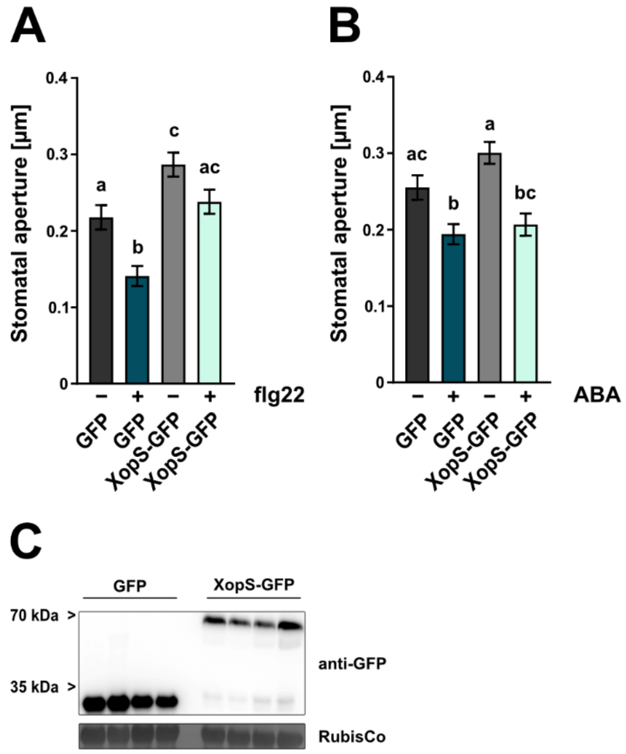

Supplementary Figure S2. Transient expression of XopS in *N. benthamiana* inhibits stomatal closure in response to flg22. (Supports Figure 1). **(A)** Stomatal aperture measurement in *N. benthamiana* plants transiently expressing either free GFP (GFP) or XopS-GFP. Leaf discs were floated on water (control) or on water supplemented with 25 µM flg22 for 2 hours prior to the measurement of stomatal aperture under a microscope. Approximately 100 apertures from n = 4 independent plants were measured per individual treatment and are represented as width/length ratio. Bars represent the mean ± SE. Letters above bars represent the statistical significance determined by one-way ANOVA ($P < 0.05$). The experiment was carried out twice with similar results. **(B)** Stomatal aperture measurement in *N. benthamiana* plants transiently expressing either GFP or XopS-GFP and treated with abscisic acid (ABA). Leaf discs were floated on water (control) or on water supplemented with 50 µM ABA for 2 hours prior to the measurement of stomatal aperture under a microscope. Approximately 100 apertures from n = 4 independent plants were measured per individual treatment and are represented as width/length ratio. Bars represent the mean ± SE. Letters above bars represent the statistical significance determined by one-way ANOVA ($P < 0.05$). The experiment was carried out twice with similar results. **(C)** Expression of GFP and XopS-GFP in *Agrobacterium* infiltrated *N. benthamiana* leaves. Total protein extracts were generated 24 hpi and proteins were detected by immunoblotting using an anti-GFP antibody. Amido black staining of RubisCo served as loading control. The four biological replicates used for the analysis of stomatal aperture are shown.

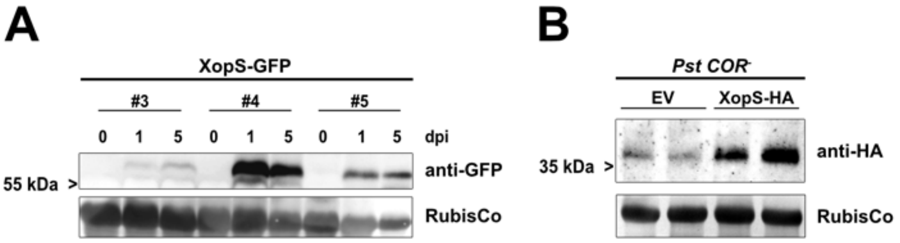

Supplementary Figure S3. Verification of ectopic XopS protein expression. (Supports Figure 2). **(A)** Western blot analysis to confirm XopS-GFP protein expression in β-estradiol inducible transgenic *Arabidopsis* lines. Four-week-old plants grown in soil under short day conditions (8 h light/16 h dark) were sprayed with 50 µM β-estradiol and leaf samples were taken at indicated time points (dpi = days post induction). Expression of the XopS-GFP fusion protein was detected by immunoblotting with an anti-GFP antibody. Amido black staining of RubisCo served as loading control. **(B)** Verification of XopS-HA protein expression in the *Pst COR*$^{-}$ (*XopS-HA*) complementation strain. Leaves of *N. benthamiana roq1* plants were syringe-inoculated with *Pst COR*$^{-}$ complemented with empty vector (EV) or the complementation strain *Pst COR*$^{-}$ (*XopS-HA*) at OD_{600} = 0.2. Total protein extracts from *N. benthamiana roq1* leaves were prepared at 3 dpi and equal volumes of each extract representing approximately equal protein amounts were immunoblotted. Proteins were detected using an anti-HA antibody and Amido black staining of RubisCo served as loading control.

A

	1	2	3	4	5	6	7	8	9
**1. *Nt*WRKY40**		50.4	100.0	72.3	59.9	61.9	38.8	17.8	20.3
**2. *At*WRKY40**	64.9		50.4	48.1	43.7	46.0	42.4	16.1	20.3
**3. *Nb*WRKY40**	100.0	64.9		72.3	59.9	61.9	38.8	17.8	20.3
**4. *Ca*WRKY40a**	82.2	61.4	82.2		61.4	62.7	38.2	20.6	18.9
**5. *Ca*WRKY40**	72.6	59.0	72.6	75.9		81.6	35.8	19.0	19.4
**6. *Nb*WRKY40a**	74.7	61.8	74.7	79.2	87.3		38.0	19.4	20.2
**7. *Nb*WRKY40e**	52.8	57.0	52.8	52.2	50.4	52.8		16.1	22.0
**8. *Nb*WRKY8**	31.4	29.6	31.4	34.2	33.8	32.9	27.7		16.7
**9. *Ca*WRKY1**	30.4	34.8	30.4	28.1	28.3	30.3	37.3	23.4	

B

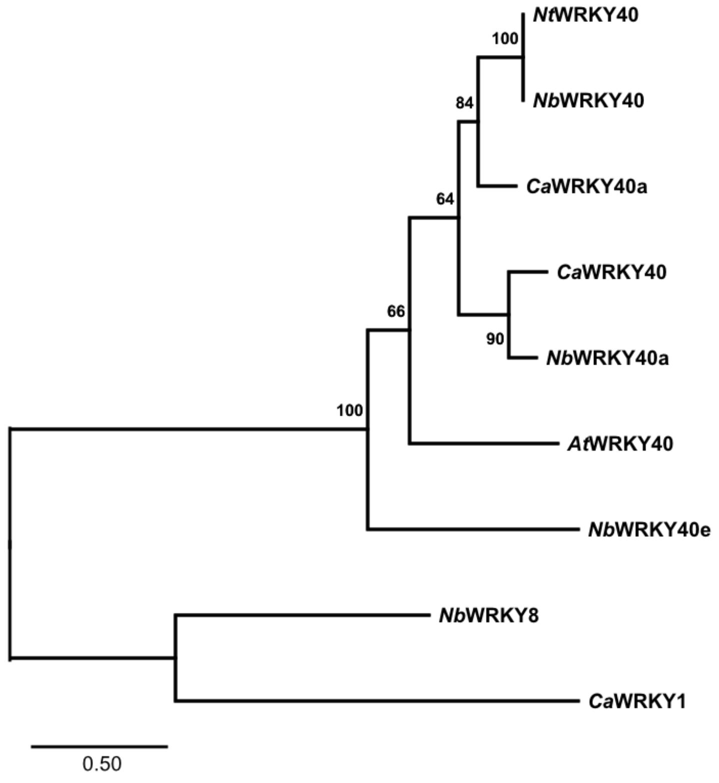

Supplemental Figure S4. Sequence relationship of different WRKY proteins. (Supports Figure 3). **(A)** Identity/Similarity matrix of WRKY protein sequence relationship. Blue: percentage of amino acid identity. Green: percentage of amino acid similarity. **(B)** Phylogenetic tree of WRKY proteins constructed using MEGA software (version X) with the neighbor-joining algorithm. Bootstrap values indicate the confidence of each branch. The scale bar indicates the branch length. *Arabidopsis thaliana* (*At*WRKY40; AT1G80840), *Capsicum annuum* (*Ca*WRKY40, XP_016578457; *Ca*WRKY40a, XP_016562883; *Ca*WRKY1, XP_016582129.1), *Nicotiana tabacum* (*Nt*WRKY40, XM_016624265) and *Nicotiana benthamiana* (*Nb*WRKY40, Niben101Scf06091g04005.1; *Nb*WRKY40a, Niben101Ctg16115g00003.1; *Nb*WRKY40e, Niben101Scf04944g05002.1; *Nb*WRKY8, Niben101Scf02362g02014.1)

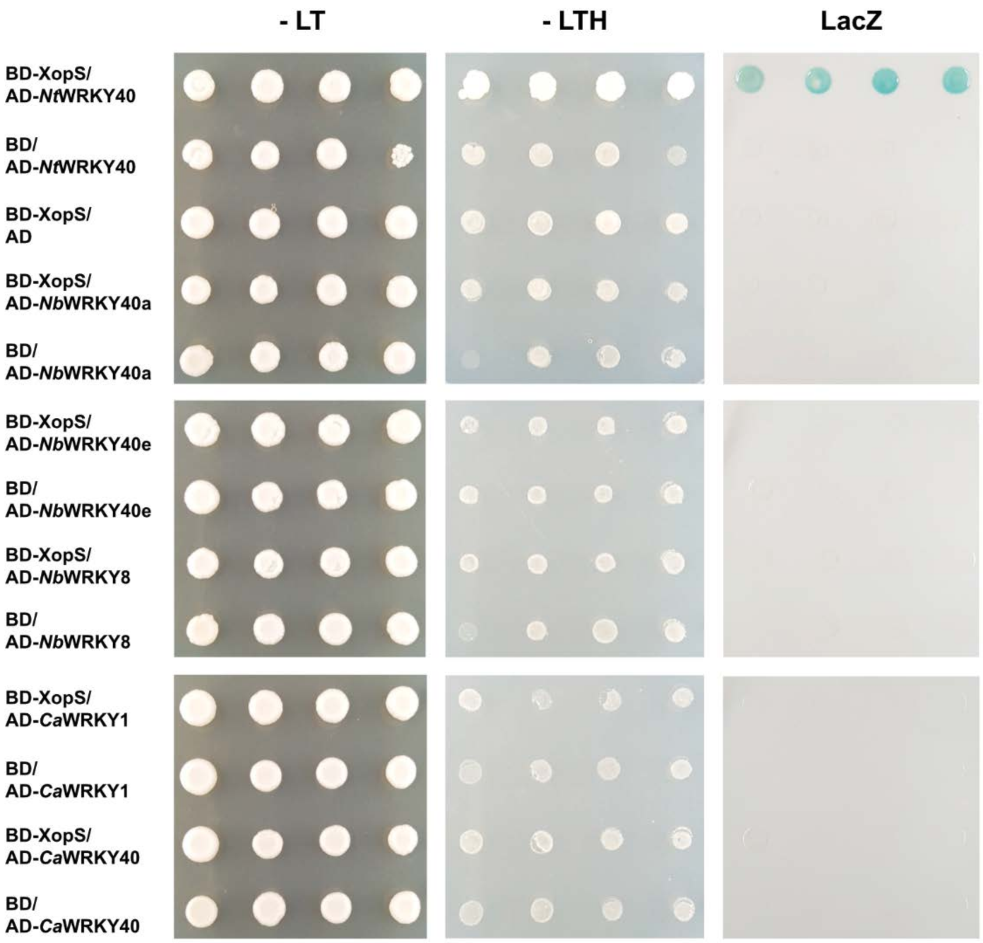

Supplementary Figure S5. XopS does not interact with WRKY proteins other than WRKY40 in yeast. (Supports Figure 3). Proteins were either fused to the GAL4 DNA binding domain (BD) or to the GAL4 activation domain (AD). Indicated combinations were transformed into yeast strain Y190. Four independent transformants were grown on selective media before a LacZ filter assay was performed. BD-XopS/AD-*Nt*WRKY40 served as positive control, while empty pGAD424 (AD) or pGBT9 (BD) vector served as negative control. *Nt*WRKY40, *Nicotiana tabacum* WRKY40; *Nb*WRKY40a, *Nicotiana benthamiana* WRKY40a; *Nb*WRKY40e, *Nicotiana benthamiana* WRKY40e; *Nb*WRKY8, *Nicotiana benthamiana* WRKY8; *Ca*WRKY1, *Capsicum annuum* WRKY1; *Ca*WRKY40, *Capsicum annuum* WRKY40. – LT, yeast growth on medium without Leu and Trp. – LTH, yeast growth on medium lacking Leu, Trp and His indicating expression of the *HIS3* reporter gene. LacZ, activity of the *lacZ* reporter gene. The experiment was carried out three times with similar results.

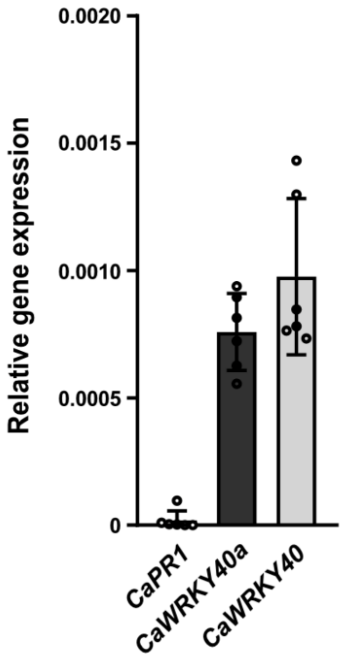

Supplementary Figure S6. Basal gene expression of pepper *WRKY40a* and *WRKY40*. (Supports Figure 5). Total RNA was extracted from untreated leaves of four-week-old pepper (ECW) plants and relative transcript levels of the SA responsive gene *CaPR1, CaWRKY40a* and *CaWRKY40* were determined by RT-qPCR. *UBIQUITIN-CONJUGATING PROTEIN 3* (*UBI*-3) was used as a reference gene. Bars represent the mean of n = 6 biological replicates ± SD. The experiment was carried out twice with similar results.

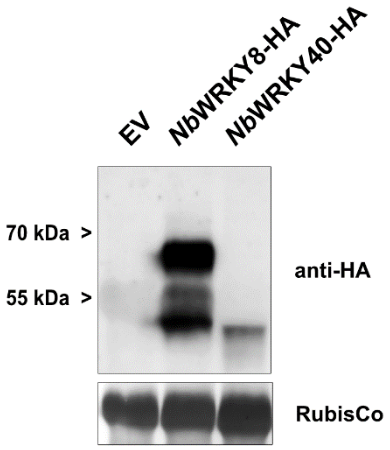

Supplementary Figure S7. Verification of effector protein expression in *W-box::GUS* reporter gene analyses. (Supports Figure 7). Total protein extracts from *Agrobacterium*-infiltrated leaves were prepared 48 hpi and protein expression of indicated proteins was detected by immunoblotting using an anti-HA antibody. Amido black staining of RubisCo served as loading control. Leaf discs from the biological replicates used for *W-box:GUS* reporter gene analyses were pooled for the immunoblot shown here.

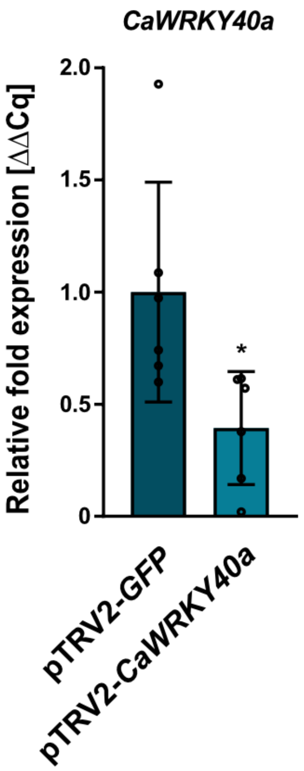

Supplementary Figure S8. Verification of *CaWRKY40a* down-regulation in virus-induced gene silencing (VIGS) pepper plants used for defense gene expression analyses. (Supports Figure 7). Three weeks after infiltrating pepper plants with the silencing constructs, total RNA was isolated from excised leaves treated with 5 mM SA for 4 hours. The mRNA level of *CaWRKY40a* in pTRV2-*CaWRK40a* (*CaWRKY40a* silencing) plants was measured by RT-qPCR and compared to *CaWRKY40a* expression in pTRV2-*GFP* (*GFP* silencing, control) plants. *UBIQUITIN-CONJUGATING PROTEIN 3* (*UBI*-3) was used as a reference gene. Bars represent the mean of n = 6 biological replicates ± SD. Asterisks (*, P < 0.05) mark significant differences according to Student's *t*-test.

A

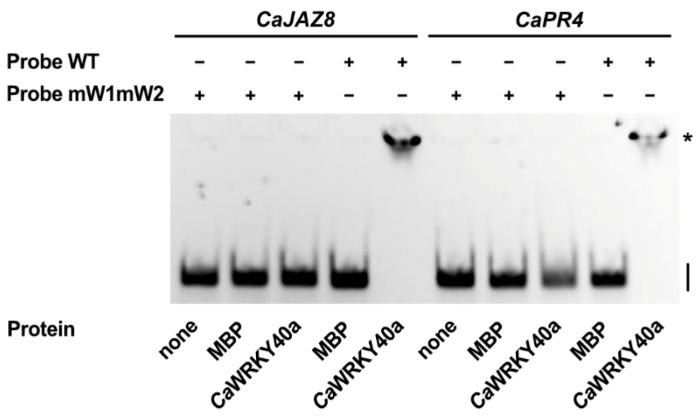

B

***CaJAZ8* w-box promoter fragment**

cgtaattgcccttgacaatggtcactgatttgttttgattgataaccaccgtgatttgtttagattga
taaccaccgttaataatagaaccaaaatcactatttttaattaattaaaaattaaatat**gaaatgtca**
aatatcacagactac

mutated *CaJAZ8* w-box promoter fragment

cgtaattgcccGAGAGA**at**AGAGactgatttgttttgattgataaccaccgtgatttgtttagattga
taaccaccgttaataatagaaccaaaatcactatttttaattaattaaaaattaaatat**gaaaGAGAG**
Aatatcacagactac

***CaPR4* w-box promoter fragment**

agagatccaattagataaatgtcccattcataatgtttggtcaaatgttagtcctctttaatttccca
aacgaaatagcctcttagcttgtttatctacacgtactggagaagactattcttcag**cataaatggtc**
aaattaagcaacag

mutated *CaPR4* w-box promoter fragment

agagatccaattagataaatgtcccattcataatgtttGAGAGAatgttagtcctctttaatttccca
aacgaaatagcctcttagcttgtttatctacacgtactggagaagactattcttcag**cataaatGAGA**
GAattaagcaacag

Supplementary Figure S9. Mutation of W-boxes abolishes binding of *Ca*WRKY40a to promotor fragments of *CaJAZ8* and *CaPR4*. (Supports Figure 7). **(A)** Lanes 5 and 10: EMSAs where performed with a Cy5-labelled 150 bp promoter fragment of *CaJAZ8* and *CaPR4* carrying two W-boxes (Probe WT) and recombinant MBP-*Ca*WRKY40a. Lanes 4 and 9: MBP protein was included as a negative control. Lanes 3 and 8: EMSAs were performed with a mutant version (Probe mW1mW2) of the same probes lacking the predicted W-boxes. Lanes 2 and 7: MBP protein was included as a negative control. Lanes 1 and 6 show additional negative controls were either a mutant *CaJAZ8* or a *CaPR4* probe was incubated without addition of a recombinant protein. Protein-DNA complexes were separated from unbound probe on a 5% TBE gel. On the right-hand side of the gel, specific retarded protein-DNA complexes are marked by an asterisk, whereas free running probes are designated by a black bar. The experiment was carried out three times with similar results. **(B)** Fragments used for EMSAs. Predicted W-boxes are shaded in red. Mutated motifs are green. Location of primers used for PCR amplification is indicated by bold letters.

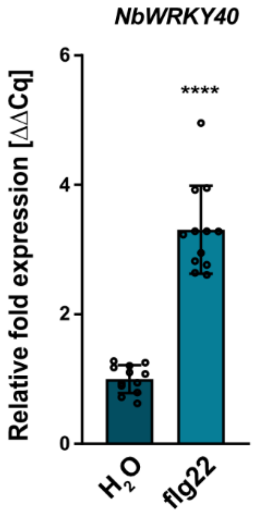

Supplementary Figure S10. Induction of *NbWRKY40* expression upon flg22 treatment. (Supports Figure 9). Gene expression analysis of *NbWRKY40* upon flg22 treatment. *N. benthamiana* leaf discs were floated on water (control) or on water supplemented with 25 µM flg22 for 2 hours, followed by extraction of total RNA. The mRNA level of *NbWRKY40* in flg22 treated samples was measured by RT-qPCR and compared to *NbWRKY40* expression in H_2O treated leaf discs. *ACTIN* was used as a reference gene. Each bar represents the mean of n = 12 biological replicates ± SD. Asterisks (****, P < 0.0001) mark significant differences according to Student's *t*-test. The experiment was carried out twice with similar results.

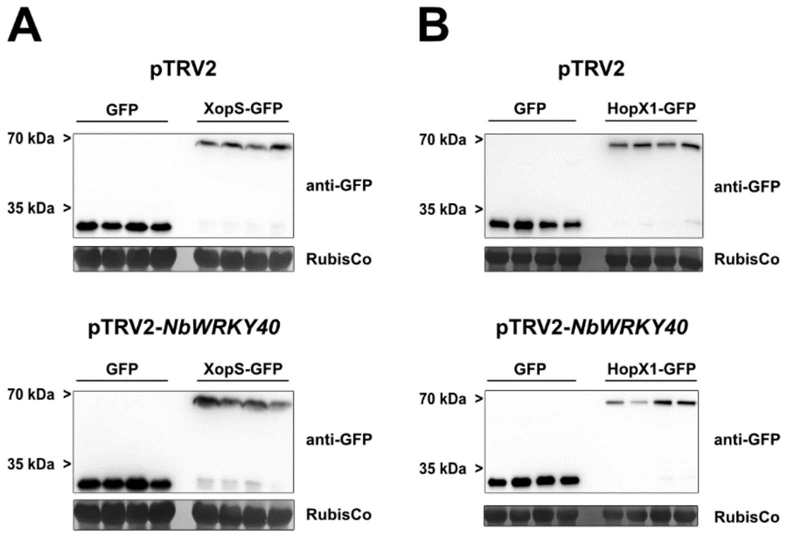

Supplementary Figure S11. Verification of protein expression in pTRV2 (empty vector silencing; control) and pTRV2-*NbWRKY40* (*NbWRKY40* silencing) VIGS *N. benthamiana* plants transiently expressing either GFP, XopS-GFP or HopX1-GFP. (Supports Figure 9). **(A)** and **(B)** Total protein extracts from *Agrobacterium*-infiltrated leaves were prepared 24 hpi and protein expression of indicated proteins was detected by immunoblotting using an anti-GFP antibody. Amido black staining of RubisCo served as loading control. The four biological replicates used for the analysis of stomatal aperture in the respective experiment are shown.

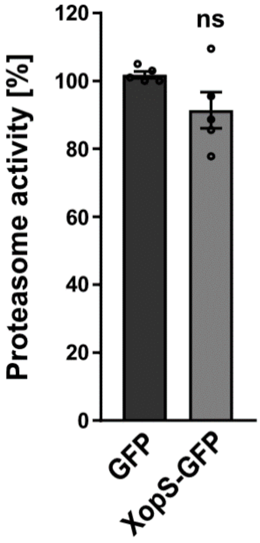

Supplementary Figure S12. Expression of XopS does not affect proteasome activity. (Supports Figure 10). Proteasome activity is shown in *N. benthamiana* leaves following transient expression of XopS or GFP control. Relative proteasome activity in total protein extracts was determined by monitoring the breakdown of the fluorogenic peptide Suc-LLVY-AMC at 30˚C in a fluorescence spectrophotometer. The GFP control was set to 100%. Bars represent the mean of n = 5 biological replicates ± SE. ns, not significant according to Student's *t*-test. The experiment was carried out at least twice with similar results.

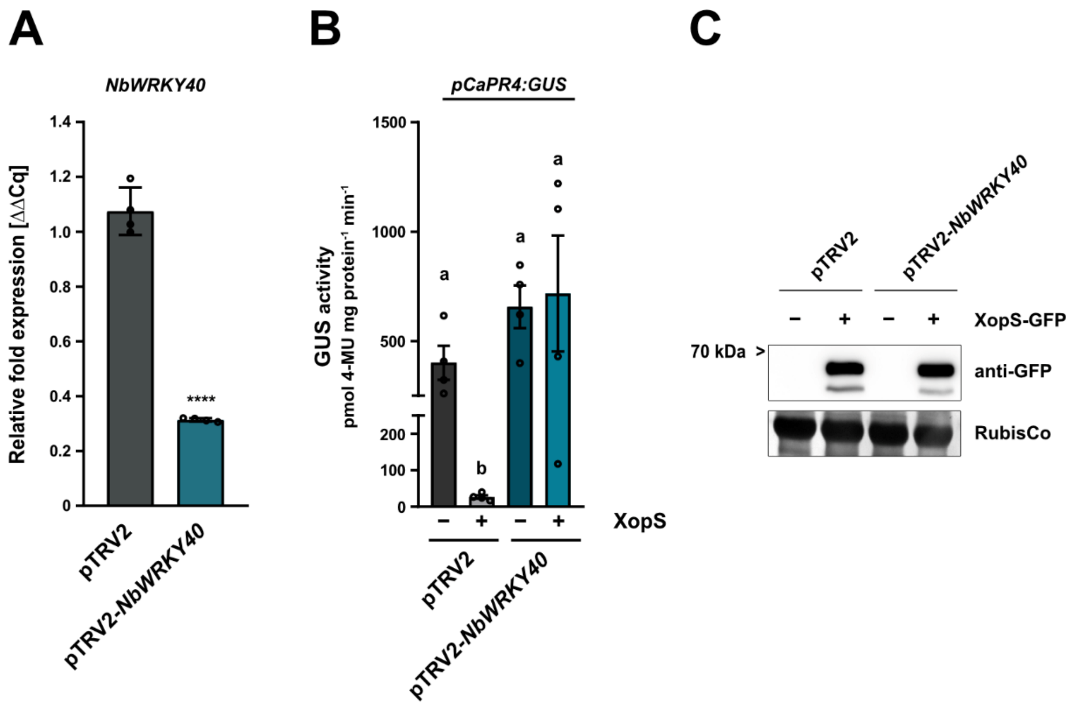

Supplementary Figure S13. The ability of XopS to repress *pCaPR4:GUS* reporter gene expression depends on WRKY40. (Supports Figure 11). **(A)** Verification of *NbWRKY40* down-regulation in VIGS *N. benthamiana* pTRV2-*NbWRKY40* compared to pTRV2 control plants. Two weeks after infiltrating *N. benthamiana* plants with the silencing constructs, total RNA was isolated from excised leaves treated with 5 mM SA for 4 hours. The mRNA level of *NbWRKY40* in pTRV2-*NbWRKY40* was measured by RT-qPCR and compared to *NbWRKY40* expression in pTRV2 control plants. *ACTIN* was used as a reference gene. Bars represent the mean of n = 4 biological replicates ± SD and asterisks (****, $P < 0.0001$) mark significant differences according to Student's *t*-test. **(B)** Transactivation of the *pCaPR4*::GUS reporter gene in pTRV2 or pTRV2-*NbWRKY40* VIGS *N. benthamiana* plants either in presence or absence of XopS-GFP (XopS). Samples were taken 48 hours after *Agrobacterium*-infiltration of XopS and GUS activity is expressed in pmol 4-Methylumbelliferone mg protein^{-1} min^{-1}. Bars represent the mean of n = 4 biological replicates ± SD. Letters above bars represent the statistical significance determined by one-way ANOVA ($P < 0.05$). The experiment was carried out twice with similar results. **(C)** Verification of effector protein expression in *pCaPR4:GUS* reporter gene analyses. Total protein extracts from *Agrobacterium*-infiltrated leaves were prepared 24 hpi and XopS-GFP protein expression was detected by immunoblotting using an anti-GFP antibody. Amido black staining of RubisCo served as loading control. Leaf discs from the four biological replicates used for *pCaPR4:GUS* reporter gene analyses were pooled and used for the immunoblot analysis shown here.

Supplementary Table S1. Candidate XopS interaction partners identified by screening a *Nicotiana tabacum* yeast two-hybrid cDNA library using XopS as a bait.

**Number**	**Acc. No.**	**Annotation**
1	XP_016457007.1	probable WRKY transcription factor 40 [*Nicotiana tabacum*]
2	XP_009606047.1	40S ribosomal protein S5-like [*Nicotiana tomentosiformis*]
3	XP_009760231.1	probable BOI-related E3 ubiquitin-protein ligase 2 [*Nicotiana sylvestris*]
4	XP_009608860.1	betaine aldehyde dehydrogenase, chloroplastic-like [*Nicotiana tomentosiformis*]

Supplementary Table S2. Nucleotide sequences of gene-specific primers.		
**Target gene**		**Primer sequences used for qRT-PCR**
AtUBC9	Fw	5'-TGGATCGTGGGATTTTGGAAATGGC-3'
	Rv	5'-GCAACGGGTCCTGCGCTACAT-3'
AtJAZ10	Fw	5'-GAGAAGCGCAAGGAGAGATTAG-3'
	Rv	5'-CTTAGTAGGTAACGTAATCTCC-3'
AtMYC2	Fw	5'- GATGAGGAGGTGACGGATACGGAA-3'
	Rv	5'-CGCTTTACCAGCTAATCCCGCA-3'
NbACTIN	Fw	5'-GCCAACAGAGAGAAGATGACCCAGA-3'
	Rv	5'-ACACCATCACCAGAGTCCAACACAAT-3'
NbWRKY40	Fw	5'-GTGGCAACATATGAAGGAGAACA-3
	Rv	5'-CCCCAAGATTACTCTTCCCG-3'
NbWRKY40a	Fw	5'-CCCAACAACAACAACAACAAC-3'
	Rv	5'-CCTCATAGAAACGACAGAAACC-3'
NbWRKY40e	Fw	5'-TCCGGGAATTTTTTTGAATATGA-3'
	Rv	5'-CAGTGAAAAAACAAAACCGTTTGA-3'
CaACTIN	Fw	5'-GCCAACAGAGAGAAGATGACCCAGA-3'
	Rv	5'-ACACCATCACCAGAGTCCAACACAAT-3'
CaUBI-3	Fw	5'-TGTCCATCTGCTCTCTGTTG-3'
	Rv	5'-CACCCCAAGCACAATAAGAC-3
CaTUB	Fw	5'-GAGGGTGAGTGAGCAGTTC-3'
	Rv	5'-CTTCATCGTCATCTGCTGTC-3
CaWRKY40a	Fw	5'-GCCGATTAAACCCGAAAAAT-3'
	Rv	5'-ATGTCCTCCTGGTGATTTGC-3'
CaWRKY40	Fw	5'-AACTTGGATGTTGTGCCTGGA-3'
	Rv	5'-CTGTAACCTTGGCTTTTATGTGC-3'
CaPR1	Fw	5'-GCCGTGAAGATGTGGGTCAATGA-3'
	Rv	5'-TGAGTTACGCCAGACTACCTGAGTA-3'
CaPR4	Fw	5'-GAACACAAGCAACGGTGAGA-3'
	Rv	5'-GGCACTTGTTTAGGCAGAGC-3'
CaJAZ8	Fw	5'-GCCTTATGCCTCCTCCTCTT-3'
	Rv	5'-AGGCTCTGATATTGGCGATG-3'
CaCDPK15	Fw	5'-TTTTCTTTTCGCCCTTTA-3'
	Rv	5'-AATGAACTCCATCCAGCA-3'
		**Primer sequences used for EMSA**
pCaPR4	Fw	5'-AGAGATCCAATTAGATAAATGTCCC-3'
	Rv	5'-CTGTTGCTTAATTTGACCATTTATG-3'
pCaJAZ8	Fw	5'-CGTAATTGCCCTTGACAATGGTC-3'
	Rv	5'-GTAGTCTGTGATATTTGACATTTC-3'
pCaPR4m1W2W	Fw	5'-AGAGATCCAATTAGATAAATGTCCC-3'
	Rv	5'-CTGTTGCTTAATTCTCTCATTTATG-3'
pCaJAZ8m1W2W	Fw	5'-CGTAATTGCCCGAGAGAATAGAG-3'
	Rv	5'-GATGTCTGTGATATTCTCTCTTTC-3'
		**Primer sequences used for construction of *Xcv∆xopS***
P1_∆xopS	Fw	5'-GCGGATCCATGGGAGATGTACAAATG-3‘
	Rv	5'-ACGGCAGCCGTCGTATTGCATATCGGCAATCTGGCG-3‘
P2_∆xopS	Fw	5'- CGCCAGATTGCCGATATGCAATACGACGGCTGCCGT-3‘
	Rv	5'- GTCGACTCAAGAAATGCCATCGCTG-3‘

**Supplementary Table S2 *continued*. Nucleotide sequences of gene-specific primers.**		
**Target gene**		**Primer sequences used for construction of *Xcv∆xopS(XopS-HA)***
XopS-HA	Fw	5'-GGATCCTATGCTTTAGAAAAGCATGGGAGATGTAC-3'
	Rv	5'-GGATCCTCATGCGTAGTCTGGCACATCATAAGGGTAAGAAATGCCAT CGCT-3'
		**Primer sequences used for cloning**
XopS (with stop)	Fw	5'-CACCGGAGATGTACAAATGGGAAACTG-3'
	Rv	5'-TCAAGAAATGCCATCGCTGGCGCCACC-3'
XopS (without stop)	Fw	5'-CACCAACAATGGGAGATGTACAAATGG-3'
	Rv	5'-AGAAATGCCATCGCTGGCGCCACCGCG-3'
HopX1 (without stop)	Fw	5'-CACCAACAATGAGAATTCACAGTGCTGGTC-3'
	Rv	5'-TCTTCGTGGAGGCATGCCTTTAGACG-3'
PopP2 (without stop)	Fw	5'-CACCACAAATGAAGGTCAGTAGCGCAAAC-3'
	Rv	5'-GTTGGTATCCAATAGGGAATCC-3'
NtWRKY40 (with stop)	Fw	5'-CACCATGGAATTTACAAGTTTAGTTGA-3'
	Rv	5'-TTATTTATCCGTGTGATTATTTGG-3'
NbWRKY40 (with stop)	Fw	5'-CACCATGGAATTCACAAGTTTAGTTGAT-3'
	Rv	5'-TTATTTATCCGTGTGATTATTTGG-3'
NbWRKY40 (without stop)	Fw	5'-CACCACAAATGGAATTCACAAGTTTAGTTGAT-3
	Rv	5'-TTTATCCGTGTGATTATTTGGTAGG-3'
NbWRKY40a (with stop)	Fw	5'-CACCATGGAATTCACAAGTTTTGTTG-3'
	Rv	5'-TTACCATCTACTTGTTTGATTATTATG-3'
NbWRKY40e (with stop)	Fw	5'-CACCATGAATACAAGTTCTGGGGAGAAG-3'
	Rv	5'-TTAATCATATTCAAAAAAATTCCCGG-3'
NbWRKY8 (with stop)	Fw	5'-CACCGCAGCTTCTTCAACAATCATA-3'
	Rv	5'-TCAGCAGAGCAATGTCTCCATAAACAT-3'
NbWRKY8 (without stop)	Fw	5'-TTCATTTGGAGAGGACAGGAATGGCAGCTTCTTCAACAATC-3'
	Rv	5'-ATCCCCGGGTACCGAATTCACAGAGCAATGTCTCCATAAAC-3'
AtWRKY40 (with stop)	Fw	5'-GAATTCATGGATCAGTACTCATCCTCT-3'
	Rv	5'-GTCGACCTATTTCTCGGTATGATTCTG-3
CaWRKY40a (with stop)	Fw	5'- CACCAACAGAATTCACCAGTTTAG -3'
	Rv	5'-ATTTTTGTCTGTATGATTATTTTGTAAG-3'
CaWRKY40 (with stop)	Fw	5'-CACCATGGAATTTACCAGTTTGGTTGATAC-3'
	Rv	5'-TTACCATCTGCCCGTCTGATTATTATG-3'
CaWRKY1 (with stop)	Fw	5'-CACCATGGCTGCAAATAATCCCAGTGC-3'
	Rv	5'-TCAGAATGGGGATAGACCTTGATGG-3'
NbWRKY40 (VIGS)	Fw	5'-AGACCTCTTCGAGTTCCTGA-3'
	Rv	5'-GTGGCAATATTTCATTATTCTCAG-3'
CaWRKY40a (VIGS)	Fw	5'-ATGGAATTCACCAGTTTAGTTGATA-3'
	Rv	5'-GTGGATTATCAGCTGCAGTACTAGT-3'
CaWRKY40 (VIGS)	Fw	5'-TTCTGTGAGTAAGGTTACCGCCTTGACCCTGCACTATC-3'
	Rv	5'-CCCATGGAGGCCTTCTAGAGATGGAATTTACCAGTTTGGTTG-3'
pCaPR4 (GUS assay)	Fw	5'- CGTCTTTGATCGCACTAGTGAAGGTGACTTGGCACAGC-3'
	Rv	5'- TGTTGGATCCCCGGGTACCGCTACACACAACTTGTTAACACTC-3'
