# Supplementary material for: The Xanthomonas type-III effector XopS stabilizes CaWRKY40a to regulate defense responses and stomatal immunity in pepper (Capsicum annuum)
Source: Plant Cell. 2022 Feb 2;34(5):1684–708. doi: 10.1093/plcell/koac032 (PMC9048924; doi:10.1093/plcell/koac032)
Supplement: koac032_supplementary_data [file koac032_supplementary_data.zip › tpc.21.00991_Supplemental File S2.pdf]

Supplemental File 2. ANOVA and Student's t-test Tables

SS = Sum of Squares; DF = Degrees of Freedom; MS = Mean Squares

Figure 1A.

	SS	DF	MS	F-value (DF_n, DF_d)	P-value
Treatment (between columns)	13.11	2	6.554	F (2, 45) = 12.87	P < 0.0001
Residual (within columns)	22.91	45	0.5091		
Total	36.02	47			

Figure 1C.

	SS	DF	MS	F-value (DF_n, DF_d)	P-value
Treatment (between columns)	34286	3	11429	F (3, 28) = 36.62	P < 0.0001
Residual (within columns)	8738	28	312.1		
Total	43023	31			

Figure 1D.

	SS	DF	MS	F-value (DF_n, DF_d)	P-value
Treatment (between columns)	1.373	5	0.3432	F (4, 503) = 13.10	P < 0.0001
Residual (within columns)	13.18	503	0.02620		
Total	14.55	507			

Figure 2A. XopS-GFP #3

	SS	DF	MS	F-value (DF_n, DF_d)	P-value
Treatment (between columns)	15.94	3	5.313	F (3, 33) = 11.27	P < 0.0001
Residual (within columns)	15.56	33	0.4716		
Total	31.50	36			

Figure 2A. XopS-GFP #4

	SS	DF	MS	F-value (DF_n, DF_d)	P-value
Treatment (between columns)	6.296	3	2.099	F (3, 32) = 11.84	P < 0.0001

Residual (within columns)	5.674	32	0.1773		
Total	11.97	35			

Figure 2A. XopS-GFP #5

	SS	DF	MS	F-value (DF_n, DF_d)	P-value
Treatment (between columns)	9.884	3	3.295	F (3, 44) = 8.519	P = 0.0001
Residual (within columns)	17.02	44	0.3867		
Total	26.90	47			

Figure 2C. *AtMYC2*

P-value: 0.0043; Two-tailed; t = 3.936; DF = 8

Figure 2C. *AtJAZ10*

P-value: 0.0045; Two-tailed; t = 3.907; DF = 8

Figure 2D.

	SS	DF	MS	F-value (DF_n, DF_d)	P-value
Treatment (between columns)	0.4568	3	0.1523	F (3, 16) = 48.84	P < 0.0001
Residual (within columns)	0.04988	16	0.003118		
Total	0.5067	19			

Figure 2E.

	SS	DF	MS	F-value (DF_n, DF_d)	P-value
Treatment (between columns)	0.3199	3	0.1066	F (3, 396) = 6.141	P = 0.0004
Residual (within columns)	6.877	396	0.01737		
Total	7.197	399			

Figure 2F.

	SS	DF	MS	F-value (DF_n, DF_d)	P-value
Treatment (between columns)	9.302	2	4.651	F (2, 45) = 16.22	P < 0.0001
Residual (within columns)	12.90	45	0.2867		
Total	22.20	47			

Figure 2G.

	SS	DF	MS	F-value (DF_n, DF_d)	P-value

Treatment (between columns)	0.7158	3	0.2386	F (3, 426) = 23.64	P < 0.0001
Residual (within columns)	4.299	426	0.01009		
Total	5.015	429			

Figure 6A. *CaWRKY40a*

P-value: < 0.0001; Two-tailed; t = 9.580; DF = 12

Figure 6B. *CaWRKY40*

P-value: 0.8342; Two-tailed; t = 0.2162; DF = 8

Figure 6D.

P-value: < 0.0001; Two-tailed; t = 6.943; DF = 10

Figure 6E.

P-value: < 0.0001; Two-tailed; t = 7.347; DF = 26

Figure 7C.

	SS	DF	MS	F-value (DF_n, DF_d)	P-value
Treatment (between columns)	15575517	2	7787759	F (2, 9) = 75.64	P < 0.0001
Residual (within columns)	926619	9	102958		
Total	16502136	11			

Figure 7D. *CaCDPK15*

P-value: 0.0033; Two-tailed; t = 3.953; DF = 9

Figure 7D. *CaPR4*

P-value: 0.0062; Two-tailed; t = 3.547; DF = 9

Figure 7D. *CaJAZ8*

P-value: 0.0044; Two-tailed; t = 3.770; DF = 9

Figure 7D. *CaPR1*

P-value: 0.0347; Two-tailed; t = 2.486; DF = 9

Figure 8A. *CaWRKY40a*

P-value: 0.0005; Two-tailed; t = 6.896; DF = 6

Figure 8B.

	SS	DF	MS	F-value (DF_n, DF_d)	P-value
Treatment (between columns)	4.543	9	0.5048	F (9, 1406) = 15.35	P < 0.0001
Residual (within columns)	46.23	1406	0.03288		
Total	50.77	1415			

Figure 8C.

P-value: < 0.0001; Two-tailed; t = 6.718; DF = 14

Figure 8D. *CaWRKY40*

P-value: < 0.0001; Two-tailed; t = 9.533; DF = 6

Figure 8E.

	SS	DF	MS	F-value (DF_n, DF_d)	P-value
Treatment (between columns)	3.252	9	0.3613	F (9, 1167) = 15.51	P < 0.0001
Residual (within columns)	27.18	1167	0.02329		
Total	30.43	1176			

Figure 8F. *CaWRKY40*

P-value: 0.0003; Two-tailed; t = 5.466; DF = 10

Figure 8G.

P-value: 0.8991; Two-tailed; t = 0.1283; DF = 22

Figure 9A. *NbWRKY40*

P-value: 0.0020; Two-tailed; t = 5.207; DF = 6

Figure 9B. *NbWRKY40a*

P-value: 0.1847; Two-tailed; t = 1.425; DF = 10

Figure 9B. *NbWRKY40e*

P-value: 0.1483; Two-tailed; t = 1.566; DF = 10

Figure 9C.

	SS	DF	MS	F-value (DF_n, DF_d)	P-value
Treatment (between columns)	2.761	5	0.5522	F (5, 597) = 18.37	P < 0.0001
Residual (within columns)	17.94	597	0.03005		
Total	20.70	602			

Figure 9D. *NbWRKY40*

P-value: 0.0002; Two-tailed; t = 7.780; DF = 6

Figure 9E.

	SS	DF	MS	F-value (DF_n, DF_d)	P-value
Treatment (between columns)	2.524	5	0.5048	F (5, 639) = 19.47	P < 0.0001
Residual (within columns)	16.57	639	0.02592		
Total	19.09	644			

Figure 11B.

Data were transformed by Tukey's Ladder of Powers approach.

"###Descriptive Statistics"

I1_I2 N mean sd se

1 EV-HA - mit_XopS 6 127.16741 88.40379 36.090697

2 EV-HA - ohne_XopS 6 1446.85237 399.83046 163.230101

3 NbWRKY40-HA - mit_XopS 6 36.79798 17.51841 7.151862

4 NbWRKY40-HA - ohne_XopS 6 238.43583 13.30632 5.432281

5 NbWRKY8-HA - mit_XopS 6 345.36195 174.42306 71.207918

6 NbWRKY8-HA - ohne_XopS 6 2513.57182 648.01558 264.551252

Tukey's Ladder of Powers Transformation

Conducts Tukey's Ladder of Powers on a vector of values to produce a more-normally distributed vector of values.

lambda W Shapiro.p.value

400 -0.025 0.9506 0.1096

lambda

-0.025

```
if (lambda >  0){TRANS = x ^ lambda}
if (lambda == 0){TRANS = log(x)}
if (lambda <  0){TRANS = -1 * x ^ lambda} <= !!!
```

One Way AOV

Analysis of Variance Table

```
Response: df_sub_trans
          Df   Sum Sq   Mean Sq F value    Pr(>F)
I1_I2      5 0.035726 0.0071452  87.159 < 2.2e-16 ***
Residuals 30 0.002459 0.0000820
---
Signif. codes:  0 ‘***’ 0.001 ‘**’ 0.01 ‘*’ 0.05 ‘.’ 0.1 ‘ ’ 1
```

The ANOVA (formula: df_sub_trans ~ I1_I2) suggests that:

- The main effect of I1_I2 is statistically significant and large (F(5, 30) = 87.16, p < .001; Eta2 = 0.94, 95% CI [0.89, 1.00])

Effect sizes were labelled following Field's (2013) recommendations.

"### Check AOV Conditions"

"# TEST FOR NORMALITY"

Shapiro-Wilk normality test

data: aov_residuals

W = 0.96247, p-value = 0.256

-> p > 0.05 -> Residuals are normal distibuted

[1] "# HOMOGENITY of Variance"

Levene's Test for Homogeneity of Variance (center = median)

Df F value Pr(>F)

group 5 1.2076 0.3295

30

-> p > 0.05 Variance is homogeneous

=> Condition for performing an ANOVA are met

"###Descriptive Statistics"

	I1_I2	N	mean	sd	se
1	EV-HA - mit_XopS	6	127.16741	88.40379	36.090697
2	EV-HA - ohne_XopS	6	1446.85237	399.83046	163.230101
3	NbWRKY40-HA - mit_XopS	6	36.79798	17.51841	7.151862
4	NbWRKY40-HA - ohne_XopS	6	238.43583	13.30632	5.432281
5	NbWRKY8-HA - mit_XopS	6	345.36195	174.42306	71.207918
6	NbWRKY8-HA - ohne_XopS	6	2513.57182	648.01558	264.551252

Tukey's Ladder of Powers Transformation

Conducts Tukey's Ladder of Powers on a vector of values to produce a more-normally distributed vector of values.

	lambda	W	Shapiro.p.value
400	-0.025	0.9506	0.1096

lambda

-0.025

```
if (lambda >  0){TRANS = x ^ lambda}
if (lambda == 0){TRANS = log(x)}
if (lambda <  0){TRANS = -1 * x ^ lambda} <= !!!
```

One Way AOV

Analysis of Variance Table

Response: df_sub_trans

Df Sum Sq Mean Sq F value Pr(>F)

I1_I2   5 0.035726 0.0071452 87.159 < 2.2e-16 ***

Residuals 30 0.002459 0.0000820

Signif. codes: 0 ‘***’ 0.001 ‘**’ 0.01 ‘*’ 0.05 ‘.’ 0.1 ‘ ’ 1

The ANOVA (formula: df_sub_trans ~ I1_I2) suggests that:

  - The main effect of I1_I2 is statistically significant and large (F(5, 30) = 87.16, p < .001; Eta2 = 0.94, 95% CI [0.89, 1.00])

Effect sizes were labelled following Field's (2013) recommendations.

"### Check AOV Conditions"

"# TEST FOR NORMALITY"

Shapiro-Wilk normality test

data: aov_residuals

W = 0.96247, p-value = 0.256

-> p > 0.05 -> Residuals are normal distibuted

[1] "# HOMOGENITY of Variance"

Levene's Test for Homogeneity of Variance (center = median)

      Df F value Pr(>F)

group 5 1.2076 0.3295

      30

-> p > 0.05 Variance is homogeneous

=> Condition for performing an ANOVA are met

Posthoc test

Tukey-HSD

	df_sub_trans	groups
NbWRKY8-HA - ohne_XopS	-0.8228365	a
EV-HA - ohne_XopS	-0.8344807	a
NbWRKY8-HA - mit_XopS	-0.8664744	b

NbWRKY40-HA - ohne_XopS   -0.8721275   b

EV-HA - mit_XopS   -0.8892847   c

NbWRKY40-HA - mit_XopS   -0.9162271   d

Supplementary Figure S1A.

P-value: 0.8067; Two-tailed; t = 0.2461; DF = 46

Supplementary Figure S1D.

	SS	DF	MS	F-value (DF_n, DF_d)	P-value
Treatment (between columns)	16.64	2	8.318	F (2, 15) = 58.09	P < 0.0001
Residual (within columns)	2.148	15	0.1432		
Total	18.78	17			

Supplementary Figure S1E. *CaPR1*

	SS	DF	MS	F-value (DF_n, DF_d)	P-value
Treatment (between columns)	254233734	2	127116867	F (2, 11) = 141.2	P < 0.0001
Residual (within columns)	9906373	11	900579		
Total	264140107	13			

Supplementary Figure S2A.

	SS	DF	MS	F-value (DF_n, DF_d)	P-value
Treatment (between columns)	1.116	3	0.3718	F (3, 400) = 15.86	P < 0.0001
Residual (within columns)	9.376	400	0.02344		
Total	10.49	403			

Supplementary Figure S2B.

	SS	DF	MS	F-value (DF_n, DF_d)	P-value
Treatment (between columns)	0.8757	3	0.2919	F (3, 489) = 11.18	P < 0.0001
Residual (within columns)	12.77	489	0.02611		
Total	13.64	492			

Supplementary Figure S8. *CaWRKY40a*

P-value: 0.0227; Two-tailed; t = 2.691; DF = 10

Supplementary Figure S10. *NbWRKY40*

P-value: < 0.0001; Two-tailed; t = 11.18; DF = 22

Supplementary Figure S12.

P-value: 0.0918; Two-tailed; t = 1.915; DF = 8

Supplementary Figure S13A. *NbWRKY40*

P-value: < 0.0001; Two-tailed; t = 17.52; DF = 6

Supplementary Figure S13B.

	SS	DF	MS	F-value (DF_n, DF_d)	P-value
Treatment (between columns)	7876946	3	2625649	F (3, 20) = 62.45	P < 0.0001
Residual (within columns)	840818	20	42041		
Total	8717764	23			
